# Quality control and non-clinical testing of iPSC- and ESC-derived medicinal products: A systematic review

**DOI:** 10.1016/j.stemcr.2026.103084

**Published:** 2026-09-24

**Authors:** Akvilė Gasiūnaitė, Mara Tihaya, Inge van Amerongen-Westra, Fong Lin, Pema Gurung, Bahareh Rajaei, Eelco de Koning, Françoise Carlotti, Pauline Meij

**Affiliations:** 1 Center for Cell and Gene Therapy, Leiden University Medical Center, Leiden, the Netherlands; 2 Department of Internal Medicine, Leiden University Medical Center, Leiden, the Netherlands; 3 Walaeus Library, Leiden University Medical Center, Leiden, the Netherlands

**Keywords:** induced pluripotent stem cells, embryonic stem cells, cell therapy, regenerative medicine, quality control, non-clinical testing, release testing, characterization, safety and efficacy, systematic review

## Abstract

The lack of specific resources for quality control (QC) and non-clinical testing of cell-based medicinal products derived from embryonic and induced pluripotent stem cells (PSCs) contributes to uncertainty, variability, and delay in devising testing strategies. We conducted a systematic review on testing practices for diverse PSC-derived medicinal products using a custom-made ranking system, compiling a comprehensive “QC menu” of relevant critical quality attributes, established fit-for-purpose and promising techniques, and study designs for product development or clinical release. This consolidated resource could benefit product developers and regulators by creating informative testing strategies, ultimately increasing harmonization, facilitating clinical translation, and ensuring patient safety.

## Graphical abstract

**Figure:**
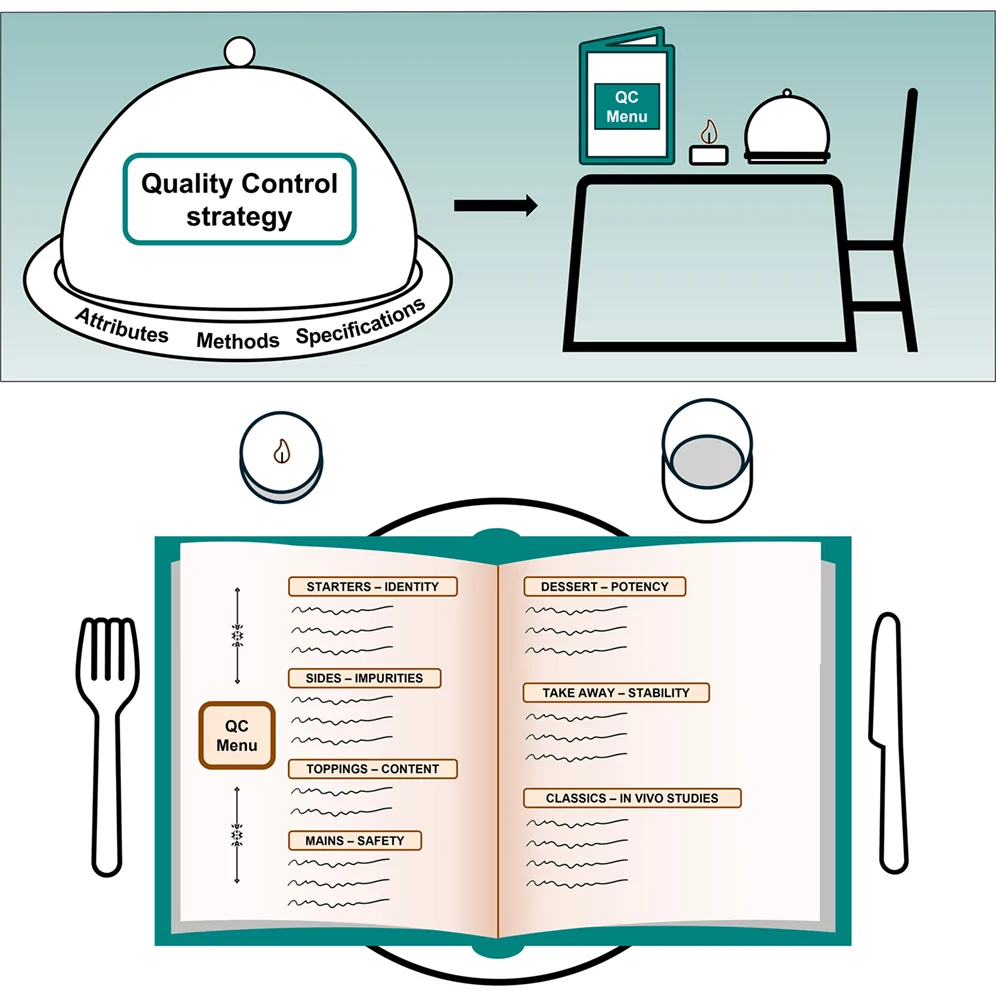

## Teaser

In this article, Meij and colleagues present a systematic review on quality control (QC) and non-clinical testing performed for iPSC- and ESC-derived therapies. This review serves as a resource informing product developers and regulators about critical quality attributes, methods, and study designs previously used to assess this type of medicinal products during development and clinical release.

## Introduction

Advanced therapy medicinal products are medicines mostly based on living cells and tissues. Increasingly, they are generated by differentiating pluripotent stem cells (PSCs), such as embryonic stem cells (ESCs) and induced PSCs (iPSCs) (Kirkeby et al., 2025; Kobold et al., 2023), which offer a renewable source of starting material for manufacturing. PSC-derived cell medicinal products were first introduced into the clinic 16 years ago (McKenna et al., 2022), and an increase of initiated clinical trials is observed in the last quinquennium, exceeding 100 studies in 2024 (Kirkeby et al., 2025).

PSC-derived therapies need to be thoroughly tested during pre-clinical development, manufacturing, and release to ensure quality, efficacy, and safety. Physical, chemical, or (micro)biological properties of a drug substance that describe and/or influence quality of the (investigational) medicinal product are called critical quality attributes (CQAs) (ICH, 2009). CQAs should meet appropriate specifications monitored via a set of quality control (QC) tests, which are performed on each product batch prior to release (ICH, 2009). While appropriate CQAs, acceptance criteria, and standardized testing methods are well-established in guidelines for conventional chemical pharmaceuticals (ICH, 1999), there are few detailed resources that can be consulted when designing a testing strategy for PSC-derived therapies (Barry et al., 2026; Hirai et al., 2023; Sato et al., 2019). Guidance for testing cell banks, including PSCs, has been established in regulatory guidelines (ICH, 1997, ICH, 2023) and consensus workshops (Sullivan et al., 2018); however, recommendations for testing PSC-derived end-products are less clearly defined (Barry et al., 2026; EMA, 2011; Hayakawa et al., 2015a; 2015b; 2015c), with the exception of compendial microbiological sterility, mycoplasma, and endotoxin tests, which are common across all aseptically produced medicinal products (EDQM (European Directorate for the Quality of Medicines & HealthCare) 2025a, EDQM (European Directorate for the Quality of Medicines & HealthCare) 2025b, EDQM (European Directorate for the Quality of Medicines & HealthCare) 2025c, EDQM (European Directorate for the Quality of Medicines & HealthCare) 2025d, EDQM (European Directorate for the Quality of Medicines & HealthCare) 2025e). A recently published document developed by the International Society for Stem Cell Research (ISSCR) has synthesized a more specific translational roadmap, taking into account the guidelines from major regional and international regulatory authorities, aiming to highlight the appropriate CQAs and suitable testing strategies for determining identity, purity, safety, potency, and stability of PSC-derived medicinal products (Barry et al., 2026).

The lack of specific guidance for PSC-derived therapies stems from the novelty, complexity, and diversity of these versatile products (Heslop et al., 2015). Although progressive insights on suitable testing strategies are gained with every new product entering clinical trials, the translatability of a QC strategy from one PSC-derived product to another is often limited due to differences in target cell types and product characteristics, necessitating custom development of quality assurance measures. Regulators evaluate the quality testing strategies on a case-by-case basis, relying on a risk-based approach to tailor the design of QC tests to the specific characteristics of the product (EMA, 2008). It is important for both the product developers and the regulators to have a comprehensive overview of the latest developments in the PSC-derived therapy field to design and evaluate suitable QC and non-clinical testing strategies for a successful translation of the product to the clinic.

Our paper focuses on the end-differentiated PSC derivatives and provides an in-depth overview of the currently applied practices for QC and non-clinical testing of the final products. We conducted a systematic search, extracting quality testing strategies and key parameters in the non-clinical study design implemented for ESC- and iPSC-derived products in the period of 2022–2024 to reflect the practices within a recent period in this fast-paced and innovative field. We used a custom-made ranking system to discriminate tests used for product characterization during the pre-clinical developmental phase, and tests that either have been used or were proposed as a QC release test in a clinical setting. Our aim was to systematically map the quality testing practices applied for PSC-derived medicinal products and to develop a ranked “QC menu” of methods with proven and promising suitability to assess a wide range of CQAs. This comprehensive resource of existing QC and non-clinical testing practices can thus complement the existing guidelines with a list of applicable techniques and support the design of future testing strategies, leading to higher quality PSC-derived medicinal products and, ultimately, patient safety.

## Results

### General characteristics of included reports

Fifty reports were included from 2022 to 2024, covering a variety of ESC- and iPSC-derived products developed for diverse disease indications (Bershteyn et al., 2023; Chung et al., 2022; Cichocki et al., 2022; Corsini and Mitola, 2023; Diaz-Hernandez et al., 2023; Domingues et al., 2022; Fessler et al., 2022; Gantier et al., 2024; Gotkine et al., 2023; Hirami et al., 2023; Huang et al., 2023; Iworima et al., 2024; Izrael et al., 2018; Jo et al., 2022; Kirkeby et al., 2023; Kobayashi et al., 2024; Kriedemann et al., 2024; Lemmens et al., 2023; Luciani et al., 2022; Manley et al., 2017; Masumoto et al., 2022; Miyagawa et al., 2022; Miyagawa et al., 2024; Naderi et al., 2023; Neumayer et al., 2024; Oh et al., 2022; Park et al., 2024a, Park et al., 2024b; Rong et al., 2024; Sekine et al., 2023; Shi et al., 2022; Shigyo et al., 2023; Shin et al., 2022; Soma et al., 2024; Sugimoto et al., 2022a, Sugimoto et al., 2022b; Sun et al., 2024; Surendran et al., 2021, Surendran et al., 2022; Tsujimoto et al., 2022; Wang et al., 2024; Watanabe et al., 2023, 2024; Watari et al., 2023; Wu et al., 2020, 2023, 2024; Yasuda et al., 2024; Yu et al., 2023; Zhao and Liu, 2022) (Figure S1A; Table S1). In this dataset, reports on neuronal cells, cardiomyocytes, and mesenchymal stromal/stem cells (MSCs) collectively accounted for approximately half of all entries, underscoring the prominence of these medicinal products in current research. Four reports were designed to develop universal techniques that could be applicable for products of multiple lineages (Corsini and Mitola, 2023; Masumoto et al., 2022; Oh et al., 2022; Shi et al., 2022). Furthermore, almost half of the reports described products or techniques that were used in clinical trials (Figure S1B) in Asia (13 unique clinical trials) (Gotkine et al., 2023; Hirami et al., 2023; Huang et al., 2023; Izrael et al., 2018; Kobayashi et al., 2024; Miyagawa et al., 2022, 2024; Park et al., 2024b; Rong et al., 2024; Shin et al., 2022; Soma et al., 2024; Sugimoto et al., 2022a, 2022b; Surendran et al., 2021, 2022; Wang et al., 2024; Watari et al., 2023; Wu et al., 2020, 2024), the United States (3) (Bershteyn et al., 2023; Cichocki et al., 2022; Fessler et al., 2022; Manley et al., 2017), and Europe (1) (Kirkeby et al., 2023). The remaining reports focused on technique validation, development of manufacturing protocols and QC assays, and product characterization to support clinical translation (Chung et al., 2022; Corsini and Mitola, 2023; Diaz-Hernandez et al., 2023; Domingues et al., 2022; Gantier et al., 2024; Iworima et al., 2024; Jo et al., 2022; Kriedemann et al., 2024; Lemmens et al., 2023; Luciani et al., 2022; Masumoto et al., 2022; Naderi et al., 2023; Neumayer et al., 2024; Oh et al., 2022; Park et al., 2024a; Sekine et al., 2023; Shi et al., 2022; Shigyo et al., 2023; Sun et al., 2024; Tsujimoto et al., 2022; Watanabe et al., 2023, 2024; Wu et al., 2023; Yasuda et al., 2024; Yu et al., 2023; Zhao and Liu, 2022).

### Identity

Tests used to identify and quantify the target cell population were grouped according to the investigated CQA and scored in accordance with the created ranking system (Tables 1 and S2; Figure 1A). The ranking system was based on terms widely used for testing of medicinal products: characterization performed as extensive testing for establishing product and process understanding during product development (Ch); QC testing for decision-making purposes during release ensuring quality of the medicinal product (QC) (ICH, 2008); and supportive testing during the release of the product performed For Information Only (FIO) (Madrid et al., 2024). The ranking was determined based on the application of a testing method as described in a report. Release tests for clinical application with or without defined specifications were ranked as “QC” or “QCu” (undefined), respectively. FIO tests used for the release of a product for clinical application were ranked “FIO”, whereas potential/proposed QC and FIO tests for intended clinical application were ranked “pQC” and “pFIO”. Lastly, tests used for the characterization during product development, but not intended for use as release tests, were ranked “Ch” (Table 1).

**Table 1:** Ranking system of quality tests

Rank	Definition
QC	A test used as a QC for release of a product for clinical application with specific acceptance criteria.
QCu (QC undefined)	A test used as a QC for release of a product for clinical application, however, the specific acceptance criteria are not mentioned (e.g., markers for target cell identity were defined and results were given but not the acceptance criteria).
FIO	A test used during the release of a product for clinical application For Information Only.
pQC (proposed/potential QC)	A test/property defined as a QC/CQA of a product but not employed in the product release for a clinical application yet. It may not have the specific acceptance criteria.
pFIO (proposed/potential FIO)	A test proposed as optional yet supportive but not employed in the product release for clinical application yet.
Ch (characterization)	A test used during the development and characterization of the product and not falling under the definitions above.

**Figure 1:**
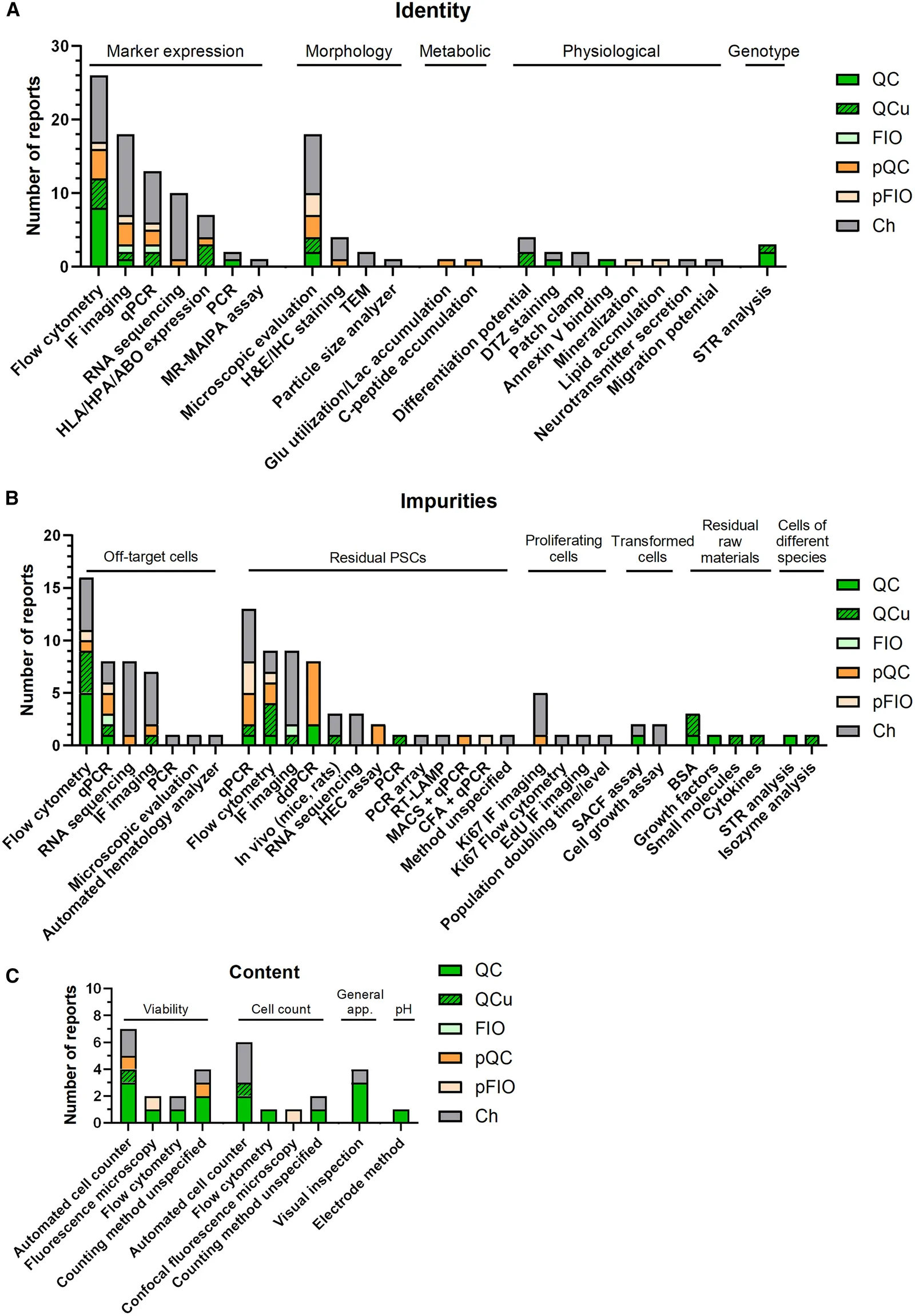
Identity, purity, and content evaluation of PSC-derived therapy products (A) Target cell identity was confirmed by various techniques assessing marker expression, morphology, metabolic and physiological properties, and genotype. The assessment of the HLA, HPA, or ABO blood type antigens was performed by flow cytometry but was grouped as a separate method in identity testing due to the focus on immunophenotypic cell profile. See also Table S2. (B) Techniques used to detect and/or quantify cellular and acellular impurities such as off-target cells; tumorigenic cells including residual PSCs, proliferating, and transformed cells; residual raw materials; and contaminating cells of different species. See also Figure S2; Tables S3 and S4. (C) Content of the end formulation was evaluated based on cell viability and count, general appearance of cell suspension and sheets, and pH. The unspecified counting methods mostly relied on Trypan Blue exclusion and fluorescent staining. See also Table S5. BSA, bovine serum albumin; CFA, colony formation assay; ddPCR, droplet digital PCR; DTZ, dithizone; EdU, 5-ethynyl-2′-deoxyuridine; Glu, glucose; HEC: highly efficient culture; HLA, human leukocyte antigen; HPA, human platelet antigen; H&E, hematoxylin and eosin; IF, immunofluorescence; IHC, immunohistochemistry; Lac, lactate; MACS, magnetic-activated cell sorting; MR-MAIPA, modified rapid monoclonal antibody-specific immobilization of platelet antigens; RT-LAMP, reverse transcription loop-mediated isothermal amplification; SACF, soft agar colony formation; STR, short tandem repeat; TEM, transmission electron microscopy.

Marker expression was the most frequently evaluated cell attribute with flow cytometry as the leading technique utilized to determine the identity of the product, both for general characterization and release testing (Figure 1A). PCR techniques and immunofluorescence (IF) imaging were used to analyze RNA and protein expression, respectively, both qualitatively, by gel electrophoresis or observing positive staining, as well as quantitatively, by qPCR and counting positive cells. Bulk (Naderi et al., 2023; Neumayer et al., 2024; Surendran et al., 2021; Wu et al., 2020; Yu et al., 2023) and single cell (Bershteyn et al., 2023; Jo et al., 2022; Miyagawa et al., 2024; Neumayer et al., 2024; Wang et al., 2024; Wu et al., 2020, 2024) RNA sequencing was mostly used for characterization; however, one report has proposed to use single cell RNA sequencing for QC assessment (Jo et al., 2022).

The next CQA that was most often taken into consideration was cell or aggregate morphology. The most common method was microscopic evaluation without staining to observe the morphology and size of individual cells or cell clusters. Hematoxylin and eosin (H&E) and immunohistochemistry (IHC) staining were used to assess tissue-engineered constructs for presence of cell multilayers, subcellular structures, or cell polarization. Transmission electron microscopy was used to observe platelet ultrastructures (Sugimoto et al., 2022a) and subcellular structures and cell-cell connections in cardiomyocyte patches (Miyagawa et al., 2024), while a particle size analyzer was employed to evaluate platelet maturity (Sugimoto et al., 2022a).

Metabolic parameters have been proposed as a CQA in pancreatic islet cells, where the consumption of nutrients and production of certain metabolites were suggested to indicate the required switch toward the intended cell type (Iworima et al., 2024). Specifically, decreasing glucose consumption and lactate accumulation rates indicated the transition from glycolysis to oxidative phosphorylation during the differentiation of ESCs into pancreatic islets, while decreased C-peptide accumulation in spent medium at end differentiation stage could indicate the shift from primarily constitutive to more regulated hormone secretion, representing beta cell maturation (Iworima et al., 2024).

Several tests that were used to verify the cellular identity as a CQA were related to their physiology and therefore included a variety of methods to assess functional characteristics. For example, MSC identity was confirmed by trilineage (osteogenic, chondrogenic, and adipogenic) differentiation potential verified by cell morphology, IF imaging, and/or qPCR (Huang et al., 2023; Rong et al., 2024; Shin et al., 2022; Wu et al., 2020). This ability was evaluated to confirm the physiological identity of the product, rather than potency, as the intended therapeutic mechanism was not directly related to the differentiation capacity, but was based on the immunomodulatory properties of MSCs for the treatment of joint, lung, bladder, and retinal diseases (Huang et al., 2023; Rong et al., 2024; Shin et al., 2022; Wu et al., 2020).

Finally, donor identity confirmation is required to match the differentiated product to the correct source. For this CQA, the cell product’s genotype was compared with the Master Cell Bank and/or the patient/donor cells for allogeneic (Kirkeby et al., 2023; Wu et al., 2020) and autologous (Wang et al., 2024) products. This assessment was applied as a QC test by performing a short tandem repeat (STR) analysis using PCR technique in three reports (Kirkeby et al., 2023; Wang et al., 2024; Wu et al., 2020).

To sum up, target cell identity was confirmed in a variety of ways by assessing protein and RNA expression, cell and aggregate morphology, metabolism, and physiological characteristics, as well as a match with the donor material. While some techniques were product-specific (e.g., antigen recognition assay for platelets), others were universal and used as QC tests for a variety of product types (e.g., flow cytometry, bright-field and fluorescence microscopy, and qPCR).

### Impurities

The purity evaluation of PSC-derived products was based on identifying and quantifying cellular and acellular impurities, which can be divided into four subgroups: off-target cells, tumorigenic cells, residual raw materials, and interspecies contamination (Figure 1B; Table S3).

The most frequently assessed impurity was off-target cells, often detected using the same techniques as described in the “Identity” section. Additionally, to detect off-target tissue in retinal organoids, the cells from other lineages arising during the differentiation process and residual progenitors were detected using single-cell PCR and bright-field microscopy (Watari et al., 2023). For a platelet product, an automated hematology analyzer was used to quantify the immature platelet fraction (Sugimoto et al., 2022b).

The absence of undifferentiated PSCs in any PSC-derived medicinal product is considered an important CQA, as these cells can have tumorigenic potential (Sato et al., 2019). Considerable attention was given to methods fit for the purpose of identifying residual undifferentiated PSCs, which could be present in low quantities among the product cells, requiring assays with sufficiently low detection thresholds. Although the most frequent techniques largely overlapped with the ones used for off-target cell identification, such as qPCR, flow cytometry, and IF imaging, we observed increasing use of novel techniques specifically for assessment of this CQA using droplet digital PCR (ddPCR) (Chung et al., 2022; Shi et al., 2022; Soma et al., 2024; Sun et al., 2024; Tsujimoto et al., 2022; Wang et al., 2024; Wu et al., 2023; Yasuda et al., 2024) and highly efficient culture (HEC) assay (Watanabe et al., 2023, 2024). Whilst the ddPCR is also based on marker detection, it is proposed to offer a lower detection limit (0.0001%–0.001%, as specified in the included reports) than the more widely used qPCR (0.001%–0.38%) and flow cytometry (0.002%–0.2%) (Figure S2; Table S4). The HEC assay, based on culturing a sample of the product in PSC maintenance medium to detect outgrowing PSC colonies, has also achieved a low limit of detection (0.0005-0.005%) (Watanabe et al., 2023, 2024). The detection threshold of an established qPCR technique could be improved through a combination of methods to enrich the residual PSC population by magnetic-activated cell sorting (MACS) (Tsujimoto et al., 2022) or pre-culturing cells in PSC maintenance medium (Sekine et al., 2023) before applying the detection method. *In vivo* mouse and rat studies have also been used to confirm the absence of tumorigenicity and to determine the percentage of undifferentiated PSCs by comparing the teratoma formation of PSC-derived end-products to spike-in samples with known PSC concentrations for up to 52 weeks (Park et al., 2024b; Wu et al., 2020; Yu et al., 2023) (Figure 1B). Interestingly, even though animal studies are rarely used as release tests due to long duration, high costs, and the need for repeated testing for every batch, a 6-week-long *in vivo* mouse study was listed together with other quality tests required by the Chinese regulatory authority for one of the products (Wu et al., 2020).

Potentially tumorigenic cells may also arise during the manufacturing process. Although some degree of expansion is expected in the end-products containing immature progenitor cells, which can either be the intended on-target cell population or off-target cells remaining due to incomplete differentiation, excessive proliferation represents a potential risk that could result in uncontrolled growth and tumor formation. Proliferative capacity was assessed by IF imaging or flow cytometry using the Ki67 marker (Bershteyn et al., 2023; Gantier et al., 2024; Izrael et al., 2018; Park et al., 2024b; Surendran et al., 2021; Yu et al., 2023) or EdU incorporation (Bershteyn et al., 2023) and by measuring population doubling time and expansion level (Park et al., 2024b). Dysregulated proliferative activity could also be an indication of malignant transformation as cells may accumulate mutations and become tumorigenic. The presence of such cells was assessed using a soft agar colony formation (SACF) assay, which is based on detecting the outgrowth of anchorage-independent colonies in agar over 2–3 weeks (Miyagawa et al., 2024; Wang et al., 2024). Similarly, proliferation rate was monitored in a cell growth assay by culturing cells under regular passaging conditions in maturation medium for 5–6 passages up to 50 days (Miyagawa et al., 2024; Soma et al., 2024).

Acellular impurities originating from raw materials used during manufacturing and cryopreservation have been a part of the QC test panel in three reports (Sugimoto et al., 2022a; Wang et al., 2024; Wu et al., 2020). This included the detection of residual animal-derived components (bovine serum albumin), recombinant growth factors and cytokines, and small molecules (agonists, antagonists, inhibitors, antibiotics, and solvents) using methods such as ELISA, liquid chromatography-tandem mass spectrometry (LC-MSMS), and gas chromatography-mass spectrometry (GC-MS). Two reports tested for a possible contamination with cells of different species (Wang et al., 2024; Wu et al., 2020). The absence of interspecies contaminants was confirmed by STR analysis, simultaneously testing for the donor material identity, and by isozyme analysis relying on the electrophoretic mobility of enzyme isoforms, with human cells showing a distinct banding pattern (Chang et al., 2001).

To conclude, for the assessment of product purity as a CQA, different impurities were tested at varying frequencies, with off-target cells and residual PSCs being the most prevalent cellular impurities of interest. While flow cytometry, qPCR, and IF imaging remain the most frequently used techniques for QC, new methods are being developed and qualified, offering potentially superior detection thresholds. Culture-based methods can be suitable to detect tumorigenic cells of different origins.

### Content

Product content, reflecting composition and therapeutic dose in the final formulation, was evaluated as a separate quality category. Viability and total cell count were the most frequently assessed CQAs, measured on single-cell samples using automated cell counters or flow cytometry (Figures 1C; Table S5). Alternatively, (confocal) fluorescence microscopy of cell viability and membrane integrity assays has been proposed and applied for whole aggregates (Kriedemann et al., 2024; Wang et al., 2024), with one of the reports describing a procedure to validate the imaging assay (Kriedemann et al., 2024). General appearance by visual inspection has also been set as a QC criterium for product release assessing the color and turbidity of the cell suspension ensuring absence of foreign particles (Kirkeby et al., 2023; Sugimoto et al., 2022a) and macroscopically observing the integrity of cell sheets after detaching from culture vessels (Miyagawa et al., 2022; Soma et al., 2024). In one platelet product, pH was included as a release parameter (Sugimoto et al., 2022a), as in platelet concentrates it can indicate metabolic activity and viability (FDA, 2008; Murphy and Gardner, 1975). Overall, total and viable cell numbers, supported by visual appearance and, in some products, pH, represent key content-related parameters for confirming the quality of PSC-derived cell therapies.

### Safety

For the overview of quality testing related to safety, compendial sterility testing was excluded, and our analysis focused on the aspects specific to PSC-derived medicinal products such as genomic integrity, tumorigenicity, cytotoxicity, and adventitious virus presence.

#### Genomic integrity

Genomic integrity of the end products was assessed in 11 reports at different levels of detail (Bershteyn et al., 2023; Domingues et al., 2022; Kirkeby et al., 2023; Miyagawa et al., 2022, 2024; Neumayer et al., 2024; Park et al., 2024a; Shigyo et al., 2023; Shin et al., 2022; Soma et al., 2024; Wang et al., 2024). Chromosomal aberrations such as numerical variations or large structural abnormalities were evaluated by karyotyping 20-50 metaphases using Giemsa or multicolor fluorescence *in situ* hybridization (mFISH) staining (Domingues et al., 2022; Shigyo et al., 2023; Shin et al., 2022; Soma et al., 2024) (Figure 2A; Table S6). For the assessment of post-mitotic cells and a more detailed analysis of mutations in known high-risk genomic locations and structural variants associated with cancer or other diseases, several targeted approaches were employed. Copy number variations (CNVs) were identified by a comparative genomic hybridization (CGH) array (Miyagawa et al., 2022), while both CNVs and copy neutral loss of heterozygosity (CN-LOH) events could be detected by single nucleotide polymorphism (SNP) arrays (Bershteyn et al., 2023; Kirkeby et al., 2023; Miyagawa et al., 2024; Soma et al., 2024) (e.g., HumanOmniExpress-24 (Miyagawa et al., 2024; Soma et al., 2024) and HumanCytoSNP-12 (Kirkeby et al., 2023)), and a chromosomal microarray (CytoScanHD (Park et al., 2024a)).

**Figure 2:**
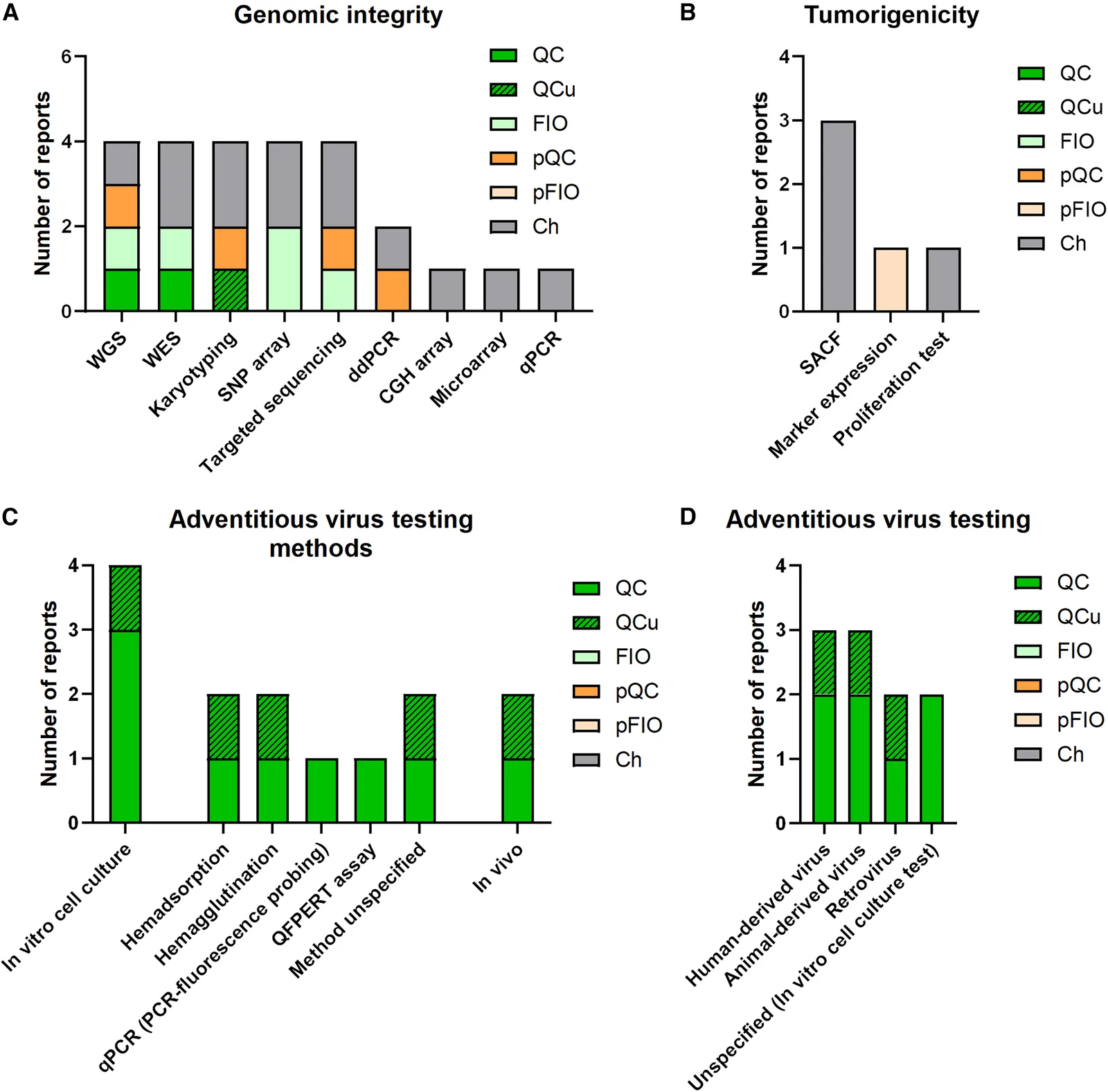
Safety testing of PSC-derived therapy products (A) Methods used to assess genomic integrity in the end-products. See also Table S7. (B) *In vitro* tests to determine the tumorigenic potential of product cells. (C) Methods for detecting adventitious viruses based on the use of detector cell lines, cell-free *in vitro* techniques, and *in vivo* studies. See also Figure S3A. (D) Virus types and species targeted for detection in adventitious virus testing. See also Figure S3B. For the whole figure, see also Table S6. CGH, comparative genomic hybridization; ddPCR, droplet digital PCR; QFPERT, quantitative fluorescent product-enhanced reverse transcriptase; SACF, soft agar colony formation; SNP, single nucleotide polymorphism; WES, whole-exome sequencing; WGS, whole-genome sequencing.

High-resolution next-generation sequencing (NGS) methods, capable of detecting single-base changes, were the most frequently used techniques for PSC-derived end-products, aiming to confirm the absence of (malignant) *de novo* mutations acquired during cell culture. Targeted sequencing was utilized in four reports using different cancer gene panels, covering a single gene (*TP53*) or up to 344 cancer-related genes (Bershteyn et al., 2023; Kirkeby et al., 2023; Neumayer et al., 2024; Park et al., 2024a) (Table S7). Alternatively, untargeted whole-genome sequencing (WGS) or whole-exome sequencing (WES) was done to acquire comprehensive information on CNVs, single-nucleotide variants (SNVs), insertions and deletions (INDELs). The sequencing depth reached approximately 40–83× for WGS (Miyagawa et al., 2024; Neumayer et al., 2024; Soma et al., 2024; Wang et al., 2024), 83–300× for WES (Miyagawa et al., 2024; Park et al., 2024a; Soma et al., 2024; Wang et al., 2024), and 100–300× for targeted sequencing (Bershteyn et al., 2023; Park et al., 2024a) (Table S7).

Results acquired by the different NGS and array methods were often analyzed by collating the identified variants to publicly available databases reporting on genetic variations related to tumorigenicity and other pathological conditions (Miyagawa et al., 2024; Park et al., 2024a; Soma et al., 2024; Wang et al., 2024). In addition, databases containing unannotated and benign genomic variants were cross-referenced to exclude constitutive variants unlikely to be related to disease (Kirkeby et al., 2023; Miyagawa et al., 2024; Neumayer et al., 2024). Moreover, various *in silico* tools were used to predict the deleterious nature of the captured variants (Park et al., 2024a) by evaluating the impact on the change in protein structure (Montenegro et al., 2021). Follow-up analysis using targeted ddPCR has been proposed as a QC test (Park et al., 2024a) and was used to detect mutated variants present at low frequencies previously identified through NGS (Neumayer et al., 2024; Park et al., 2024a), while qPCR was shown to be more prone to false-positive results (Park et al., 2024a) (Figure 2A).

Based on the used targeted panels and databases, the primary goal of assessing genomic integrity in the final PSC-derived medicinal products was to detect cancer-related variants and evaluate potential tumorigenicity of transformed cells. While karyotyping was frequently used where possible, high-resolution NGS and array-based techniques suitable for assessing genomic aberrations in post-mitotic PSC-derived cells are gaining popularity as QC and FIO tests.

#### *In vitro* tumorigenicity testing

Some methods previously described in the “Impurities” section were applied with the primary goal to evaluate the potential to form tumors rather than estimating levels of specific cellular impurities, and were therefore assigned to the “Safety” category (Gantier et al., 2024; Miyagawa et al., 2024; Sugimoto et al., 2022a, 2022b; Wu et al., 2020). Quantification of proliferating cell colonies was done in agarose (SACF assay) (Gantier et al., 2024; Sugimoto et al., 2022a; Wu et al., 2020), suspension culture (Sugimoto et al., 2022b), and adherent culture assays (Sugimoto et al., 2022b) for up to 21 days (Wu et al., 2020), 22 days (Sugimoto et al., 2022b), and 12 days (Sugimoto et al., 2022b), respectively (Figures 2B; Table S6). Master Cell Bank PSCs (Sugimoto et al., 2022a; Wu et al., 2020) and intermediate product cells (Sugimoto et al., 2022a, 2022b; Wu et al., 2020) served as comparators, with cancer cell lines (Sugimoto et al., 2022a, 2022b) or cancer cell spike-ins (Sugimoto et al., 2022b) as positive controls. One report proposed quantifying LIN28A marker expression levels as an alternative to *in vivo* tumorigenicity testing, as this marker had been found to correlate with tumor formation frequency in mice (Miyagawa et al., 2024).

#### *In vitro* toxicity testing

Two reports described the use of *in vitro* cell viability models to investigate potential cytotoxic effects of the product on surrounding healthy cells (Cichocki et al., 2022; Domingues et al., 2022). For genetically engineered NK cells, absence of off-target cytotoxicity was assessed by co-culturing the product with pre-labeled hematopoietic progenitor cells followed by viability assessment using flow cytometry (Cichocki et al., 2022). For engineered skin tissue, the toxicity of reconstituted composite keratinocyte-fibroblast skin grafts was assessed by applying the product’s conditioned medium to intact primary keratinocyte monolayers and recording apoptosis via live cell imaging (Domingues et al., 2022).

#### Adventitious virus testing

Adventitious viral agent testing, performed to detect viral contamination potentially present in the starting material or introduced through raw materials during manufacturing, was described in only five products, but applied as a QC release test in all these cases (Kirkeby et al., 2023; Park et al., 2024b; Wang et al., 2024; Wu et al., 2024, Wu et al., 2020) (Figures 2C and 2D; Table S6). Most reports described a combination of methods used to assess viral safety in the PSC-derived end products (Figure S3). *In vitro* cell culture assays were most frequently described, using co-cultures of cell product (or spent culture media) with indicator cell lines of human, monkey, bovine, and porcine origin (Kirkeby et al., 2023; Park et al., 2024b; Wang et al., 2024; Wu et al., 2020) to observe cytopathic effect (Kirkeby et al., 2023; Wang et al., 2024). Hemadsorption and hemagglutination tests were used independently or as follow-up methods for the *in vitro* cell culture tests (Wang et al., 2024; Wu et al., 2020). For the detection of known viral contaminants and retroviruses, qPCR (Wang et al., 2024) and quantitative fluorescent product-enhanced reverse transcriptase (QFPERT) assay (Kirkeby et al., 2023) were used, respectively. *In vivo* testing was performed in two reports, using various animal models (Wang et al., 2024; Wu et al., 2020).

All in all, the safety of the PSC-derived medicinal products was evaluated with regards to genomic integrity, tumorigenic outgrowth, cytotoxicity, and infectious viral agents. A number of *in vitro* approaches were used to determine the product safety profile and possible pathogenic, toxic, and infectious risks.

### Potency

Due to the variety of medicinal products that can be obtained from PSCs, approaches to examine their potency can be tailored to different characteristics and differ per product. Some of the potency tests aim to directly measure CQAs linked to the mechanism of action, while others rely on surrogate markers. However, distinguishing between direct or surrogate potency assays proved challenging due to the highly product- and indication-specific context defining the therapeutic mechanism of a PSC-derived medicinal product. Therefore, it was not possible to consistently and reliably classify this based on the limited information provided about potency development in the included reports. For these reasons, potency tests designed to evaluate the biological activity of a product were grouped based on the assessed characteristics rather than by method used and represent a combination of both direct and surrogate assays (Figure 3; Table S8).

**Figure 3:**
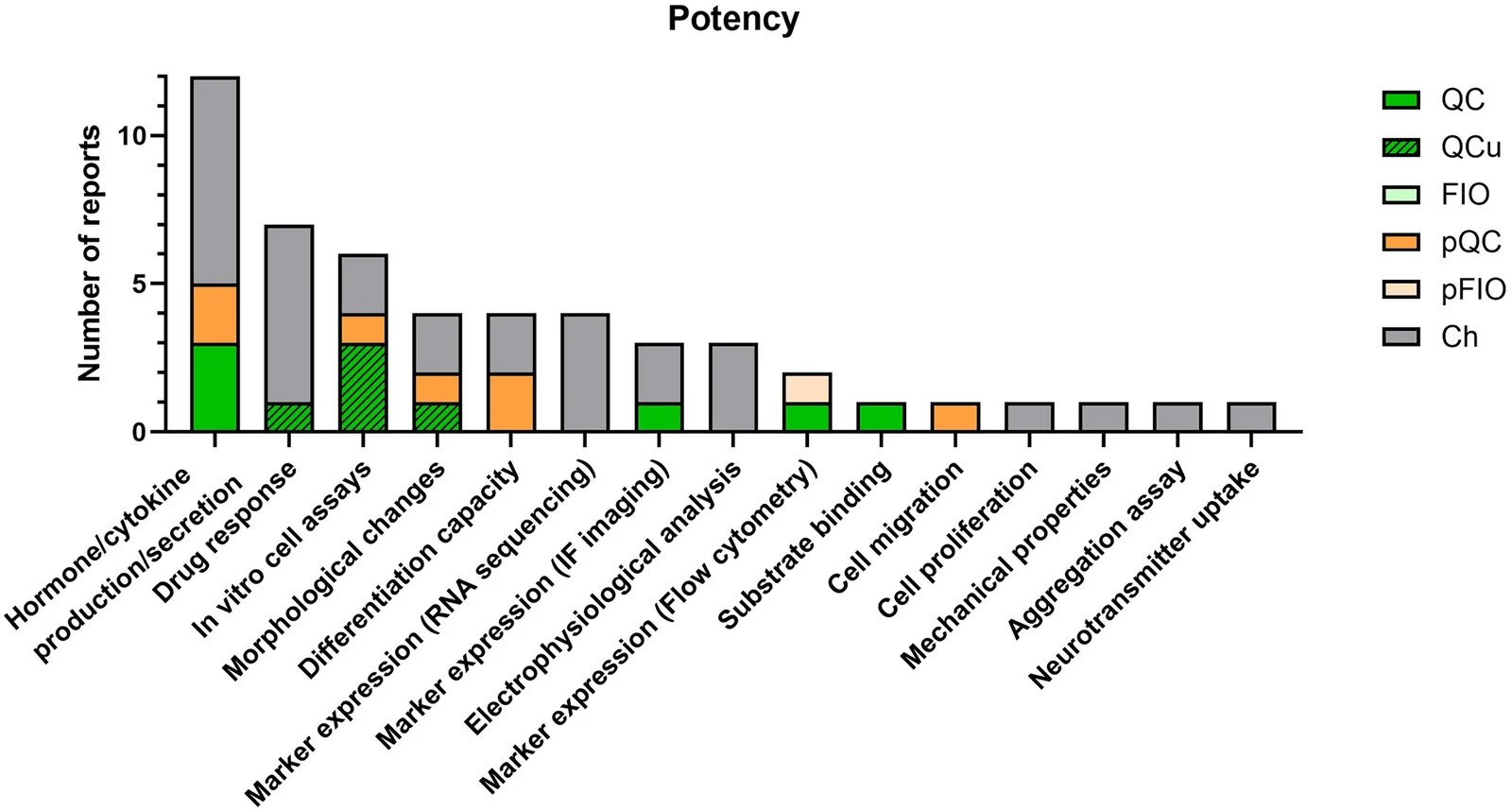
Potency tests of PSC-derived therapy products Functionality of the cells and tissue constructs was evaluated using a range of direct and surrogate assays based on various physiological properties, *in vitro* cell assays, and marker expression. See also Table S8. IF, immunofluorescence.

The most frequent approach to evaluate product potency was measuring production or secretion of cell-specific hormones and cytokines (Cichocki et al., 2022; Gantier et al., 2024; Gotkine et al., 2023; Huang et al., 2023; Iworima et al., 2024; Miyagawa et al., 2024; Naderi et al., 2023; Park et al., 2024b; Surendran et al., 2022; Wang et al., 2024; Wu et al., 2024, Wu et al., 2020). Signature molecules, such as cell-specific growth factors (Gantier et al., 2024; Gotkine et al., 2023; Miyagawa et al., 2024; Surendran et al., 2022), glucagon and insulin or its by-product C-peptide (Iworima et al., 2024; Wang et al., 2024; Wu et al., 2024), neurotransmitters (Naderi et al., 2023; Park et al., 2024b), and immunomodulatory cytokines (Cichocki et al., 2022; Huang et al., 2023; Wu et al., 2020), were detected using ELISA (Gantier et al., 2024; Gotkine et al., 2023; Huang et al., 2023; Iworima et al., 2024; Naderi et al., 2023; Park et al., 2024b; Surendran et al., 2022; Wang et al., 2024; Wu et al., 2024, Wu et al., 2020), radioimmunoassay (Iworima et al., 2024), mass spectrometry (Gantier et al., 2024), electrochemiluminescence (Cichocki et al., 2022), or bead-coupled multiplex immunoassays (Miyagawa et al., 2024). The release of the molecules was often assessed in response to a biologically relevant inducing factor, such as membrane depolarization by ion (Naderi et al., 2023; Park et al., 2024b) or glucose stimulation (Iworima et al., 2024; Wang et al., 2024; Wu et al., 2024), treatment with pro-inflammatory cytokines (Wu et al., 2020) or synovial fluid from meniscus injury patients (Huang et al., 2023), hypoxia induction (Miyagawa et al., 2024), or co-culture with a tumor cell line (Cichocki et al., 2022). In addition, hormone release was assessed to evaluate cell polarity by measuring the differential growth factor secretion in supernatant on the apical and basal side of the retinal pigment epithelium cell layer (Surendran et al., 2022). In rare cases, the secreted proteins were also assessed for their activity and correct isoform (Wu et al., 2020).

*In vitro* cell assays utilized model cells co-cultured with the medicinal product or its conditioned medium to assess the activity of the product. Stimulation of tissue regeneration was evaluated through chondrocyte migration (Huang et al., 2023) and keratinocyte scratch assay (Domingues et al., 2022). Immunomodulatory effects were observed by inhibited lymphocyte proliferation (Huang et al., 2023; Wu et al., 2020) and anti-fibrotic effects by reduced myofibroblast *trans*-differentiation (Wu et al., 2020). CAR-NK cell potency was measured by antigen-specific cytotoxicity against engineered cancer cells and persistence via serial restimulation (Cichocki et al., 2022). Astrocyte functionality was demonstrated by promoting axonal outgrowth and protecting neurons from oxidative stress (Izrael et al., 2018). Angiogenic interactions between MSCs and endothelial cells were assessed using a dye transfer assay (Shin et al., 2022).

Other CQAs related to potency that were interrogated included morphological changes and structural organization of cells (Domingues et al., 2022; Huang et al., 2023; Shin et al., 2022; Wu et al., 2020), differentiation capacity (Domingues et al., 2022; Naderi et al., 2023; Shigyo et al., 2023; Zhao and Liu, 2022), electrophysiological analyses (Miyagawa et al., 2024; Naderi et al., 2023; Yu et al., 2023), PAC-1 binding and aggregation for platelets (Sugimoto et al., 2022a), MSC migration toward tumor cells (Zhao and Liu, 2022), corneal epithelial cell proliferation (Soma et al., 2024), mechanical strength of cardiomyocyte patches (Miyagawa et al., 2024), and neurotransmitter uptake by astrocytes (Izrael et al., 2018). Drug response was often integrated in other assays like hormone secretion or electrophysiological analysis to assess cell activation or pathway specificity by applying stimulating compounds and challenging the cells with inhibitors (Iworima et al., 2024; Izrael et al., 2018; Kobayashi et al., 2024; Miyagawa et al., 2024; Naderi et al., 2023; Shin et al., 2022; Yu et al., 2023).

Potency evaluation by marker expression was assessed through bulk and single-cell RNA sequencing (Huang et al., 2023; Neumayer et al., 2024; Wu et al., 2020; Yu et al., 2023), IF imaging (Neumayer et al., 2024; Soma et al., 2024; Surendran et al., 2022), and flow cytometry (Neumayer et al., 2024; Sugimoto et al., 2022a). These methods were rarely used as stand-alone tests and required additional steps to validate their suitability by demonstrating a correlation with true biological activity (Neumayer et al., 2024). In addition, PSC-derived cell potency was often compared to primary cells or cell lines of the same lineage to demonstrate functional equivalence.

In conclusion, the potency of PSC-derived products can be evaluated in a multitude of ways using product-specific parameters, with hormone and cytokine production being the most applied approach for measuring biological activity. Suitable assays need to be determined by the product developers in consultation with the regulatory authorities. Surrogate methods correlating with biological activity can be validated when justified—usually when measuring direct potency as a QC release test is not feasible. Careful case-by-case consideration is required for designing QC strategies and evaluation of the desired quality product profile, which is highly related to the clinical indication for which the product is developed.

### Stability

Cell product attributes related to identity, impurities, content, genomic integrity, and potency have been tested in stability studies to evaluate the persistence of these CQAs. This included the impact of prolonged culture; in-use stability during room temperature preservation, storage in the formulation solution, and transportation to the clinical site; long-term cryopreservation; and delivery through medical devices, where applicable (Figure S4; Table S9).

The dominant CQA assessed across all categories of the stability studies was cell viability (Gantier et al., 2024; Kirkeby et al., 2023; Kobayashi et al., 2024; Naderi et al., 2023; Watari et al., 2023; Wu et al., 2020; Yu et al., 2023), succeeded by marker expression evaluating identity and purity of the product using flow cytometry, qPCR, and IF imaging of on- and off-target cells and absence of pluripotency markers (Gantier et al., 2024; Kirkeby et al., 2023; Kobayashi et al., 2024; Naderi et al., 2023; Sugimoto et al., 2022b; Watari et al., 2023; Yu et al., 2023). Cell morphology was the third most common attribute often assessed by bright-field (Gantier et al., 2024) or IF imaging (Watari et al., 2023; Yu et al., 2023) of replated cells or multilayered structures (Sugimoto et al., 2022a). Specifications for cell count, general appearance of the suspension, and pH were defined in a shipping test critical for the release of a platelet product after transportation to the clinical care facility (Sugimoto et al., 2022a). Simulation of storing the cell preparation in a formulation buffer at 4°C or on ice prior to the transplantation or during transportation was assessed by inspecting cell viability for up to 4-72 h (Kobayashi et al., 2024; Naderi et al., 2023; Wu et al., 2020; Yu et al., 2023). Genomic stability was assessed by karyotyping (Naderi et al., 2023) or by an SNP array (Kirkeby et al., 2023), which was classified as an FIO test for clinical release (Figure 2A) and compared CNV and CN-LOH events between directly thawed cells and after 14 days of additional culture. Prolonged *in vitro* culture of 4–12 weeks was also applied for cardiac spheroids to monitor their viability, nourishment of the core structure, and cellular purity over extended periods (Kobayashi et al., 2024) (Figure S4). Furthermore, in-use and cryopreservation stability was evaluated by assessing potency through marker expression (Sugimoto et al., 2022a), cytokine secretion (Surendran et al., 2021), and replating ability to demonstrate functional adhesive properties (Gantier et al., 2024). The longest time-points for stability testing after cryopreservation were reported at 6 months (Naderi et al., 2023; Yu et al., 2023) and at 36 months (Gantier et al., 2024; Kirkeby et al., 2023) with a planned extension to a total of 60 months (Kirkeby et al., 2023). The impact of a delivery device was monitored immediately after extruding the cells (Kirkeby et al., 2023; Naderi et al., 2023; Yu et al., 2023) and up to 11 months post-transplantation in a porcine *in vivo* study with a single animal to observe the survival and maturation of the grafted cells (Kirkeby et al., 2023).

In conclusion, stability studies mostly focused on detecting changes in cell viability and product composition, evaluated by marker expression. The handling conditions reflected the period after manufacturing and prior to patient transplantation, which included storage, transportation, final formulation, and administration through a delivery device. These items represent critical steps in product handling that could affect the product quality.

### Non-clinical *in vivo* studies

Non-clinical animal studies remain an essential part of safety and efficacy evaluation during the development of medicinal products, including PSC-derived therapies. A number of parameters were extracted from the included reports regarding the design of the *in vivo* studies to identify the current practices in biodistribution, tumorigenicity, toxicity, and efficacy assessment (Table S10-S13).

#### Animal models

Key aspects of animal models, such as species, immunosuppression and disease state, number of animals used, sex, and follow-up duration, were extracted from the included reports containing *in vivo* testing (Bershteyn et al., 2023; Cichocki et al., 2022; Corsini and Mitola, 2023; Gantier et al., 2024; Huang et al., 2023; Izrael et al., 2018; Kirkeby et al., 2023; Kobayashi et al., 2024; Luciani et al., 2022; Manley et al., 2017; Miyagawa et al., 2022, Miyagawa et al., 2024; Naderi et al., 2023; Neumayer et al., 2024; Oh et al., 2022; Park et al., 2024b; Rong et al., 2024; Shigyo et al., 2023; Shin et al., 2022; Soma et al., 2024; Sugimoto et al., 2022a, Sugimoto et al., 2022b; Surendran et al., 2021; Wang et al., 2024; Watari et al., 2023; Wu et al., 2020, Wu et al., 2024; Yu et al., 2023) (Figures 4, S5, and S6).

**Figure 4:**
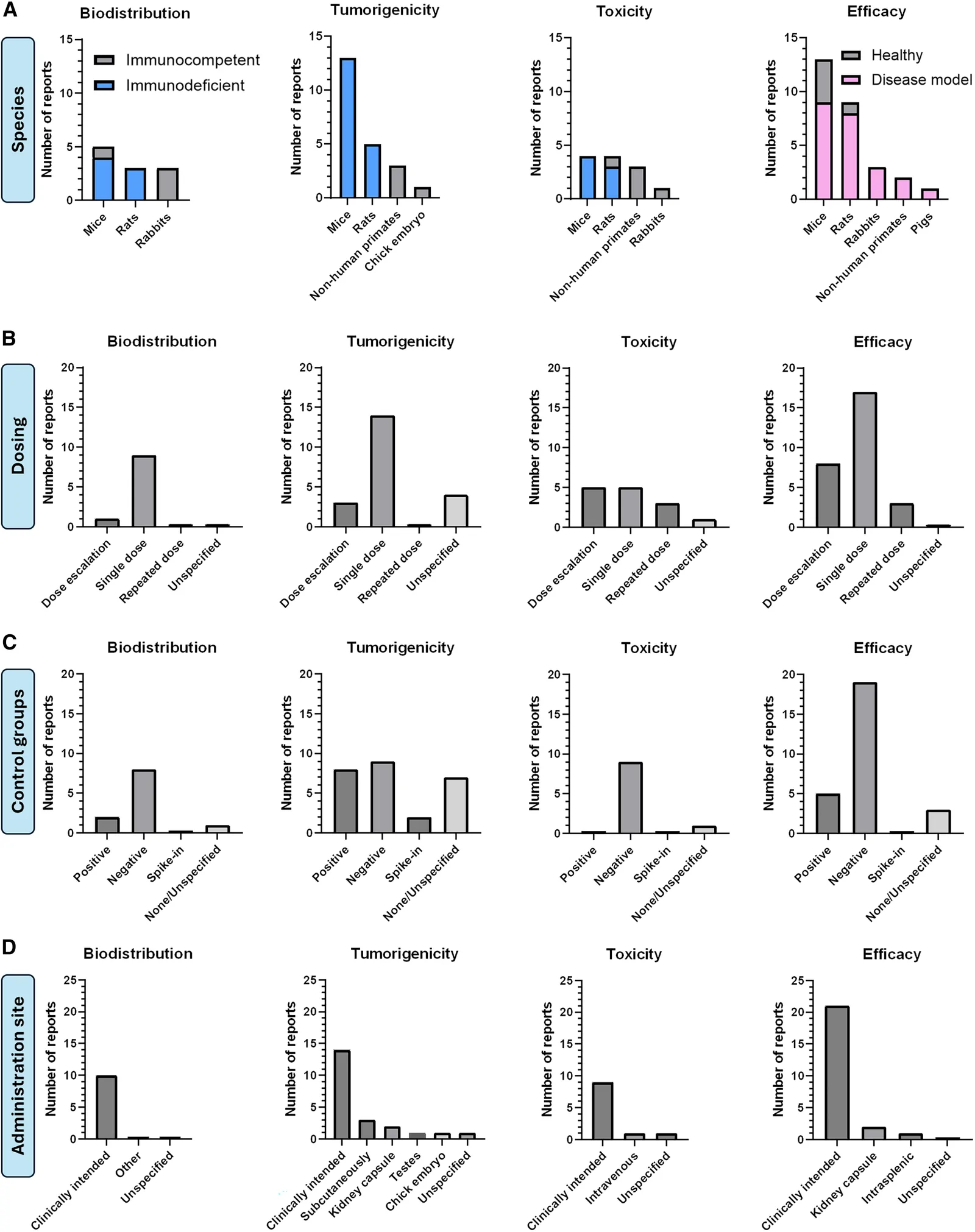
Animal species and product dosing in non-clinical studies of PSC-derived therapy products (A) Animal species and employment of immunosuppression or disease models in non-clinical safety and efficacy assessments described in the included reports. (B) Number of different product doses tested for PSC-derived products in animal studies. (C) Inclusion of control groups in the non-clinical studies. (D) Sites of administration through clinically intended or other routes utilized in the analyzed *in vivo* studies. See also Figures S5 and S6; Tables S10–S13.

Mice were the predominant species used for all studies, closely followed by rats (Figure 4A). For most safety and efficacy studies in large animal models, non-human primates and rabbits were the model of choice, while pigs were the preferred model to test the efficacy of specific products like cardiomyocyte patch (Miyagawa et al., 2024). To assess teratoma formation caused by ESCs, a chick embryo model was proposed, offering a growth-stimulating highly-vascularized environment and allowing to monitor the teratoma formation through a transparent window (Corsini and Mitola, 2023).

Most of the safety studies were conducted in immunocompromised murine strains, while large animal models were genetically unaltered (Figure 4A). In the majority of the reviewed reports, drug-based immunosuppression was not used or remained unreported (Figure S5A), which could be attributed to the preferred use of the genetically induced immunodeficient mice and rats. However, when immunosuppressant use was reported, it was administered to both immunocompetent and immunodeficient animals. Immunosuppressive drugs in animal models can not only be administered to prevent rejection of a xenograft but also to mimic clinical conditions where immunosuppressive treatment is applied as an adjuvant. Meanwhile, efficacy was primarily evaluated in disease models induced genetically, mechanically, or via drug or toxin treatment (Figure 4A). In some cases, studies were performed in healthy animals in addition to diseased mice (Bershteyn et al., 2023; Wang et al., 2024) and non-human primates (Kobayashi et al., 2024).

The number of animals varied per study, not only between but also within the groups of the same species (Figures S6A and S6B). Based on the available reported data, the total number of animals, including all dose groups, controls, and spares (Figure S6A), as well as the lowest animal number used to test a single dose of a product (Figure S6B) generally ranged from a few up to a cohort of hundreds for mice and rats. In studies with other species, considerably lower animal numbers were reported, reaching up to a few dozen for a minimum cohort used to test a single dose of a product (Figure S6B) and for complete studies (Figure S6A).

Where sex was specified, a study set-up using a single sex or a mixed population was reported at a similar rate for biodistribution, tumorigenicity, and efficacy assessment (Figure S5B). For toxicity studies, a mixed population was more often chosen. One report mentioned a limited possibility to include both male and female pigs (Miyagawa et al., 2024), while another report justified their choice for female rats in tumorigenicity evaluation of retinal grafts as necessary to limit aggressive behavior among co-housed animals (Watari et al., 2023).

The longest follow-up duration was generally observed for mice and rats, varying from 7 days studying biodistribution of platelets (Sugimoto et al., 2022a) and efficacy of MSCs (Shin et al., 2022) to 1.5 years monitoring efficacy of interneurons (Bershteyn et al., 2023) and tumorigenicity of retinal sheets (Watari et al., 2023) (Figure S6C). Non-human primates were followed for a maximum of 4 weeks in an efficacy study of islets (Wu et al., 2024) to 36 weeks in a long-term toxicity study of repeatedly dosed immunity-and-matrix-regulatory cells (IMRCs) (Huang et al., 2023). Rabbits were used to follow the biodistribution of platelets for up to 24 h (Sugimoto et al., 2022a) and toxicity and efficacy of IMRCs in meniscus regeneration for 8 weeks (Huang et al., 2023), while cardiac regeneration in pigs was observed for 12 weeks (Miyagawa et al., 2024). Teratoma detection in the proposed chick embryo model could be achieved in a rapid turnaround time of 10 days (Corsini and Mitola, 2023).

#### Dosing studies, controls, and site of administration

An essential part of a testing strategy for a new medicinal product is deciding on the dosing. Most of the *in vivo* studies assessed the effect of a single dose of cells (Figure 4B). The selected dose was often related to the estimated cell number required for efficacy or a maximal feasible dose limited by the volume constraints to increase the possibility of detecting toxic effects, tumorigenesis, or ectopic distribution. Dose-escalation of 2–5 different cell amounts was primarily utilized in finding a therapeutically effective or non-toxic dose. Furthermore, repeated dosing was used to assess toxicity and efficacy, with the most extensive example of IMRCs administered intravenously up to 22 times within 24 weeks in non-human primates (Huang et al., 2023; Wu et al., 2020).

In addition to the animal groups dosed with the cell product, negative control groups were often used for comparison (Figure 4C). Negative controls either remained untreated (Cichocki et al., 2022; Gantier et al., 2024; Naderi et al., 2023; Rong et al., 2024; Shin et al., 2022; Wang et al., 2024; Watari et al., 2023), were sham-operated (Miyagawa et al., 2024; Shin et al., 2022; Watari et al., 2023; Wu et al., 2024), or were dosed with the vehicle (saline, medium, or formulation buffer) (Bershteyn et al., 2023; Huang et al., 2023; Izrael et al., 2018; Kirkeby et al., 2023; Kobayashi et al., 2024; Manley et al., 2017; Naderi et al., 2023; Park et al., 2024b; Rong et al., 2024; Sugimoto et al., 2022a, Sugimoto et al., 2022b; Surendran et al., 2021; Wu et al., 2020; Yu et al., 2023); Matrigel for tumorigenicity studies (Soma et al., 2024; Wang et al., 2024); carrier cells to maintain cell density (Kirkeby et al., 2023); dispersed cells compared to spheroids (Kobayashi et al., 2024); gene-engineered cells containing the backbone construct (Cichocki et al., 2022); or intermediate cell product (Sugimoto et al., 2022a; Wu et al., 2024). Positive controls were mainly used in tumorigenicity studies by administering cancer cell lines (Oh et al., 2022; Park et al., 2024b; Sugimoto et al., 2022a), PSCs (Kirkeby et al., 2023; Neumayer et al., 2024; Soma et al., 2024; Wang et al., 2024; Wu et al., 2024; Yu et al., 2023), or unpurified product (Miyagawa et al., 2024). Spike-in controls with PSCs (Park et al., 2024b) or patient-derived induced carcinoma cells (Neumayer et al., 2024) mixed into the product or differentiated cells enabled titrating the number of cells able to initiate tumor growth. Controls consisting of intermediate cell product (Sugimoto et al., 2022a) or PSCs (Neumayer et al., 2024) in biodistribution studies were used to characterize the general cell dissemination or possible cancer metastases. Some efficacy studies compared the cell therapy product to approved chemical drugs (Cichocki et al., 2022; Shin et al., 2022; Wu et al., 2020), primary cells (Cichocki et al., 2022; Gantier et al., 2024; Neumayer et al., 2024; Wu et al., 2020), or equivalent PSC-derived research-grade cells (Shin et al., 2022).

Site of administration may be a key determinant for cell efficacy, tumor-stimulating environment, and routes of dissemination. Subcutaneous injection in a murine model has been described as a gold standard for studying teratoma formation (Corsini and Mitola, 2023) and was utilized for detecting possible tumor growth by transplanting corneal endothelial and epithelial cells (Soma et al., 2024; Yu et al., 2023) (Figure 4D). However, across all *in vivo* studies, cell products were predominantly administered to the clinically intended site, including the brain, spinal cord, heart, vein, eye, skin, joint, muscle sheath, and bladder. Sometimes, other sites (testes (Wu et al., 2020)) and model systems (chick embryos; Corsini and Mitola, 2023) were preferred, possibly for their hypoimmunogenic environment. Islet cells were transplanted under the kidney capsule in mice to evaluate their potential tumorigenicity and functionality (Wang et al., 2024; Wu et al., 2024). Toxicity of IMRCs intended for meniscus injury was tested by intravenous infusion (Huang et al., 2023), while intrasplenic injection of hepatic progenitor cells facilitated the delivery of cells to the liver due to the limited access to the portal vein in mice (Gantier et al., 2024).

#### GLP conditions

Good Laboratory Practice (GLP) is an established quality system for conducting non-clinical safety studies, ensuring appropriate planning, performance, monitoring, recording, archiving, and reporting (FDA, 2025; The European Parliament and the Council of the European Union, 2004). GLP conditions are usually applied for pivotal studies designed to obtain confirmatory data on product safety for submission to the regulatory authorities. Among the reported animal studies, the majority did not mention the application of GLP conditions (Figure S5C). Biodistribution and toxicity studies were most frequently performed under GLP conditions (4/10) (Izrael et al., 2018; Kirkeby et al., 2023; Manley et al., 2017; Park et al., 2024b), followed by tumorigenicity studies (6/20) (Izrael et al., 2018; Kirkeby et al., 2023; Manley et al., 2017; Oh et al., 2022; Park et al., 2024b; Shin et al., 2022). GLP conditions were rarely applied for efficacy studies (1/23). Most studies following GLP regulations were outsourced to third parties (Izrael et al., 2018; Kirkeby et al., 2023; Manley et al., 2017; Park et al., 2024b; Yu et al., 2023), while some were performed within the manufacturer’s own facilities (Oh et al., 2022; Shin et al., 2022) (Tables S10–S13). Conversely, some non-GLP studies were transferred to external facilities (Wu et al., 2020, Wu et al., 2024).

#### Use of *in vivo* studies for QC testing

Interestingly, while most non-clinical *in vivo* studies were performed during the development phase of the product, some *in vivo* tumorigenicity assays were included as QC release tests (Miyagawa et al., 2022; Wang et al., 2024). Allogeneic cryopreserved iPSC-derived cardiomyocytes intended for use in a clinical trial were first transplanted as a patch onto the hearts of 10 immunodeficient mice, and the product was released after no tumor formation was observed at the 16-week endpoint (Miyagawa et al., 2022). Another report specified a similar 4-month study with 50 immunodeficient mice as a QC release test of an autologous cryopreserved islet product transplanted under the kidney capsule, using Matrigel with ascitic fluid and the iPSC Master Cell Bank starting material as negative and positive controls, respectively (Wang et al., 2024). For cryopreserved allogeneic (Miyagawa et al., 2022; Wu et al., 2020) and autologous (Wang et al., 2024) products released as one batch, the possibility of long-term preservation contributed to the feasible implementation of a release test with a substantial lead time.

### Typical profile of non-clinical studies and quality testing

From the available information, it is evident that the outcomes on efficacy and tumorigenicity of PSC-derived cell therapy products were reported more frequently than biodistribution and toxicity (Figure 4A). The typical profile of non-clinical safety and efficacy studies delineated a cohort nearing a hundred immunosuppressed rodents with a balanced male/female ratio, comparing the effect of a single dose of the cell product to a negative control, administered via a clinically relevant route and followed up for a median of 5–10 months.

The detailed systematic data extraction of the quality testing strategies enabled us to compile a list of CQAs and applied methods for QC release testing of PSC-derived medicinal products for clinical application (Table S14). These QC tests ranged over a spectrum of universality and application frequency (Figure 5). Some techniques can be universally applied for multiple PSC-derived cell lineages and were widely implemented. The other end of the spectrum hosts tests that were relatively less frequently observed within the corresponding testing groups and analyzed product-specific properties, mostly dependent on cell function and mechanism of action. Meanwhile, methodologies clustering in the middle of the spectrum can be universal across PSC-derived medicinal products, however, they were reported at a lower frequency. These methods included both approved QC tests as well as proposed, emerging, and potentially informative methods ranked as pQC and FIO. In addition, we provide a product-specific overview of the performed QC release tests per product type and the available specifications or reported results, which could benefit therapy developers interested in comparing the methods and acceptance criteria applied to similar products (Table S15). This resulted in a comprehensive resource providing information on testing strategies that have been used to qualify cell and tissue-engineered products for clinical application, fit-for-purpose methods to evaluate relevant CQAs, and reported specifications allowing the comparison of acceptance criteria.

**Figure 5:**
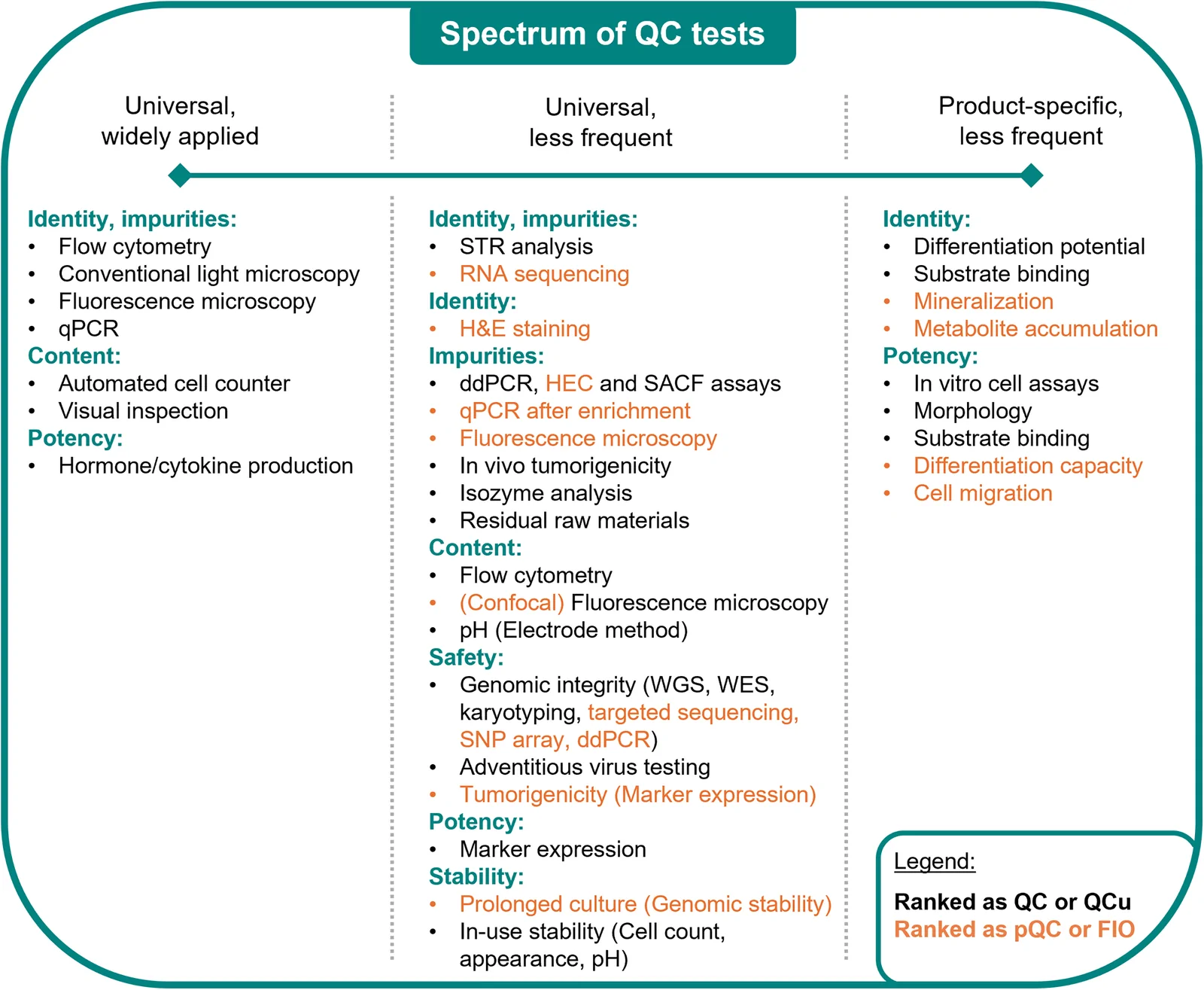
Spectrum of QC tests on the scale of universality and frequency of application Methods on the left side can be utilized universally for multiple PSC-derived cell lineages and were frequently reported for QC release testing. Methods on the right side focus on product-specific properties mostly related to cell functionality and were used less frequently compared with other techniques within the same groups of tests. Methods in the middle of the spectrum represent potentially widely applicable methodologies which were less frequently reported. Methods in black were ranked as QC or QCu, while methods in orange obtained pQC or FIO as the highest applied ranks.

## Discussion

In this systematic review, we provided a comprehensive overview of the quality and non-clinical testing strategies reported for various iPSC- and ESC-derived medicinal products in recent years. An inclusive literature search yielded reports on diverse PSC-derived products from different geographical regions, many of which have passed a regulatory checkpoint before starting clinical trials. We grouped a wide range of analyzed CQAs with regards to PSC-derived cell product identity, purity, content, safety, potency, and stability, constructing a traceable dataset for delving into product-specific cases of interest. Using a custom ranking system for quality tests, we ranked the methods revealing the trends in the most frequently assessed CQAs and preferred techniques for general characterization as well as for release testing. A summary of all techniques ranked as QC and QCu is intended to serve as a “QC menu card” listing recognized methods employed for assessing CQAs of different PSC-derived medicinal products during product release. Meanwhile, the product-specific overview with release test specifications is meant to provide a starting point for developers designing a QC testing panel using earlier experience as a blueprint.

This work was focused on terminally differentiated products derived from iPSCs and ESCs, which are the two main types of PSCs used to generate medicinal products (Kirkeby et al., 2025). In our analysis, we encountered a lower-than-expected testing frequency for some CQAs, such as viral safety and genomic integrity assessment. A possible explanation for missing seemingly critical analyses could be the use of interchangeable approaches, or manufacturing in-process controls and upstream QC tests performed on starting materials and intermediate products, such as donor screening and Master Cell Bank validation, falling beyond the scope of this review. It is also possible that the underrepresentation of certain testing modalities relates to the fact that some QC and non-clinical tests performed on the final formulation of the products may be undisclosed, reflecting publication bias, or may have been published as part of the pre-clinical development prior to the established cut-off for record retrieval. The need to include additional cross-referenced reports manually (Izrael et al., 2018; Manley et al., 2017; Surendran et al., 2021; Wu et al., 2020) demonstrated the fragmented reporting of test panels. Therefore, overall conclusions about the completeness of the testing strategies cannot be drawn from this dataset. Standardized and complete reporting of testing panels, applied methods, and acceptance criteria could improve the comparability of the studies and adaptation for future products, expediting product development, regulatory review process, and patient access to novel therapies worldwide.

The “QC menu” of recognized release tests can furthermore be extended with the techniques ranked as pQC tests, comprising state-of-the-art methods undergoing validation for this purpose or indicated as provisional QC release tests for developed products which were not yet applied in clinical trials at the time of data analysis. The field of PSC-derived therapies remains a dynamic domain in which novel assay developments and improvements are encouraged to accommodate the diversity of the final products and establish more rapid, sensitive, and cheaper procedures (O'Shea et al., 2020). The laborious *in vivo* and *in vitro* assays for adventitious virus detection, which can be limited by low detection sensitivity, variable cell line susceptibility, and long testing time, have the prospect to be replaced by the NGS (ICH, 2023). Possibly due to the relatively recent regulatory acceptance of the revised ICH Q5A(R2) guideline in 2023 (ICH, 2023), NGS application for adventitious virus testing of the final products was not observed in the included reports. Standardization of testing panels and defining minimum technical requirements such as the sequencing depth appropriate for adventitious virus or genomic integrity evaluation could contribute to a wider implementation of NGS in QC testing. During our systematic search, several novel methods for QC testing of PSC-derived cells have been retrieved, which were excluded from the main analysis due to early development stages and limited fit-for-purpose evidence. A few promising methods which addressed a clear CQA-specific challenge and were proposed for QC testing are Raman imaging of metabolites for assessing cellular purity (Li et al., 2023); multifunctional microelectrode array detecting exocytosis for cell functionality (He et al., 2022); atomic force microscopy evaluating nanomechanical properties for functional evaluation (Herardot et al., 2024); and artificial intelligence (AI) algorithms of cell morphology supporting identity assessment (Lien et al., 2023; Marzec-Schmidt et al., 2023). Other up-and-coming techniques currently transforming the field are multi- and spatial omics coupled with powerful AI-assisted analysis algorithms (Gong et al., 2025; Yang et al., 2025), which could be especially valuable for assessing engineered tissue constructs at a cellular level while preserving the architecture which defines product’s quality and functionality. Moreover, all these established and novel techniques can comprise not only the final product testing strategies, but could also support in-process control, comparability studies, and long-term evaluation throughout the product life cycle. Robust comparability assessments may be particularly important for PSC-derived medicinal products, as manufacturing processes often evolve due to process modifications, production scaling, or manufacturing site transfer, requiring demonstration that CQAs remain consistent throughout development, upscaling, and commercialization.

In conclusion, this review creates a consolidated resource of recent QC and non-clinical testing practices for PSC-derived products. Insights into state-of-the-art and established fit-for-purpose testing methods, revealed by the custom ranking system, can benefit product developers and regulatory authorities by providing a toolkit for devising future QC strategies. Taken together, this review highlights both universal methods and product-specific solutions, identifies gaps, and points to the need for harmonization, transparent reporting, and continued innovation to ensure safe and effective clinical translation of PSC-derived therapies.

## Acknowledgments

This work was supported by the Novo Nordisk Foundation Center for Stem Cell Medicine reNEW (NNF21CC0073729).

## Author contributions

A.G., conceptualization, methodology, investigation, validation, formal analysis, data curation, visualization, writing – original draft, and writing – review and editing; M.T., conceptualization, methodology, investigation, validation, formal analysis, supervision, and writing – review and editing; I.v.A.-W., investigation, validation, and writing – review and editing; F.L., investigation and validation; P.G., methodology; B.R., supervision and writing – review and editing; E.d.K., supervision, funding acquisition, and writing – review and editing; F.C., supervision, funding acquisition, and writing – review and editing; P.M., conceptualization, supervision, project administration, funding acquisition, and writing – review and editing.

## Declaration of interests

The authors declare no competing interests.

## Supplemental information

Document S1. Figures S1–S7, Tables S1–S14 and S16, Supplemental methods, and Supplemental references

Table S15. Tests applied in QC testing of PSC-derived cell therapy products grouped by cell type and specifications provided in the included reports

Document S2. Article plus supplemental information
