# Supplementary material for: Quality control and non-clinical testing of iPSC- and ESC-derived medicinal products: A systematic review
Source: Stem Cell Reports. 2026 Sep 24;21(10):103084. doi: 10.1016/j.stemcr.2026.103084 (PMC13681315; doi:10.1016/j.stemcr.2026.103084)
Supplement: Supplementary file 1 [file mmc1.pdf]

Stem Cell Reports, Volume *21*

Supplemental Information

Quality control and non-clinical testing of iPSC- and ESC-derived medicinal products: A systematic review

Akvilė Gasiūnaitė, Mara Tihaya, Inge van Amerongen-Westra, Fong Lin, Pema Gurung, Bahareh Rajaei, Eelco de Koning, Françoise Carlotti, and Pauline Meij

Supplemental figures

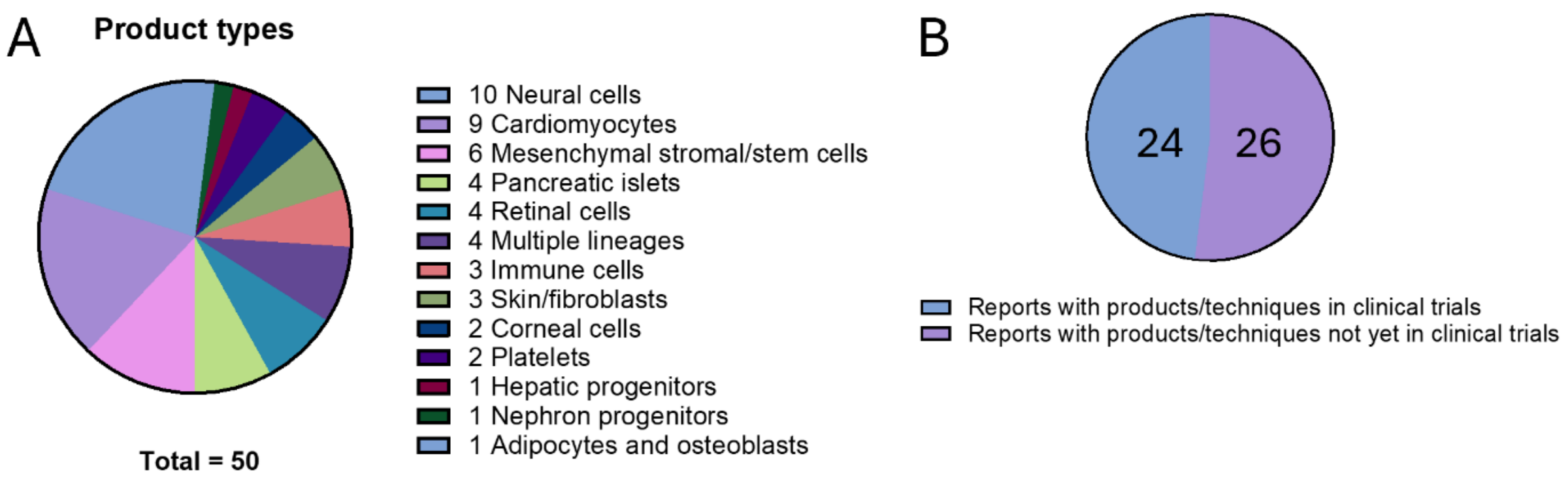

Figure S1. An overview of the included reports. A) Number of reports on PSC-derived products grouped by the end-differentiated cell types. See also Table S1. B) Clinical implementation of cell-based products and techniques in the included reports.

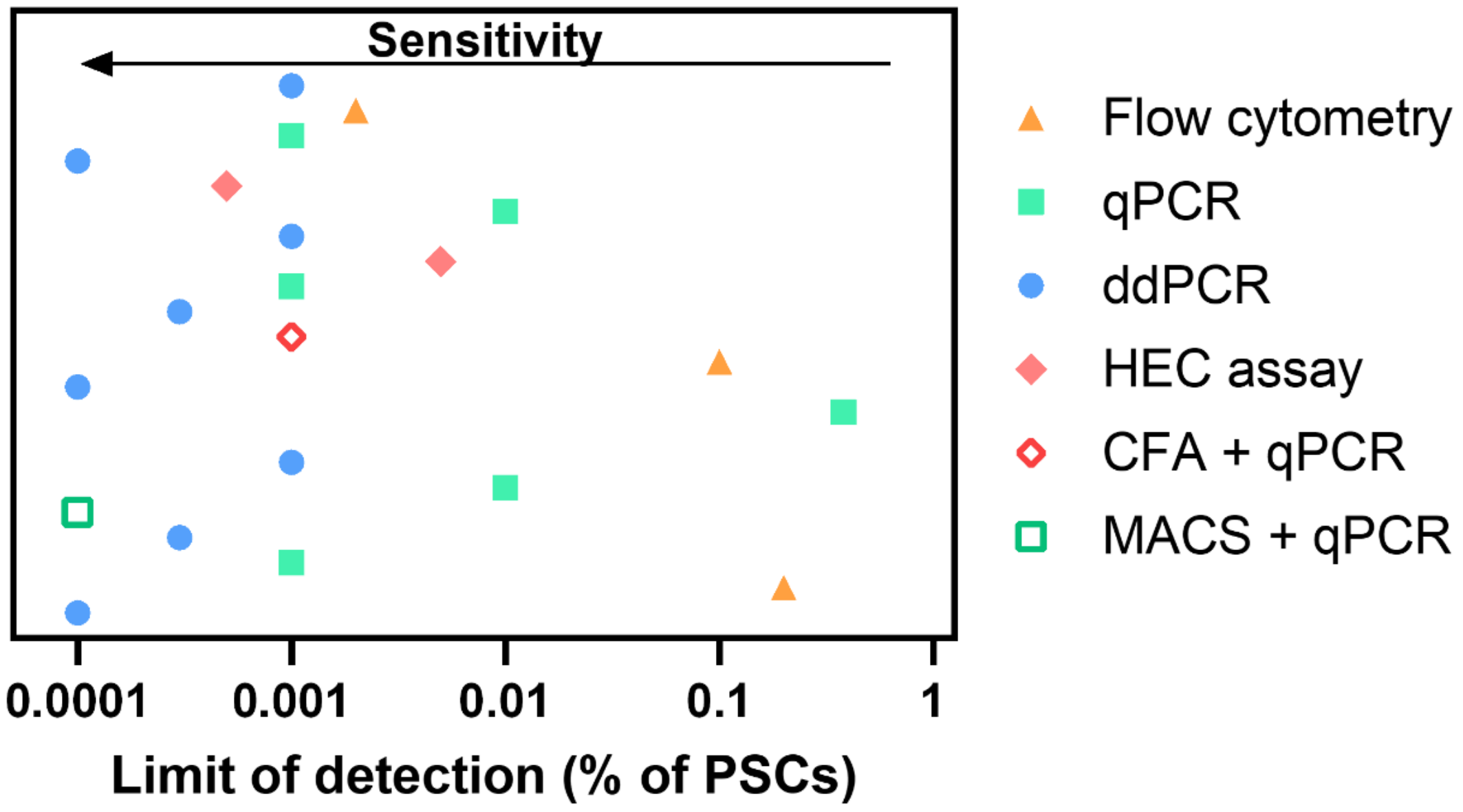

Figure S2. Limits of detection (LOD) reported for different methods employed in residual PSC detection, related to Figure 1B. The sensitivity to detect residual PSCs increases with decreasing LOD of the assay. Flow cytometry: N = 3 reports; qPCR: N = 6; ddPCR: N = 8; HEC assay: N = 2; CFA + qPCR: N = 1, MACS + qPCR: N = 1. CFA: colony formation assay, ddPCR: droplet digital PCR, HEC: highly efficient culture, MACS: magnetic-activated cell sorting, qPCR: quantitative PCR.

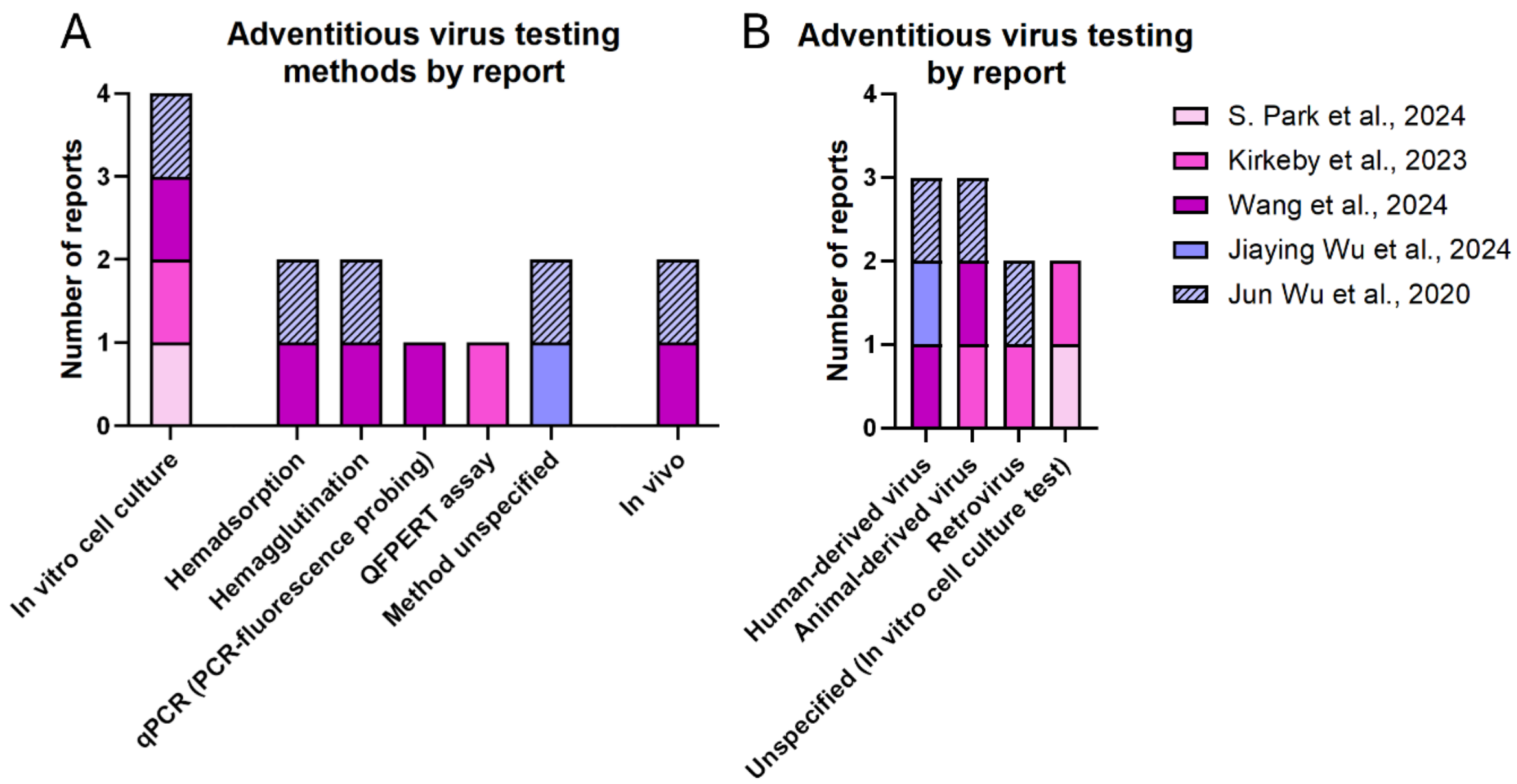

Figure S3. Reports that tested adventitious virus presence in the PSC-derived end-products, related to Figure 2. A) Graphical representation of different methods used for adventitious virus detection in each report. See also Figure 2C. B) Virus types and species targeted for detection. See also Figure 2D. qPCR: quantitative PCR, QFPERT: quantitative fluorescent product-enhanced reverse transcriptase.

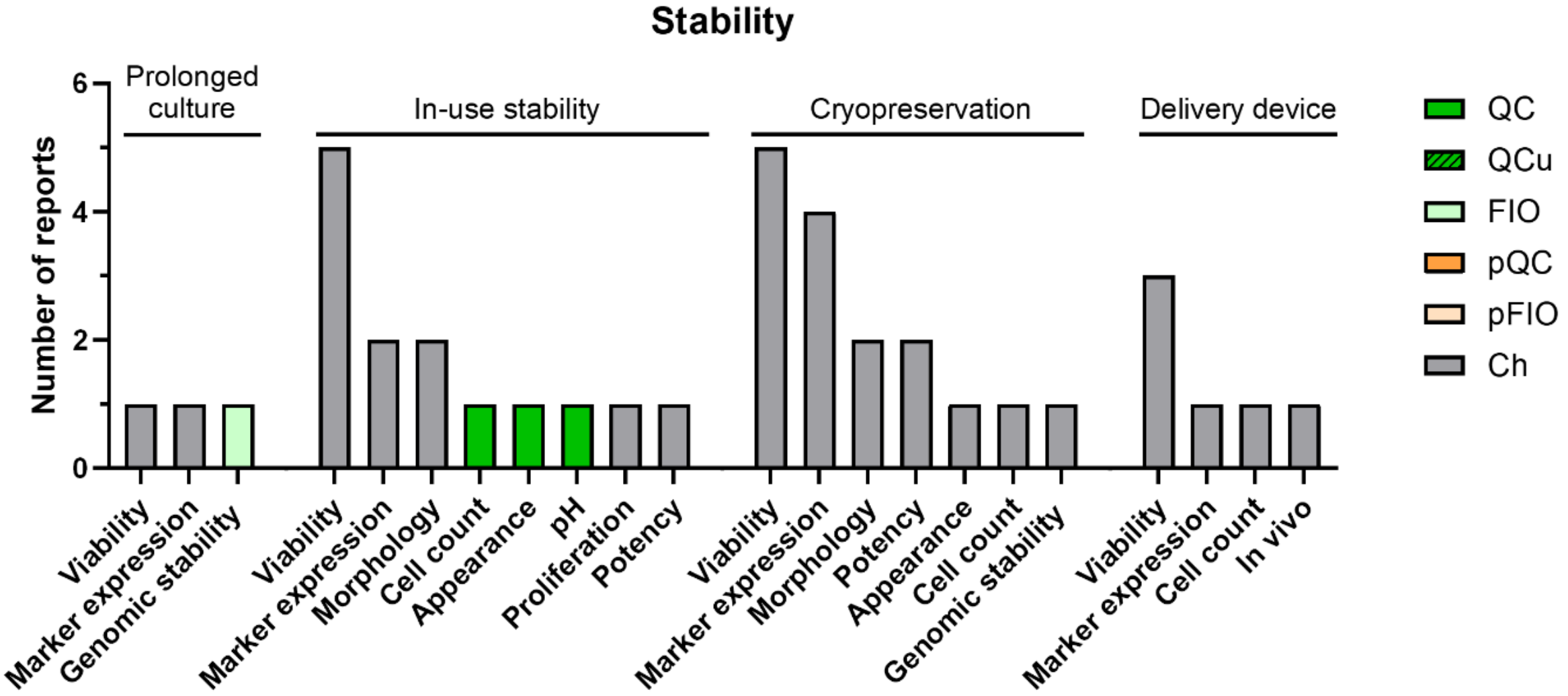

Figure S4. Stability testing of PSC-derived therapy products, related to Table S9. Different CQAs that were assessed to evaluate the effects of post-manufacturing product handling procedures, such as prolonged culture, in-use stability (during room temperature preservation, storage in the formulation solution, and transportation), cryopreservation, and delivery through an injection device.

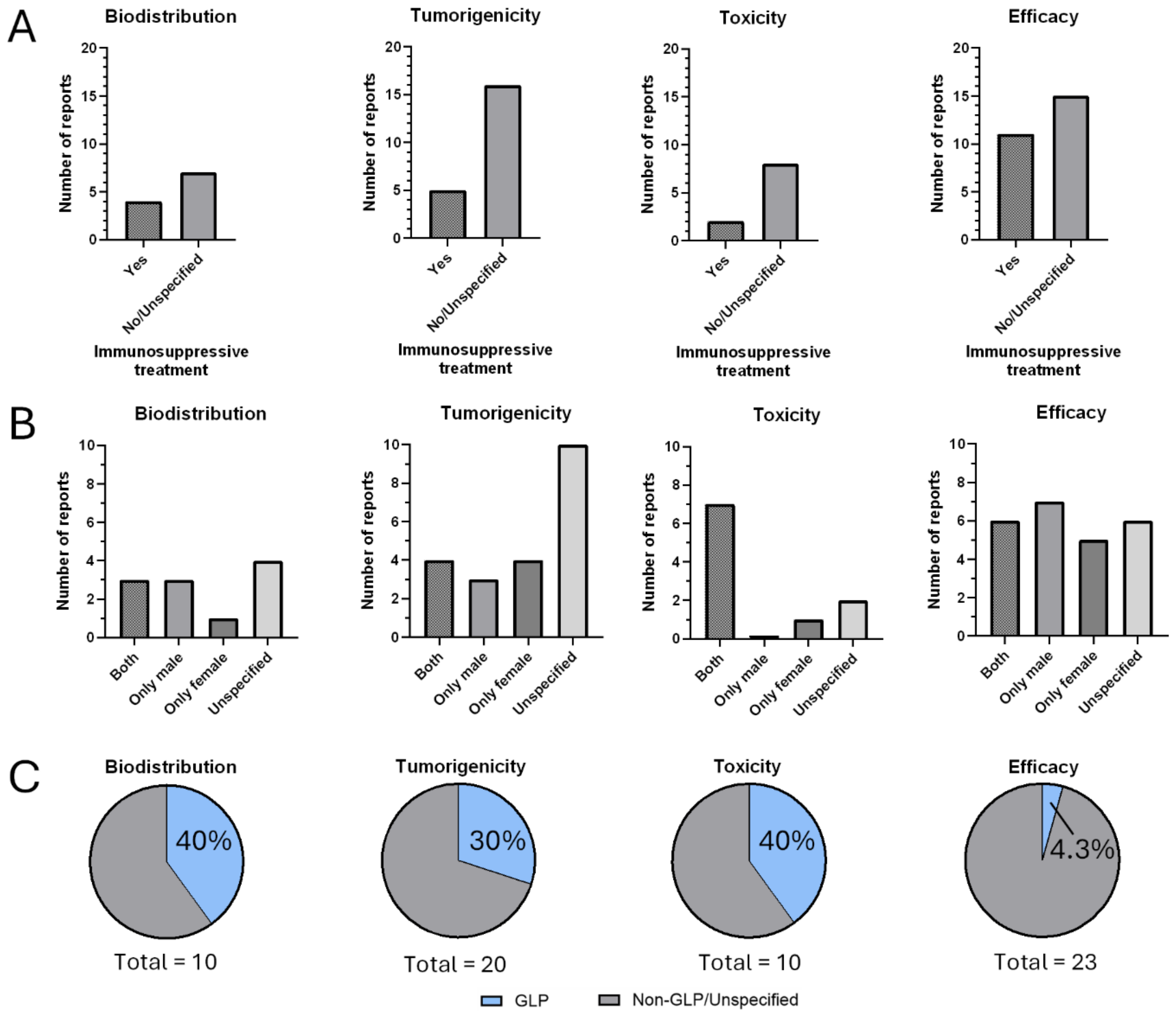

Figure S5. Animal model characteristics and study conditions in non-clinical assessment of PSC-derived therapy products, related to Figure 4 and Tables S10-S13. A) Use of the immunosuppressive drugs in animal studies. B) Use of male and female animals for PSC-derived product testing. C) Percentage of reports which applied GLP conditions in the non-clinical studies.

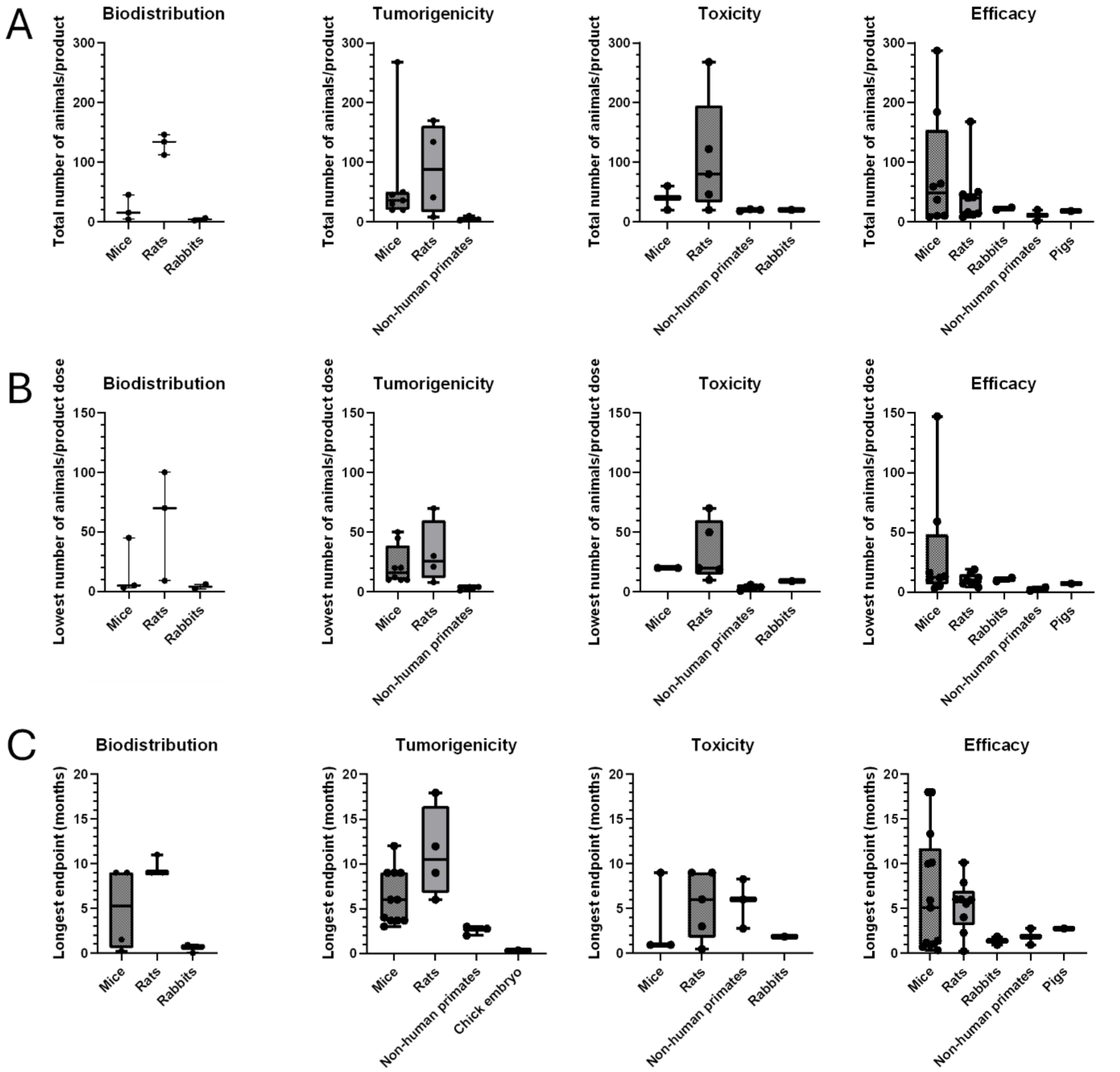

Figure S6. Animal numbers and follow-up duration in non-clinical assessment of PSC-derived therapy products, related to Figure 4 and Tables S10-S13. A) Total number of animals, including all dose groups, controls, and spares, used to test a single PSC-derived product described in the included reports. B) Lowest indicated number of animals used to test a single dose of the product. C) Longest endpoint of the follow-up duration in non-clinical studies per animal species. Data are represented as box plots with median line and whiskers showing minimum and maximum values. Individual dots represent the summed animal number per specific product.

Supplemental tables

Table S1. PSC-derived product types and their intended indications in the included reports, related to Figure S1.

Product type	Product	Disease	Reference numbers
Neural cells	Neural stem cells	Early-onset neurodegenerative or demyelinating CNS diseases	[1]
	Neural precursor cells	Spinal neurodegenerative disorders (spinal ischemic/traumatic injury, ALS, or multiple sclerosis)	[2]
	Dopaminergic progenitors	Parkinson’s disease	[3-5]
	Oligodendrocyte progenitor cells	Cervical spinal cord injury	[6, 7]
	Interneurons	Epilepsy	[8]
	Astrocytes	Amyotrophic lateral sclerosis	[9, 10]
Cardiomyocytes	Single cells	Heart failure	[11-15]
	Aggregates		[13, 16, 17]
	Patches		[18, 19]
MSCs	MSCs	Diabetic retinopathy, Interstitial cystitis	[20-23]
	IMRCs	Meniscus injury, Lung injury and fibrosis	[24, 25]
Retinal cells	Photoreceptors (retinal sheets)	Retinitis pigmentosa	[26, 27]
	RPE cells (suspension)	Age-related macular degeneration	[28, 29]
Immune cells	Macrophages	Inflammatory disorders and cancer	[30]
	NK cells	Multiple myeloma	[31, 32]

Skin grafts	Fibroblasts	-	[33]
	Keratinocytes and fibroblasts	Chronic wounds	[34]
	Keratinocytes, fibroblasts, and melanocytes	Dystrophic Epidermolysis Bullosa	[35]
Corneal cells	Corneal endothelial cells	Corneal endothelial dysfunction	[36]
	Corneal epithelial cell sheets	Limbal stem-cell deficiency	[37]
Multiple lineages	Including MSCs, RPEs, neural progenitor, hepatic, and endothelial cells	-	[38-41]
Other	Islets	Type 1 and 2 Diabetes	[42-45]
	Platelets	Aplastic anemia	[46, 47]
	Hepatic progenitors	Acute liver failure	[48]
	Nephron progenitor cells	Acute kidney injury	[49]
	Adipocytes and osteoblasts	-	[50]

ALS: amyotrophic lateral sclerosis, CNS: central nervous system, IMRCs: immunity-and-matrix-regulatory cells, MSCs: mesenchymal stromal/stem cells, NK cells: natural killer cells, RPE: retinal pigment epithelium.

Table S2. Identity tests of PSC-derived therapy products and their ranking, related to Figure 1A.

CQA	Method	Ranks and Reference numbers					
		QC	QCu	FIO	pQC	pFIO	Ch
Marker expression	Flow cytometry	[3, 5, 9, 18, 37, 44, 45, 47]	[6, 22, 24, 25]		[16, 21, 34, 36]	[28]	[2, 8, 17, 20, 30, 32, 35, 42, 48]
	IF imaging	[37]	[6]	[5]	[4, 34, 36]	[28]	[1-3, 8, 10, 17, 18, 26, 42, 44, 48]
	qPCR		[6, 27]	[5]	[4, 21]	[13]	[1, 3, 18, 28, 42, 45, 48]
	RNA-sequencing (single cell, bulk)				[30]		[4, 8, 18, 25, 29, 35, 36, 44, 45]
	HLA/HPA/ABO expression		[22, 24, 25]		[21]		[20, 46, 48]
	PCR	[3]					[26]
	MR-MAIPA assay						[46]
Morphology	Microscopic evaluation (bright-field/phase contrast)	[44, 45]	[22, 25]		[4, 34, 36]	[16, 21, 50]	[3, 17, 20, 26, 28, 30, 37, 42]
	H&E/IHC staining				[34]		[18, 26, 37]
	Transmission electron microscopy						[18, 47]
	Particle size/hematology analyzer						[47]
Metabolic	Glucose utilization and lactate accumulation				[42]		
	C-peptide accumulation in the medium				[42]		

Potency-related	Differentiation potential		[22, 24]				[20, 25]
	Dithizone (DTZ) staining	[44]					[42]
	Patch clamp						[16, 17]
	Annexin V binding	[47]					
	Mineralization					[50]	
	Lipid accumulation					[50]	
	Neurotransmitter secretion						[8]
	Migration potential						[8]
Genotype	STR analysis	[5, 44]	[25]				

HLA: human leukocyte antigen, HPA: human platelet antigen, H&E: hematoxylin and eosin, IF: immunofluorescence, IHC: immunohistochemistry, MR-MAIPA: modified rapid monoclonal antibody-specific immobilization of platelet antigens, STR: short tandem repeat.

Table S3. Impurity tests of PSC-derived therapy products and their ranking, related to Figure 1B.

CQA	Method	Ranks and Reference numbers					
		QC	QCu	FIO	pQC	pFIO	Ch
Off-target cells	Flow cytometry	[3, 5, 44, 45, 47]	[6, 22, 24, 25]		[21]	[16]	[8, 18, 20, 29, 32]
	qPCR	[26]	[6]	[5]	[4, 21]	[28]	[18, 42]
	RNA-sequencing (single cell, bulk)				[30]		[4, 8, 18, 25, 29, 35, 45]
	IF imaging		[6]		[4]		[3, 8, 17, 29, 42]
	PCR						[26]
	Morphology evaluation (bright-field microscopy)						[26]
	Morphology evaluation (automated hematology analyzer)						[46]
Residual PSCs	qPCR	[3]	[27]		[21, 36, 41]	[13, 14, 28]	[1, 18, 33, 35, 48]
	Flow cytometry	[5]	[9, 22, 25]		[8, 16]	[28]	[2, 48]
	IF imaging		[25]	[5]			[1, 3, 4, 18, 29, 36, 48]
	ddPCR	[37, 44]			[15, 31, 33, 39, 43, 49]		
	In vivo (mice, rats)		[25]				[3, 36]
	RNA-sequencing (single cell, bulk)						[8, 25, 29]
	Highly Efficient Culture (HEC) Assay				[12, 23]		
	PCR		[25]				
	PCR array						[18]
	RT-LAMP						[33]
	MACS + qRT-PCR				[49]		

	Colony formation assay (CFA) + qPCR (supernatant miRNA)					[13]	
	Method unspecified						[19]
Proliferating cells	Ki67 IF imaging				[36]		[3, 8, 10, 48]
	Ki67 Flow cytometry						[29]
	EdU IF imaging						[8]
	Population doubling time/level						[3]
Transformed cells	Soft agar colony formation (SACF) assay	[44]					[18]
	Cell growth assay/ Serial passage culture test						[18, 37]
Residual raw materials	Bovine serum albumin	[44]	[25, 47]				
	Growth factors	[44]					
	Small molecules		[47]				
	Cytokines		[47]				
Interspecies contamination	STR analysis	[44]					
	Isozyme analysis		[25]				

ddPCR: droplet digital PCR, EdU: 5-ethynyl-2´-deoxyuridine, IF: immunofluorescence, MACS: magnetic-activated cell sorting, RT-LAMP: reverse transcription loop-mediated isothermal amplification, STR: short tandem repeat.

Table S4. Methods for detecting residual PSCs in PSC-derived therapy products and their limit of detection, related to Figure 1B.

Method	Ref. no.	Rank	Material	Markers	Lowest reported LOD
Flow cytometry	[16]	pQC	Cells	OCT4	- (reported result 0.163%)
	[48]	Ch	Cells	OCT4	0.2%
	[2]	Ch	Cells	NANOG	-
	[5]	QC	Cells	OCT4/NANOG	≤0.1% (release specification)
	[9]	QCu	Cells	SSEA-4, EPCAM	-
	[28]	pFIO	Cells	OCT4	- (reported result 0.51%)
	[22]	QCu	Cells	OCT4, TRA-1-60, TRA-1-81	- (reported results 0.26%, 0.05%, 0.00%)
	[25]	QCu	Cells	TRA-1-60, SSEA-4, TRA-1-81	- (reported result <0.1%)
	[8]	pQC	Cells	TRA-1-60/OCT4	0.002%
qPCR	[3]	QC	Cells	*NANOG, TDGF1, REX1*	-
	[18]	Ch	Cells	*POU5F1, SOX2, NANOG, LIN28A*	-
	[48]	Ch	Cells	*OCT4, NANOG*	-
	[36]	pQC	Cells	*OCT4*	0.001%
	[13]	pFIO	Supernatant	miR-302b-3p	0.01%
	[35]	Ch	Cells	*LIN28A, NANOG, OCT4, SOX2*	0.38% (based on *LIN28A*)
	[14]	pFIO	Cells	*LIN28A, IDO1, CNMD*	0.001%
	[27]	QCu	Cells	*OCT4*	-
	[21]	pQC	Cells	*NANOG, OCT4*	-
	[28]	pFIO	Cells	*OCT4*	-
	[41]	pQC	Supernatant	miR-302b	0.001%
	[1]	Ch	Cells	*OCT4, NANOG*	-
	[33]	Ch	Cells	*LIN28A*	0.01%

ddPCR	[43]	pQC	Cells	*LNCPRESS2, LINC00678*	0.0001%
	[49]	pQC	Cells	*MIR302CHG*	0.001%
	[39]	pQC	Cells	*ESRG, ZSCAN10*	0.0001%
	[31]	pQC	Cells	miR-302a-5p, miR-302c-3p, miR-302d-5p	0.0003% (miR-302c-3p)
	[15]	pQC	Cells	*ESRG, LINC00678, LIN28A*	0.0003%
	[33]	pQC	Cells	*LIN28A*	0.001%
	[37]	QC	Cells	*LIN28A*	0.001%
	[44]	QC	Cells	*LNCPRESS2, LINC00678*	0.0001%
HEC assay	[12]	pQC	Cells	Alkaline phosphatase (ALP)	0.0005%
	[23]	pQC	Cells	Alkaline phosphatase (ALP)	0.005%
CFA + qPCR	[13]	pFIO	Supernatant	miR-302b-3p	0.001%
MACS + qPCR	[49]	pQC	Cells	*MIR302CHG*	0.0001%

CFA: colony formation assay, ddPCR: droplet digital PCR, HEC: highly efficient culture, LOD: limit of detection, MACS: magnetic-activated cell sorting.

Markers separated by a comma: evaluated individually; markers separated by a slash: double positive.

Table S5. Content tests of PSC-derived therapy products and their ranking, related to Figure 1C.

CQA	Method	Ranks and Reference numbers					
		QC	QCu	FIO	pQC	pFIO	Ch
Viability	Automated cell counter	[3, 5, 44]	[9]		[16]		[42, 48]
	Fluorescence microscopy	[44]				[16]	
	Flow cytometry	[45]					[8]
	Method unspecified	[18, 37]			[36]		[17]
Cell count	Automated cell counter	[3, 5]	[9]				[42, 44, 48]
	Flow cytometry	[47]					
	Confocal fluorescence microscopy					[16]	
	Method unspecified	[37]					[17]
General appearance	Visual inspection	[5, 37, 47]					[19]
pH	Electrode method	[47]					

Table S6. Safety tests of PSC-derived therapy products and their ranking, related to Figure 2.

CQA	Method	Ranks and Reference numbers					
		QC	QCu	FIO	pQC	pFIO	Ch
Genomic integrity	Whole genome sequencing	[44]		[37]	[35]		[18]
	Whole exome sequencing	[44]		[37]			[11, 18]
	Karyotyping		[22]		[34]		[2, 37]
	SNP array			[5, 37]			[8, 18]
	Targeted sequencing			[5]	[35]		[8, 11]
	ddPCR				[11]		[35]
	CGH array						[19]
	Microarray						[11]
	qPCR						[11]
Tumorigenicity	Soft agar colony formation (SACF) assay						[25, 47, 48]
	Marker expression					[18]	
	Proliferation test						[46]
Adventitious virus	In vitro cell culture	[3, 5, 44]	[25]				
	Hemadsorption	[44]	[25]				
	Hemagglutination	[44]	[25]				
	qPCR (PCR-fluorescence probing)	[44]					
	QFPERT assay	[5]					
	Method unspecified	[45]	[25]				
	In vivo	[44]	[25]				

CGH: comparative genomic hybridization, ddPCR: droplet digital PCR, QFPERT: quantitative fluorescent product-enhanced reverse transcriptase, SNP: single nucleotide polymorphism.

Table S7. Comparison of NGS methods used for genomic integrity testing, related to Figure 2A.

Method	Ref. no.	Rank	Depth coverage	Panel	Number of genes
WGS	[18]	Ch	56-83x	COSMIC, Cancer Gene Census, Shibata's list	
	[35]	pQC	~ 40x	-	
	[37]	FIO	71-76x	COSMIC Cancer Gene Census, Shibata's list	
	[44]	QC	~ 40x	COSMIC, Cancer Gene Census, CliniVar	
WES	[11]	Ch	~ 100x	COSMIC	
	[18]	Ch	84-114x	COSMIC, Cancer Gene Census, Shibata's list	
	[37]	FIO	83-148x	COSMIC Cancer Gene Census, Shibata's gene list	
	[44]	QC	~ 300x	COSMIC, Cancer Gene Census, CliniVar	
Targeted sequencing	[11]	Ch	~ 300x	ONCOAccuPanel	344 solid tumor-related genes
	[35]	pQC	-	STAMP oncopanel	130 genes, LOD allele frequency 5%
	[5]	FIO	-	Illumina NGS AmpliSeq Cancer HotSpot Panel	50 genes with 2800 COSMIC mutations; sensitivity of 5% mosaicism
	[8]	Ch	At least 100x	CleanPlex TP53 Panel (Paragon Genomics)	1 gene

COSMIC: Catalogue of Somatic Mutations in Cancer, STAMP: Stanford Actionable Mutation Panel for Solid Tumors, WES: whole exome sequencing, WGS: whole genome sequencing.

Table S8. Potency tests of PSC-derived therapy products and their ranking, related to Figure 3.

CQA	Ranks and Reference numbers					
	QC	QCu	FIO	pQC	pFIO	Ch
Hormone/cytokine production/secretion	[9, 44, 45]			[28, 42]		[3, 4, 18, 24, 25, 32, 48]
Drug response		[22]				[4, 10, 17, 18, 36, 42]
In vitro cell assays		[22, 24, 25]		[34]		[10, 32]
Morphological changes		[22]		[34]		[24, 25]
Differentiation capacity				[21, 34]		[2, 4]
Marker expression (RNA sequencing)						[24, 25, 35, 36]
Marker expression (IF imaging)	[37]					[28, 35]
Electrophysiological analysis						[4, 18, 36]
Marker expression (Flow cytometry)	[47]				[35]	
Substrate binding	[47]					
Cell migration				[21]		
Cell proliferation						[37]
Mechanical properties						[18]
Aggregation assay						[47]
Neurotransmitter uptake						[10]

IF: immunofluorescence.

Table S9. Stability tests of PSC-derived therapy products and their ranking, related to Figure S4.

Stability aspect	Method	Ranks and Reference numbers					
		QC	QCu	FIO	pQC	pFIO	Ch
Prolonged culture	Viability						[17]
	Marker expression						[17]
	Genomic stability			[5]			
In-use stability	Viability						[4, 17, 25, 26, 36]
	Marker expression						[26, 46]
	Morphology						[26, 47]
	Cell count	[47]					
	Appearance	[47]					
	pH	[47]					
	Proliferation						[26]
	Potency						[47]
Cryopreservation	Viability						[4, 5, 25, 36, 48]
	Marker expression						[4, 5, 36, 48]
	Morphology						[36, 48]
	Potency						[29, 48]
	Appearance						[5]
	Cell count						[5]
	Genomic stability						[4]
Delivery device	Viability						[4, 5, 36]
	Marker expression						[36]
	Cell count						[5]
	In vivo						[5]

Table S10. Non-clinical biodistribution studies of PSC-derived therapy products, related to Figure 4 and Figures S5-S6.

Study	Animal model		Reference numbers
Biodistribution	Species	Mice (immunodeficient)	[1, 10, 35, 47]
		Mice (WT)	[25]
		Rats (immunodeficient)	[3, 5, 7]
		Rabbits	[24, 36, 47]
	Male/Female ratio	Both	[3, 5, 24]
		Only male	[25, 36, 47]
		Only female	[7]
		Unspecified	[1, 10, 35, 47]
	Immunosuppressive treatment	Yes	[3, 7, 24, 47]
		No/Unspecified	[1, 5, 10, 25, 35, 36, 47]
	**Dosing**		**Reference numbers**
	Number of doses	Dose-escalation	[7]
		Single dose	[1, 3, 5, 10, 24, 25, 35, 36, 47]
		Repeated dose	-
		Unspecified	-
	Control groups	Positive	[35, 47]
		Negative	[3, 5, 7, 10, 24, 25, 36, 47]
		Spike-in	-
		None/Unspecified	[1]
	Transplantation site	Clinically intended	[1, 3, 5, 7, 10, 24, 25, 35, 36, 47]
		Other	-
	**Study conditions**		**Reference numbers**
	GLP study	Yes	[3, 5, 7, 10]
		No/Unspecified	[1, 24, 25, 35, 36, 47]
	Outsourced	Yes	[3, 5, 7, 10]
		No/Unspecified	[1, 24, 25, 35, 36, 47]

GLP: good laboratory practice, WT: wild-type.

Table S11. Non-clinical tumorigenicity studies of PSC-derived therapy products, related to Figure 4 and Figures S5-S6.

Study	Animal model		Reference numbers
Tumorigenicity	Species	Mice (immunodeficient)	[1, 10, 18, 19, 25, 35-38, 40, 44, 45, 48]
		Rats (immunodeficient)	[2, 3, 5, 7, 26]
		Non-human primates	[17, 44, 45]
		Chick embryo	[38]
	Male/Female ratio	Both	[3, 5, 17, 44]
		Only male	[25, 36, 45]
		Only female	[7, 18, 26, 38]
		Unspecified	[1, 2, 10, 19, 35, 37, 38, 40, 44, 48]
	Immunosuppressive treatment	Yes	[3, 7, 17, 44, 45]
		No/Unspecified	[1, 2, 5, 10, 18, 19, 25, 26, 35-38, 40, 44, 45, 48]
	**Dosing**		**Reference numbers**
	Number of doses	Dose-escalation	[7, 44, 45]
		Single dose	[1-3, 5, 10, 17-19, 26, 35-37, 44, 45]
		Repeated dose	-
		Unspecified	[25, 38, 40, 48]
	Control groups	Positive	[3, 5, 18, 36, 37, 40, 44, 45]
		Negative	[3, 5, 7, 10, 17, 26, 37, 44, 45]
		Spike-in	[3, 35]
		None/Unspecified	[1, 2, 19, 25, 38, 44, 48]
	Transplantation site	Clinically intended	[1-3, 5, 7, 10, 17-19, 26, 35, 40, 44, 45]
		Subcutaneous	[36-38]
		Kidney capsule	[44, 45]
		Testes	[25]
		Chick embryo chorioal lantoic membrane (CAM)	[38]
		Unspecified	[48]
	**Study conditions**		**Reference numbers**
	GLP study	Yes	[3, 5, 7, 10, 22, 40]
		No/Unspecified	[1, 2, 17-19, 25, 26, 35-38, 44, 45, 48]
	Outsourced	Yes	[3, 5, 7, 10]
		No/Unspecified	[1, 2, 17-19, 25, 26, 35-38, 40, 44, 45, 48]

GLP: good laboratory practice, WT: wild-type.

Table S12. Non-clinical toxicity studies of PSC-derived therapy products, related to Figure 4 and Figures S5-S6.

Study	Animal model		Reference numbers
Toxicity	Species	Mice (immunodeficient)	[10, 18, 22, 46]
		Rats (immunodeficient)	[3, 5, 7]
		Rats (WT)	[46]
		Non-human primates	[17, 24, 25]
		Rabbits	[24]
	Male/Female ratio	Both	[3, 5, 17, 18, 24, 25, 46]
		Only male	-
		Only female	[7]
		Unspecified	[10, 22]
	Immunosuppressive treatment	Yes	[7, 17]
		No/Unspecified	[3, 5, 10, 18, 22, 24, 25, 46]
	**Dosing**		**Reference numbers**
	Number of doses	Dose-escalation	[3, 7, 17, 24, 25]
		Single dose	[5, 10, 18, 24, 46]
		Repeated dose	[24, 25, 46]
		Unspecified	[22]
	Control groups	Positive	-
		Negative	[3, 5, 7, 10, 17, 18, 24, 25, 46]
		Spike-in	-
		None/Unspecified	[22]
	Transplantation site	Clinically intended	[3, 5, 7, 10, 17, 18, 24, 25, 46]
		Intravenous	[24]
		Unspecified	[22]
	**Study conditions**		**Reference numbers**
	GLP study	Yes	[3, 5, 7, 10]
		No/Unspecified	[17, 18, 22, 24, 25, 46]
	Outsourced	Yes	[3, 5, 7, 10, 25]
		No/Unspecified	[17, 18, 22, 24, 46]

GLP: good laboratory practice, WT: wild-type.

Table S13. Non-clinical efficacy studies of PSC-derived therapy products, related to Figure 4 and Figures S5-S6.

Study	Animal model		Reference numbers
Efficacy	Species	Mice (disease model)	[1, 8, 10, 20, 25, 32, 44, 45, 48]
		Mice (healthy)	[8, 17, 35, 44]
		Rats (disease model)	[3-5, 7, 10, 22, 26, 29]
		Rats (healthy)	[2]
		Rabbits	[24, 36, 46]
		Non-human primates	[17, 45]
		Pigs	[18]
	Male/Female ratio	Both	[4, 10, 17, 26, 29, 32]
		Only male	[8, 17, 20, 25, 36, 45, 48]
		Only female	[3, 5, 7, 18, 22]
		Unspecified	[1, 2, 24, 35, 44, 46]
	Immunosuppressive treatment	Yes	[3, 4, 7, 10, 17, 18, 24, 29, 36, 45, 46]
		No/Unspecified	[1, 2, 5, 8, 10, 17, 20, 22, 25, 26, 32, 35, 44, 45, 48]
	**Dosing**		**Reference numbers**
	Number of doses	Dose-escalation	[3, 5, 8, 17, 25, 29, 44, 45]
		Single dose	[1, 2, 4, 7, 10, 17, 18, 20, 22, 24, 26, 32, 35, 36, 45, 46, 48]
		Repeated dose	[10, 25, 32]
		Unspecified	-
	Control groups	Positive	[22, 24, 25, 32, 35, 48]
		Negative	[3-5, 7, 8, 10, 17, 18, 20, 22, 25, 26, 29, 32, 36, 44, 45, 48]
		Spike-in	-
		None/Unspecified	[1, 2, 46]
	Transplantation site	Clinically intended	[1-5, 7, 8, 10, 17, 18, 20, 22, 24-26, 29, 32, 35, 36, 45, 46]
		Kidney capsule	[44, 45]
		Intrasplenic	[48]
	**Study conditions**		**Reference numbers**
	GLP study	Yes	[36]
		No/Unspecified	[1-5, 7, 8, 10, 17, 18, 20, 22, 24-26, 29, 32, 35, 44-46, 48]
	Outsourced	Yes	[36, 45]
		No/Unspecified	[1-5, 7, 8, 10, 17, 18, 20, 22, 24-26, 29, 32, 35, 44, 46, 48]

GLP: good laboratory practice, WT: wild-type.

Table S14. CQAs tested and methods applied for clinical release testing of PSC-derived medicinal products, based on methods scored as QC and QCu.

QC	CQA	Method	Ref. no.
Identity	Marker expression	Flow cytometry	[3, 5, 6, 9, 18, 22, 24, 25, 37, 44, 45, 47]
		HLA expression (Flow cytometry)	[22, 24, 25]
		IF imaging	[6, 37]
		qPCR	[6, 27]
		PCR	[3]
	Morphology	Microscopic evaluation	[22, 25, 44, 45]
	Physiological	Differentiation potential	[22, 24]
		Dithizone (DTZ) staining	[44]
		Annexin V binding (Flow cytometry)	[47]
	Genotype	STR analysis	[5, 25, 44]
Impurities	Off-target cells	Flow cytometry	[3, 5, 6, 22, 24, 25, 44, 45, 47]
		qPCR	[6, 26]
		IF imaging	[6]
	Residual PSCs	Flow cytometry	[5, 9, 22, 25]
		qPCR	[3, 27]
		ddPCR	[37, 44]
		IF imaging	[25]
		PCR	[25]
		In vivo (mice)	[25]
	Transformed cells	SACF assay	[44]
	Residual raw materials	BSA	[25, 44, 47]
		Growth factors	[44]
		Small molecules	[47]
		Cytokines	[47]
	Interspecies contamination	STR analysis	[44]
		Isozyme analysis	[25]

Content	Viability	Automated cell counter	[3, 5, 9, 44]
		Fluorescence microscopy	[44]
		Flow cytometry	[45]
		Counting method unspecified	[18, 37]
	Cell count	Automated cell counter	[3, 5, 9]
		Flow cytometry	[47]
		Counting method unspecified	[37]
	General appearance	Visual inspection	[5, 37, 47]
	pH	Electrode method	[47]
Safety	Genomic integrity	Karyotyping	[22]
		WGS	[44]
		WES	[44]
	Adventitious virus	In vitro cell culture	[3, 5, 25, 44]
		Hemadsorption	[25, 44]
		Hemagglutination	[25, 44]
		qPCR (PCR-fluorescence probing)	[44]
		Ultracentrifugation and QFPERT assay	[5]
		In vivo	[25, 44]
		Method unspecified	[25, 45]
Potency	Hormone secretion	ELISA	[9, 44, 45]
	In vitro cell assays	Inhibition of PBMC proliferation	[24, 25]
		Dye transfer assay	[22]
	Marker expression	Flow cytometry	[47]
		IF imaging	[37]
	Morphological changes	In vitro tube formation assay	[22]
	Drug response	In vitro cell assay	[22]
	Substrate binding	Flow cytometry	[47]
Stability	In-use stability	Cell count	[47]
		General appearance	[47]
		pH	[47]
In vivo	Tumorigenicity (mice)	Gross organ observation	[19, 44]
		H&E staining	[19, 44]

HLA: human leukocyte antigen, H&E: hematoxylin and eosin, IF: immunofluorescence, PBMC: peripheral blood mononuclear cell, QFPERT: quantitative fluorescent product-enhanced reverse transcriptase, SACF: soft agar colony formation, STR: short tandem repeat.

Table S15. Tests applied in QC testing of PSC-derived cell therapy products grouped by cell type and specifications provided in the included reports. See Excel file.

Supplemental methods

A systematic search was conducted using pre-defined keywords to retrieve records on PSC-derived products and their QC, safety, and efficacy testing, manufacturing for clinical application, and regulatory guidelines (see section 'Systematic search strategy' below). Three databases were searched spanning the period from 1 January 2022 until 14 November 2024 (Figure S7). Titles and abstracts were screened on EndNote 21 by at least two reviewers independently: one primary reviewer screened the full set of retrieved records, followed by a parallel independent screening by one of the three secondary reviewers. Pre-defined criteria were used to include records on PSC-derived medicinal products developed for clinical application (for example, working with a clinical-grade cell line, implementing a GMP-compliant protocol, or mentioning the conduct of a clinical trial) and to exclude papers with a primary focus on adult stem cell products, exosomes, disease modelling, drug screening, developmental studies, or novel/pioneering techniques under development (see section 'Pre-defined inclusion/exclusion criteria' below). For the full text screening, reviews were removed first, and the remaining original publications were screened by the same primary and secondary reviewers. Four reports could not be retrieved because the trial registry records did not contain details about the quality testing. After exclusion due to the specified reasons, 41 reports remained.

To ensure the completeness of the retrieved reference list, the review from Kirkeby et al., 2025 [51] was used for cross-reference. Out of 61 listed clinical and non-clinical references from 2022-2024, 4 reports were included in the analysis after full-text screening [6, 8, 17, 32] (Figure S7). In addition, one relevant report was found independently (did not contain an abstract) [45] and 4 reports were added manually as specifically cross-referenced by the included articles to contain relevant additional information on the products [7, 10, 25, 29]. Finally, a total of 50 reports were included in the analysis.

The data extraction from the included articles was performed by the primary reviewer in close collaboration with the secondary reviewers. The data of interest were: (1) general information about the product and initiation of a clinical trial; (2) quality tests for product's identity, purity, content, safety, potency, and stability; and (3) *in vivo* studies for biodistribution, tumorigenicity, toxicity, and efficacy. Only the tests performed on end-differentiated cell products were extracted for this review. For the *in vitro* testing, the information of interest comprised of the utilized techniques and sensitivity, biomarkers, and source material of selected methods. For the *in vivo* studies, data were collected on the animal model (species, animal number, follow-up duration, sex, use of immunosuppression), dosing regimen

(number of doses, control groups, administration site), and study conditions (GLP, outsourcing). A ranking system was created to define the application of product quality tests across different stages of product development and clinical release (Table 1). The ranking was assigned based on the implementation of a testing method as described in a report. Similarly, the assignment of the test to a certain category were determined based on the primary goal of the testing method as defined in a report, for example, the test for detecting potentially tumorigenic impurities was categorized as impurity testing, while tests evaluating tumor formation as a safety risk fell under safety.

The relevant available information was extracted into an Excel sheet, summarized in table format, and subsequently transformed into graphical representations using GraphPad Prism 10.2.3. In cases where the *in vitro* or *in vivo* testing of the same medicinal product intended for the same indication was described in multiple reports, the test or study was counted once at the highest applied rank. There were six medicinal products covered by double reports among the included articles (Table S16), which usually was a result of separate publications on pre-clinical and clinical study reports. Durations of longest follow-up time points of *in vivo* studies specified in hours, days, weeks, or years were converted to months using an online tool Omni Calculator [52]. The relevant information was extracted where possible, and any missing or incomplete information (e.g. animal number or duration of *in vivo* studies) was treated as not given. Any differences in opinion during the screening and data extraction were resolved by consensus or referral to a third reviewer.

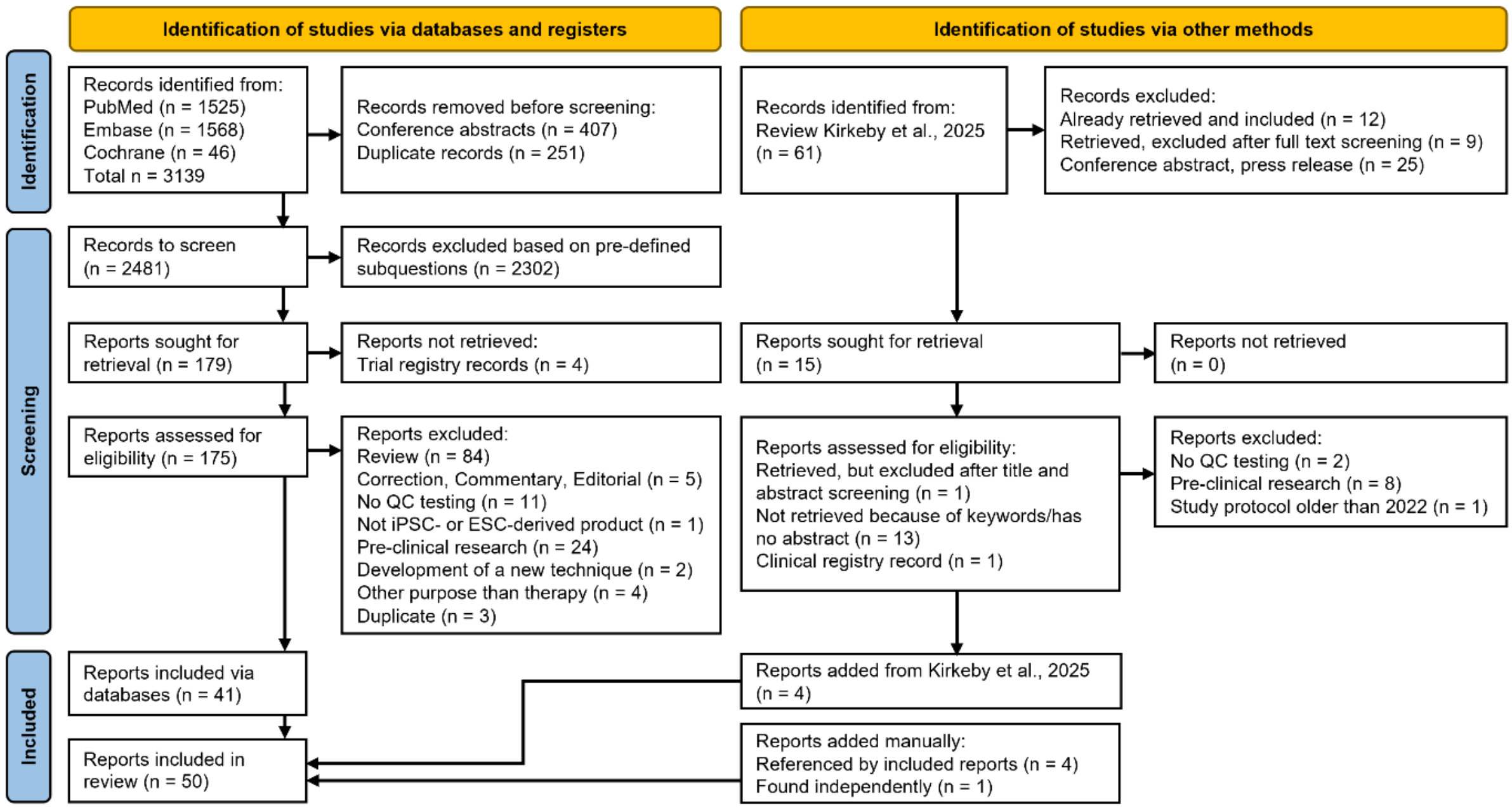

Figure S7. PRISMA 2020 flow diagram. The reports were collected via a systematic search and screening of databases, cross-referenced review paper, and manual addition. See also Supplemental methods.

Table S16. Medicinal products intended for the same indication covered by double reports. In the cases when a test or study was described in both reports, it was counted once at the highest applied rank (e.g. a test performed both as a characterization test during pre-clinical development, and in a later stage as a QC test with defined release specifications, this test was ranked as a QC test).

Medicinal products covered by double reports	Reference numbers
Cardiomyocytes (patches)	[18, 19]
Photoreceptors (retinal sheets)	[26, 27]
Astrocytes (AstroRx)	[9, 10]
RPE cells (suspension)	[28, 29]
Platelets (iPLAT1 clinical trial)	[46, 47]
Oligodendrocyte progenitor cells	[6, 7]

Systematic search strategy

PubMed:

("Stem cell-derived"[ti] OR "PSC-derived"[ti] OR "hPSC-derived"[ti] OR "iPSC-derived"[ti] OR "hiPSC-derived"[ti] OR "ESC-derived"[ti] OR "hESC-derived"[ti] OR "from pluripotent stem cell*"[tiab] OR "from human pluripotent stem cell*"[tiab] OR "from induced pluripotent stem cell*"[tiab] OR "from human induced pluripotent stem cell*"[tiab] OR "from embryonic stem cell*"[tiab] OR "from human embryonic stem cell*"[tiab] OR "from PSCs"[tiab] OR "from hPSCs"[tiab] OR "from human PSCs"[tiab] OR "from iPSCs"[tiab] OR "from hiPSCs"[tiab] OR "from human iPSCs"[tiab] OR "from ESCs"[tiab] OR "from hESCs"[tiab] OR "from human ESCs"[tiab] OR "residual stem cell*"[tiab] OR "residual PSC*"[tiab] OR "residual hPSC*"[tiab] OR "residual human PSC*"[tiab] OR "residual iPSC*"[tiab] OR "residual hiPSC*"[tiab] OR "residual human iPSC*"[tiab] OR "residual ESC*"[tiab] OR "residual hESC*"[tiab] OR "residual human ESC*"[tiab] OR "residual undifferentiated"[tiab])

AND

("Quality Control"[MeSH] OR "Total Quality Management"[MeSH] OR "Quality"[tiab] OR "QC test*"[tiab] OR "Chemistry, manufacturing and control"[tiab] OR "In-process control*"[tiab] OR "Good Manufacturing Practice"[tiab] OR "Clinical-grade"[tiab] OR "GMP-grade"[tiab] OR "GMP-compliant"[tiab] OR "Manufacturing"[tiab] OR "Product releas*"[tiab] OR "Process development"[tiab] OR ("Clinical"[tiab] AND "Trial*"[tiab]) OR ("Clinical"[tiab] AND "Stud*"[tiab]) OR "Consumer Product Safety"[MeSH] OR "Safety"[MeSH] OR "Safety"[tiab] OR "Carcinogenicity Tests"[MeSH] OR "Carcinogen*"[tiab] OR "Tumorigen*"[tiab] OR "Terato*"[tiab] OR "Genomic integrity"[tiab] OR "Genomic stability"[tiab] OR "Genomic instability"[tiab] OR "Efficac*"[tiab] OR "Potenc*"[tiab] OR "Characteri*"[tiab] OR "Legislation, Drug"[Mesh] OR "Policy"[Mesh] OR "United States Food and Drug Administration"[Mesh] OR "Legislat*"[tiab] OR "Policy"[tiab] OR "Policies"[tiab] OR "Regulation*"[tiab] OR "Guideline*"[tiab] OR "Guidance*"[tiab] OR "Regulatory standard*"[tiab] OR "Regulatory requirement*"[tiab])

Embase OVID:

(*induced pluripotent stem cell/ or *embryonic stem cell/ or ("Stem cell-derived" or "PSC-derived" or "hPSC-derived" or "iPSC-derived" or "hiPSC-derived" or "ESC-derived" or "hESC-derived").ti. or ("from pluripotent stem cell*" or "from human pluripotent stem cell*" or "from induced pluripotent stem cell*" or "from human induced pluripotent stem cell*" or "from embryonic stem cell*" or "from human embryonic stem cell*" or "from PSCs" or "from hPSCs" or "from human PSCs" or "from iPSCs" or "from hiPSCs" or "from human iPSCs" or "from ESCs" or "from hESCs" or "from human ESCs" or "residual stem cell*" or "residual PSC*" or "residual hPSC*" or "residual human PSC*" or "residual iPSC*" or "residual hiPSC*" or "residual human iPSC*" or "residual ESC*" or "residual hESC*" or "residual human ESC*" or "residual undifferentiated").ti,ab.) and (*quality control/ or *total quality management/ or *product safety/ or *patient safety/ or *drug safety/ or *good manufacturing practice/ or exp clinical trial/ or *drug efficacy/ or *drug potency/ or *carcinogen testing/ or *carcinogenesis/ or *carcinogenicity/ or *process development/ or *policy/ or *"food and drug administration"/ or *European Medicines Agency/ or *drug legislation/ or *drug approval/ or *genomic instability/ or ("Quality" or "QC test*" or "Chemistry, manufacturing and control" or "In-process control*" or "Good Manufacturing Practice" or ("Clinical" adj1 "Trial*") or ("Clinical" adj1 "Stud*") or "Safety" or "Efficac*" or "Potenc*" or "Characteri*" or "Carcinogen*" or "Tumorigen*" or "Terato*" or "Clinical-grade" or "GMP-grade" or "GMP-compliant" or "Manufacturing" or "Product releas*" or "Process development" or "Legislat*" or "Regulation*" or "Guideline*" or "Guidance*" or "Policy" or "Policies" or "Regulatory standard*" or "Regulatory requirement*" or "Genomic integrity" or "Genomic stability" or "Genomic instability").ti.)

Cochrane library:

ID Search

#1 ((pluripotent NEXT stem cell*) OR (induced NEXT pluripotent stem cell*) OR (embryonic NEXT stem cell*) OR (residual NEXT stem cell*) OR (residual NEXT PSC*) OR (residual NEXT hPSC*) OR (residual NEXT human PSC*) OR (residual NEXT human iPSC*) OR (residual NEXT iPSC*) OR (residual NEXT hiPSC*) OR (residual NEXT hESC*) OR (residual NEXT ESC*) OR (residual NEXT human ESC*) OR ("residual undifferentiated")):ti,ab,kw

#2 ("Stem cell-derived" or "PSC-derived" or "hPSC-derived" or "iPSC-derived" or "hiPSC-derived" or "ESC-derived" or "hESC-derived"):ti,ab,kw

#3 MeSH descriptor: [Induced Pluripotent Stem Cells] explode all trees

#4 MeSH descriptor: [Embryonic Stem Cells] explode all trees

#5 MeSH descriptor: [Pluripotent Stem Cells] explode all trees

#6 {OR #1-#5}

#7 MeSH descriptor: [Quality Control] explode all trees

#8 MeSH descriptor: [Total Quality Management] explode all trees

#9 MeSH descriptor: [Consumer Product Safety] explode all trees

#10 MeSH descriptor: [Carcinogenicity Tests] explode all trees

#11 MeSH descriptor: [Carcinogenesis] explode all trees

#12 MeSH descriptor: [Safety] explode all trees

#13 MeSH descriptor: [Legislation, Drug] explode all trees

#14 MeSH descriptor: [Drug Evaluation] explode all trees

#15 MeSH descriptor: [Drug Evaluation, Preclinical] explode all trees

#16 MeSH descriptor: [Policy] explode all trees

#17 MeSH descriptor: [United States Food and Drug Administration] explode all trees

#18 MeSH descriptor: [Clinical Trial] explode all trees

#19 MeSH descriptor: [Genomic Instability] explode all trees

#20 (("Quality" or (QC NEXT test*) or "Chemistry manufacturing and control" or (In-process NEXT control*) or (Good Manufacturing NEXT Practice*) or (Clinical NEXT Trial*) or (Clinical NEXT Stud*) or "Safety" or Efficac* or Potenc* or Characteri* or Carcinogen* or Tumorigen* or "Clinical-grade" or "GMP-grade" or "GMP-compliant" or "Manufacturing" or (Product NEXT releas*) or "Process development" or Legislat* or Regulation* or Guideline* or Guidance* or "Policy" or "Policies" or (Regulatory NEXT standard*) or (Regulatory NEXT requirement*) or "Genomic integrity" or "Genomic stability" OR Terato*)):ti,ab,kw

#21 {OR #7-#20}

#22 #6 AND #21

Pre-defined inclusion/exclusion criteria

Include, if the answer is “Yes”:

1. Does the paper discuss **stem cell-derived** therapy to be used in the clinic (e.g. mentions "clinical-grade", "GMP-compliant", "clinical trial")?
    a. Does the paper discuss **safety** of stem cell-based therapy?
    b. Does the paper discuss the measures to prevent **tumorigenicity** of stem cell-based therapy?
    c. Does the paper mention **biodistribution** studies?
    d. Does the paper mention **cell identity/composition** testing of a cell therapy product?
    e. Does the paper mention **purity** testing of a cell therapy product?
    f. Does the paper mention **potency** testing of a cell therapy product?
    g. Does the paper mention detection/removal of **residual stem cells**?

Exclude, if the answer is “Yes”:

1. Is the main focus of the paper a **stem cell therapy**?
2. Is the main focus of the paper a cell therapy derived from the stem cells other than iPSCs or ESCs (e.g. **mesenchymal stem cell-derived)**?
3. Is the main focus of the paper stem cell-derived **exosomes**?
4. Is the main focus of the paper **disease modelling** (using organoids, organs-on-chips)?
5. Is the main focus of the paper **drug screening** using stem cells or their products?
6. Is the main focus of the paper **developmental studies** (organism/organ development)?
7. Is the main focus of the paper **a novel strategy under development** for the detection/removal of **residual stem cells** that is **pre-clinical**?
8. Is the main focus of the paper **a novel test under development** for a stem cell-derived product that is **pre-clinical**?

Supplemental references

[1] M. Luciani, C. Garsia, E. Mangiameli, V. Meneghini, and A. Gritti, "Intracerebroventricular transplantation of human iPSC-derived neural stem cells (hiPSC-NSCs) into neonatal mice," (in eng), *Methods Cell Biol,* vol. 171, pp. 127-147, 2022, doi: 10.1016/bs.mcb.2022.04.007.

[2] M. Shigyo *et al.*, "Derivation of Sendai-Virus-Reprogrammed Human iPSCs-Neuronal Precursors: In Vitro and In Vivo Post-grafting Safety Characterization," (in English), *Cell Transplantation,* vol. 32, no. no pagination, 2023, doi: https://dx.doi.org/10.1177/09636897231163232.

[3] S. Park *et al.*, "Preclinical and dose-ranging assessment of hESC-derived dopaminergic progenitors for a clinical trial on Parkinson's disease," (in English), *Cell Stem Cell,* vol. 31(1), pp. 25-38.e8, 04 Jan 2024, doi: https://dx.doi.org/10.1016/j.stem.2023.11.009.

[4] S. Naderi *et al.*, "Cryopreserved clinical-grade human embryonic stem cell-derived dopaminergic progenitors function in Parkinson's disease models," (in English), *Life Sciences,* vol. 329(no pagination), 15 Sep 2023, Art no. 121990, doi: https://dx.doi.org/10.1016/j.lfs.2023.121990.

[5] A. Kirkeby *et al.*, "Preclinical quality, safety, and efficacy of a human embryonic stem cell-derived product for the treatment of Parkinson's disease, STEM-PD," (in English), *Cell Stem Cell,* vol. 30(10), pp. 1299-1314.e9, 05 Oct 2023, doi: https://dx.doi.org/10.1016/j.stem.2023.08.014.

[6] R. G. Fessler *et al.*, "A phase 1/2a dose-escalation study of oligodendrocyte progenitor cells in individuals with subacute cervical spinal cord injury," *J Neurosurg Spine,* vol. 37, no. 6, pp. 812-820, Dec 1 2022, doi: 10.3171/2022.5.SPINE22167.

[7] N. C. Manley, C. A. Priest, J. Denham, E. D. Wirth, 3rd, and J. S. Lebkowski, "Human Embryonic Stem Cell-Derived Oligodendrocyte Progenitor Cells: Preclinical Efficacy and Safety in Cervical Spinal Cord Injury," *Stem Cells Transl Med,* vol. 6, no. 10, pp. 1917-1929, Oct 2017, doi: 10.1002/sctm.17-0065.

[8] M. Bershteyn *et al.*, "Human pallial MGE-type GABAergic interneuron cell therapy for chronic focal epilepsy," *Cell Stem Cell,* vol. 30, no. 10, pp. 1331-1350 e11, Oct 5 2023, doi: 10.1016/j.stem.2023.08.013.

[9] M. Gotkine *et al.*, "Safety and efficacy of first-in-man intrathecal injection of human astrocytes (AstroRx) in ALS patients: phase I/IIa clinical trial results," (in English), *Journal of Translational Medicine,* vol. 21(1) (no pagination), December 2023, Art no. 122, doi: https://dx.doi.org/10.1186/s12967-023-03903-3.

[10] M. Izrael *et al.*, "Safety and efficacy of human embryonic stem cell-derived astrocytes following intrathecal transplantation in SOD1(G93A) and NSG animal models," *Stem Cell Res Ther,* vol. 9, no. 1, p. 152, Jun 6 2018, doi: 10.1186/s13287-018-0890-5.

[11] J. W. Park, S. J. Bae, J. H. Yun, S. Kim, and M. Park, "Assessment of Genetic Stability in Human Induced Pluripotent Stem Cell-Derived Cardiomyocytes by Using Droplet Digital PCR," *Int J Mol Sci,* vol. 25, no. 2, Jan 16 2024, doi: 10.3390/ijms25021101.

[12] T. Watanabe *et al.*, "International evaluation study of a highly efficient culture assay for detection of residual human pluripotent stem cells in cell therapies," (in English), *Regenerative Medicine,* vol. 18(3), pp. 219-227, 01 Mar 2023, doi: https://dx.doi.org/10.2217/rme-2022-0207.

[13] O. Sekine *et al.*, "Seamless and non-destructive monitoring of extracellular microRNAs during cardiac differentiation from human pluripotent stem cells," *Stem Cell Reports,* vol. 18, no. 10, pp. 1925-1939, Oct 10 2023, doi: 10.1016/j.stemcr.2023.08.011.

[14] M. Lemmens *et al.*, "Identification of marker genes to monitor residual iPSCs in iPSC-derived products," *Cytotherapy,* vol. 25, no. 1, pp. 59-67, Jan 2023, doi: 10.1016/j.jcyt.2022.09.010.

[15] S. Yasuda *et al.*, "Detection of residual pluripotent stem cells in cell therapy products utilizing droplet digital PCR: an international multisite evaluation study," *Stem Cells Transl Med,* vol. 13, no. 10, pp. 1001-1014, Oct 10 2024, doi: 10.1093/stcltm/szae058.

[16] N. Kriedemann *et al.*, "Standardized production of hPSC-derived cardiomyocyte aggregates in stirred spinner flasks," (in eng), *Nat Protoc,* Mar 28 2024, doi: 10.1038/s41596-024-00976-2.

[17] H. Kobayashi *et al.*, "Regeneration of Nonhuman Primate Hearts With Human Induced Pluripotent Stem Cell-Derived Cardiac Spheroids," *Circulation,* vol. 150, no. 8, pp. 611-621, Aug 20 2024, doi: 10.1161/CIRCULATIONAHA.123.064876.

[18] S. Miyagawa *et al.*, "Pre-clinical evaluation of the efficacy and safety of human induced pluripotent stem cell-derived cardiomyocyte patch," (in English), *Stem Cell Research and Therapy,* vol. 15(1) (no pagination), December 2024, Art no. 73, doi: https://dx.doi.org/10.1186/s13287-024-03690-8.

[19] S. Miyagawa *et al.*, "Case report: Transplantation of human induced pluripotent stem cell-derived cardiomyocyte patches for ischemic cardiomyopathy," *Front Cardiovasc Med,* vol. 9, p. 950829, 2022, doi: 10.3389/fcvm.2022.950829.

[20] L. Rong *et al.*, "Clinical-grade human embryonic stem cell-derived mesenchymal stromal cells ameliorate diabetic retinopathy in db/db mice," (in English), *Cytotherapy.,* 2024, doi: https://dx.doi.org/10.1016/j.jcyt.2024.02.020.

[21] Q. Zhao and F. Liu, "Derivation and Characterization of Mesenchymal Stem Cells from iPS Cells," (in English), *Methods in Molecular Biology,* Chapter vol. 2454, pp. 443-454, 2022, doi: https://dx.doi.org/10.1007/7651_2020_327.

[22] J. H. Shin *et al.*, "Safety of Human Embryonic Stem Cell-derived Mesenchymal Stem Cells for Treating Interstitial Cystitis: A Phase I Study," (in English), *Stem Cells Translational Medicine,* vol. 11(10), pp. 1010-1020, October 2022, doi: https://dx.doi.org/10.1093/stcltm/szac065.

[23] T. Watanabe *et al.*, "Multisite studies for optimization of a highly efficient culture assay used for in vitro detection of residual undifferentiated human pluripotent stem cells intermingled in cell therapy products," *Regen Ther,* vol. 26, pp. 315-323, Jun 2024, doi: 10.1016/j.reth.2024.06.007.

[24] L. Huang *et al.*, "Immunity-and-matrix-regulatory cells enhance cartilage regeneration for meniscus injuries: a phase I dose-escalation trial," (in English), *Signal Transduction and Targeted Therapy,* vol. 8(1) (no pagination), December 2023, Art no. 417, doi: https://dx.doi.org/10.1038/s41392-023-01670-7.

[25] J. Wu *et al.*, "Immunity-and-matrix-regulatory cells derived from human embryonic stem cells safely and effectively treat mouse lung injury and fibrosis," *Cell Res,* vol. 30, no. 9, pp. 794-809, Sep 2020, doi: 10.1038/s41422-020-0354-1.

[26] K. Watari *et al.*, "Self-organization, quality control, and preclinical studies of human iPSC-derived retinal sheets for tissue-transplantation therapy," (in English), *Communications biology,* vol. 6(1), p. 164, 10 Feb 2023, doi: https://dx.doi.org/10.1038/s42003-023-04543-5.

[27] Y. Hirami *et al.*, "Safety and stable survival of stem-cell-derived retinal organoid for 2 years in patients with retinitis pigmentosa," (in English), *Cell Stem Cell,* vol. 30(12), pp. 1585-1596.e6, 07 Dec 2023, doi: https://dx.doi.org/10.1016/j.stem.2023.11.004.

[28] H. Surendran *et al.*, "An improved protocol for generation and characterization of human-induced pluripotent stem cell-derived retinal pigment epithelium cells," (in English), *STAR protocols,* vol. 3(4), p. 101803, 16 Dec 2022, doi: https://dx.doi.org/10.1016/j.xpro.2022.101803.

[29] H. Surendran *et al.*, "Transplantation of retinal pigment epithelium and photoreceptors generated concomitantly via small molecule-mediated differentiation rescues visual function in rodent models of retinal degeneration," *Stem Cell Res Ther,* vol. 12, no. 1, p. 70, Jan 19 2021, doi: 10.1186/s13287-021-02134-x.
[30] H. Y. Jo *et al.*, "Single-Cell RNA Sequencing of Human Pluripotent Stem Cell-Derived Macrophages for Quality Control of The Cell Therapy Product," (in English), *Frontiers in Genetics,* vol. 12(no pagination), 31 Jan 2022, Art no. 658862, doi: https://dx.doi.org/10.3389/fgene.2021.658862.
[31] L. Chung, L. A. Cogburn, L. Sui, and J. L. Dashnau, "Development of an induced pluripotent stem cell-specific microRNA assay for detection of residual undifferentiated cells in natural killer cell therapy products," *Cytotherapy,* vol. 24, no. 7, pp. 733-741, Jul 2022, doi: 10.1016/j.jcyt.2022.02.005.
[32] F. Cichocki *et al.*, "Quadruple gene-engineered natural killer cells enable multi-antigen targeting for durable antitumor activity against multiple myeloma," *Nat Commun,* vol. 13, no. 1, p. 7341, Nov 29 2022, doi: 10.1038/s41467-022-35127-2.
[33] J. Sun *et al.*, "Analysis of three characterization assays reveals ddPCR of LIN28A as the most sensitive for the detection of residual pluripotent stem cells in cellular therapy products," *Cytotherapy,* vol. 26, no. 11, pp. 1374-1381, Nov 2024, doi: 10.1016/j.jcyt.2024.05.019.
[34] S. Domingues *et al.*, "Clinical Grade Human Pluripotent Stem Cell-Derived Engineered Skin Substitutes Promote Keratinocytes Wound Closure In Vitro," (in English), *Cells,* vol. 11(7) (no pagination), April-1 2022, Art no. 1151, doi: https://dx.doi.org/10.3390/cells11071151.
[35] G. Neumayer *et al.*, "A scalable and cGMP-compatible autologous organotypic cell therapy for Dystrophic Epidermolysis Bullosa," *Nat Commun,* vol. 15, no. 1, p. 5834, Jul 11 2024, doi: 10.1038/s41467-024-49400-z.
[36] J. Yu *et al.*, "Safety and efficacy of human ESC-derived corneal endothelial cells for corneal endothelial dysfunction," (in English), *Cell and Bioscience,* vol. 13(1) (no pagination), December 2023, Art no. 201, doi: https://dx.doi.org/10.1186/s13578-023-01145-w.
[37] T. Soma *et al.*, "Induced pluripotent stem-cell-derived corneal epithelium for transplant surgery: a single-arm, open-label, first-in-human interventional study in Japan," (in eng), *Lancet,* Nov 7 2024, doi: 10.1016/s0140-6736(24)01764-1.
[38] M. Corsini and S. Mitola, "Alternative In Vivo Models to Study Teratoma," (in English), *Methods in Molecular Biology,* Chapter vol. 2572, pp. 181-189, 2023, doi: https://dx.doi.org/10.1007/978-1-0716-2703-7_14.
[39] H. Shi, T. Feng, R. Wang, L. Wu, and Y. Gu, "Universal Markers for hiPSCs Residue Detection," *Front Biosci (Landmark Ed),* vol. 27, no. 8, p. 239, Aug 11 2022, doi: 10.31083/j.fbl2708239.
[40] S. Oh *et al.*, "Tumorigenicity Assessment of Human Cancer Cell Lines Xenografted on Immunodeficient Mice as Positive Controls of Tumorigenicity Testing," (in English), *International Journal of Toxicology,* vol. 41(6), pp. 476-487, December 2022, doi: https://dx.doi.org/10.1177/10915818221124573.
[41] K. Masumoto *et al.*, "Highly sensitive and non-disruptive detection of residual undifferentiated cells by measuring miRNAs in culture supernatant," (in eng), *Sci Rep,* vol. 12, no. 1, p. 10351, Jun 20 2022, doi: 10.1038/s41598-022-14273-z.
[42] D. G. Iworima *et al.*, "Metabolic switching, growth kinetics and cell yields in the scalable manufacture of stem cell-derived insulin-producing cells," (in eng), *Stem Cell Res Ther,* vol. 15, no. 1, p. 1, Jan 2 2024, doi: 10.1186/s13287-023-03574-3.
[43] Y. Wu, Z. Zhang, S. Wu, Z. Chen, and Y. Pu, "Estimating residual undifferentiated cells in human chemically induced pluripotent stem cell derived islets using lncRNA as

biomarkers," *Sci Rep,* vol. 13, no. 1, p. 16435, Sep 30 2023, doi: 10.1038/s41598-023-43798-0.
[44] S. Wang *et al.*, "Transplantation of chemically induced pluripotent stem-cell-derived islets under abdominal anterior rectus sheath in a type 1 diabetes patient," (in eng), *Cell,* vol. 187, no. 22, pp. 6152-6164.e18, Oct 31 2024, doi: 10.1016/j.cell.2024.09.004.
[45] J. Wu *et al.*, "Treating a type 2 diabetic patient with impaired pancreatic islet function by personalized endoderm stem cell-derived islet tissue," *Cell Discov,* vol. 10, no. 1, p. 45, Apr 30 2024, doi: 10.1038/s41421-024-00662-3.
[46] N. Sugimoto *et al.*, "Production and nonclinical evaluation of an autologous iPSC-derived platelet product for the iPLAT1 clinical trial," (in English), *Blood Advances,* vol. 6(23), pp. 6056-6069, 13 Dec 2022, doi: https://dx.doi.org/10.1182/bloodadvances.2022008512.
[47] N. Sugimoto *et al.*, "iPLAT1: the first-in-human clinical trial of iPSC-derived platelets as a phase 1 autologous transfusion study," (in English), *Blood,* vol. 140(22), pp. 2398-2402, 01 Dec 2022, doi: https://dx.doi.org/10.1182/blood.2022017296.
[48] M. Gantier *et al.*, "Cryopreserved cGMP-compliant human pluripotent stem cell-derived hepatic progenitors rescue mice from acute liver failure through rapid paracrine effects on liver cells," (in eng), *Stem Cell Res Ther,* vol. 15, no. 1, p. 71, Mar 12 2024, doi: 10.1186/s13287-024-03673-9.
[49] H. Tsujimoto *et al.*, "In vitro methods to ensure absence of residual undifferentiated human induced pluripotent stem cells intermingled in induced nephron progenitor cells," (in eng), *PLoS One,* vol. 17, no. 11, p. e0275600, 2022, doi: 10.1371/journal.pone.0275600.
[50] M. E. Diaz-Hernandez, N. M. Khan, and H. Drissi, "Efficient Differentiation of Human Induced Pluripotent Stem Cell (hiPSC)-Derived Mesenchymal Progenitors Into Adipocytes and Osteoblasts," (in eng), *Bio Protoc,* vol. 13, no. 22, p. e4885, Nov 20 2023, doi: 10.21769/BioProtoc.4885.
[51] A. Kirkeby, H. Main, and M. Carpenter, "Pluripotent stem-cell-derived therapies in clinical trial: A 2025 update," *Cell Stem Cell,* vol. 32, no. 1, pp. 10-37, Jan 2 2025, doi: 10.1016/j.stem.2024.12.005.
[52] "Omni Calculator." https://www.omnicalculator.com/ (accessed [Accessed August 7, 2025.
