# Supplementary material for: Quality control and non-clinical testing of iPSC- and ESC-derived medicinal products: A systematic review
Source: Stem Cell Reports. 2026 Sep 24;21(10):103084. doi: 10.1016/j.stemcr.2026.103084 (PMC13681315; doi:10.1016/j.stemcr.2026.103084)
Supplement: Supplementary file 2 [file mmc3.pdf]

STEM CELL REPORTS ISSCR
Review
OPEN ACCESS

Quality control and non-clinical testing of iPSC- and ESC-derived medicinal products: A systematic review

Akvilė Gasiūnaitė,[1,2] Mara Tihaya,[1] Inge van Amerongen-Westra,[1] Fong Lin,[1] Pema Gurung,[3] Bahareh Rajaei,[2] Eelco de Koning,[2] Françoise Carlotti,[2] and Pauline Meij[1,*]
[1]Center for Cell and Gene Therapy, Leiden University Medical Center, Leiden, the Netherlands
[2]Department of Internal Medicine, Leiden University Medical Center, Leiden, the Netherlands
[3]Walaeus Library, Leiden University Medical Center, Leiden, the Netherlands
*Correspondence: p.meij@lumc.nl
https://doi.org/10.1016/j.stemcr.2026.103084

SUMMARY

The lack of specific resources for quality control (QC) and non-clinical testing of cell-based medicinal products derived from embryonic and induced pluripotent stem cells (PSCs) contributes to uncertainty, variability, and delay in devising testing strategies. We conducted a systematic review on testing practices for diverse PSC-derived medicinal products using a custom-made ranking system, compiling a comprehensive "QC menu" of relevant critical quality attributes, established fit-for-purpose and promising techniques, and study designs for product development or clinical release. This consolidated resource could benefit product developers and regulators by creating informative testing strategies, ultimately increasing harmonization, facilitating clinical translation, and ensuring patient safety.

INTRODUCTION

Advanced therapy medicinal products are medicines mostly based on living cells and tissues. Increasingly, they are generated by differentiating pluripotent stem cells (PSCs), such as embryonic stem cells (ESCs) and induced PSCs (iPSCs) (Kirkeby et al., 2025; Kobold et al., 2023), which offer a renewable source of starting material for manufacturing. PSC-derived cell medicinal products were first introduced into the clinic 16 years ago (McKenna et al., 2022), and an increase of initiated clinical trials is observed in the last quinquennium, exceeding 100 studies in 2024 (Kirkeby et al., 2025).

PSC-derived therapies need to be thoroughly tested during pre-clinical development, manufacturing, and release to ensure quality, efficacy, and safety. Physical, chemical, or (micro)biological properties of a drug substance that describe and/or influence quality of the (investigational) medicinal product are called critical quality attributes (CQAs) (ICH, 2009). CQAs should meet appropriate specifications monitored via a set of quality control (QC) tests, which are performed on each product batch prior to release (ICH, 2009). While appropriate CQAs, acceptance criteria, and standardized testing methods are well-established in guidelines for conventional chemical pharmaceuticals (ICH, 1999), there are few detailed resources that can be consulted when designing a testing strategy for PSC-derived therapies (Barry et al., 2026; Hirai et al., 2023; Sato et al., 2019). Guidance for testing cell banks, including PSCs, has been established in regulatory guidelines (ICH, 1997, 2023) and consensus workshops (Sullivan et al., 2018); however, recommendations for testing PSC-derived end-products are less clearly defined (Barry et al., 2026; EMA, 2011; Hayakawa et al., 2015a; 2015b; 2015c), with the exception of compendial microbiological sterility, mycoplasma, and endotoxin tests, which are common across all aseptically produced medicinal products (EDQM (European Directorate for the Quality of Medicines & HealthCare), 2025a, 2025b, 2025c, 2025d, 2025e). A recently published document developed by the International Society for Stem Cell Research (ISSCR) has synthesized a more specific translational roadmap, taking into account the guidelines from major regional and international regulatory authorities, aiming to highlight the appropriate CQAs and suitable testing strategies for determining identity, purity, safety, potency, and stability of PSC-derived medicinal products (Barry et al., 2026).

The lack of specific guidance for PSC-derived therapies stems from the novelty, complexity, and diversity of these versatile products (Heslop et al., 2015). Although progressive insights on suitable testing strategies are gained with every new product entering clinical trials, the translatability of a QC strategy from one PSC-derived product to another is often limited due to differences in target cell types and product characteristics, necessitating custom development of quality assurance measures. Regulators evaluate the quality testing strategies on a case-by-case basis, relying on a risk-based approach to tailor the design of QC tests to the specific characteristics of the product (EMA, 2008). It is important for both the product developers and the regulators to have a comprehensive overview of the latest developments in the PSC-derived therapy field to design and evaluate suitable QC and non-clinical testing strategies for a successful translation of the product to the clinic.

Our paper focuses on the end-differentiated PSC derivatives and provides an in-depth overview of the currently applied practices for QC and non-clinical testing of the final products. We conducted a systematic search,

Published by Elsevier Inc. on behalf of International Society for Stem Cell Research.
This is an open access article under the CC BY license (http://creativecommons.org/licenses/by/4.0/).

extracting quality testing strategies and key parameters in the non-clinical study design implemented for ESC- and iPSC-derived products in the period of 2022–2024 to reflect the practices within a recent period in this fast-paced and innovative field. We used a custom-made ranking system to discriminate tests used for product characterization during the pre-clinical developmental phase, and tests that either have been used or were proposed as a QC release test in a clinical setting. Our aim was to systematically map the quality testing practices applied for PSC-derived medicinal products and to develop a ranked "QC menu" of methods with proven and promising suitability to assess a wide range of CQAs. This comprehensive resource of existing QC and non-clinical testing practices can thus complement the existing guidelines with a list of applicable techniques and support the design of future testing strategies, leading to higher quality PSC-derived medicinal products and, ultimately, patient safety.

RESULTS

General characteristics of included reports

Fifty reports were included from 2022 to 2024, covering a variety of ESC- and iPSC-derived products developed for diverse disease indications (Bershteyn et al., 2023; Chung et al., 2022; Cichocki et al., 2022; Corsini and Mitola, 2023; Diaz-Hernandez et al., 2023; Domingues et al., 2022; Fessler et al., 2022; Gantier et al., 2024; Gotkine et al., 2023; Hirami et al., 2023; Huang et al., 2023; Iworima et al., 2024; Izrael et al., 2018; Jo et al., 2022; Kirkeby et al., 2023; Kobayashi et al., 2024; Kriedemann et al., 2024; Lemmens et al., 2023; Luciani et al., 2022; Manley et al., 2017; Masumoto et al., 2022; Miyagawa et al., 2022; Miyagawa et al., 2024; Naderi et al., 2023; Neumayer et al., 2024; Oh et al., 2022; Park et al., 2024a, 2024b; Rong et al., 2024; Sekine et al., 2023; Shi et al., 2022; Shigyo et al., 2023; Shin et al., 2022; Soma et al., 2024; Sugimoto et al., 2022a, 2022b; Sun et al., 2024; Surendran et al., 2021, 2022; Tsujimoto et al., 2022; Wang et al., 2024; Watanabe et al., 2023, 2024; Watari et al., 2023; Wu et al., 2020, 2023, 2024; Yasuda et al., 2024; Yu et al., 2023; Zhao and Liu, 2022) (Figure S1A; Table S1). In this dataset, reports on neuronal cells, cardiomyocytes, and mesenchymal stromal/stem cells (MSCs) collectively accounted for approximately half of all entries, underscoring the prominence of these medicinal products in current research. Four reports were designed to develop universal techniques that could be applicable for products of multiple lineages (Corsini and Mitola, 2023; Masumoto et al., 2022; Oh et al., 2022; Shi et al., 2022). Furthermore, almost half of the reports described products or techniques that were used in clinical trials (Figure S1B) in Asia (13 unique clinical trials) (Gotkine et al., 2023; Hirami et al., 2023; Huang et al., 2023; Izrael et al., 2018; Kobayashi et al., 2024; Miyagawa et al., 2022, 2024; Park et al., 2024b; Rong et al., 2024; Shin et al., 2022; Soma et al., 2024; Sugimoto et al., 2022a, 2022b; Surendran et al., 2021, 2022; Wang et al., 2024; Watari et al., 2023; Wu et al., 2020, 2024), the United States (3) (Bershteyn et al., 2023; Cichocki et al., 2022; Fessler et al., 2022; Manley et al., 2017), and Europe (1) (Kirkeby et al., 2023). The remaining reports focused on technique validation, development of manufacturing protocols and QC assays, and product characterization to support clinical translation (Chung et al., 2022; Corsini and Mitola, 2023; Diaz-Hernandez et al., 2023; Domingues et al., 2022; Gantier et al., 2024; Iworima et al., 2024; Jo et al., 2022; Kriedemann et al., 2024; Lemmens et al., 2023; Luciani et al., 2022; Masumoto et al., 2022; Naderi et al., 2023; Neumayer et al., 2024; Oh et al., 2022; Park et al., 2024a; Sekine et al., 2023; Shi et al., 2022; Shigyo et al., 2023; Sun et al., 2024; Tsujimoto et al., 2022; Watanabe et al., 2023, 2024; Wu et al., 2023; Yasuda et al., 2024; Yu et al., 2023; Zhao and Liu, 2022).

Identity

Tests used to identify and quantify the target cell population were grouped according to the investigated CQA and scored in accordance with the created ranking system (Tables 1 and S2; Figure 1A). The ranking system was based on terms widely used for testing of medicinal products: characterization performed as extensive testing for establishing product and process understanding during product development (Ch); QC testing for decision-making purposes during release ensuring quality of the medicinal product (QC) (ICH, 2008); and supportive testing during the release of the product performed For Information Only (FIO) (Madrid et al., 2024). The ranking was determined based on the application of a testing method as described in a report. Release tests for clinical application with or without defined specifications were ranked as "QC" or "QCu" (undefined), respectively. FIO tests used for the release of a product for clinical application were ranked "FIO", whereas potential/proposed QC and FIO tests for intended clinical application were ranked "pQC" and "pFIO". Lastly, tests used for the characterization during product development, but not intended for use as release tests, were ranked "Ch" (Table 1).

Marker expression was the most frequently evaluated cell attribute with flow cytometry as the leading technique utilized to determine the identity of the product, both for general characterization and release testing (Figure 1A). PCR techniques and immunofluorescence (IF) imaging were used to analyze RNA and protein expression, respectively, both qualitatively, by gel electrophoresis or observing positive staining, as well as quantitatively, by qPCR and

Table 1. Ranking system of quality tests

Rank	Definition
QC	A test used as a QC for release of a product for clinical application with specific acceptance criteria.
QCu (QC undefined)	A test used as a QC for release of a product for clinical application, however, the specific acceptance criteria are not mentioned (e.g., markers for target cell identity were defined and results were given but not the acceptance criteria).
FIO	A test used during the release of a product for clinical application For Information Only.
pQC (proposed/potential QC)	A test/property defined as a QC/CQA of a product but not employed in the product release for a clinical application yet. It may not have the specific acceptance criteria.
pFIO (proposed/potential FIO)	A test proposed as optional yet supportive but not employed in the product release for clinical application yet.
Ch (characterization)	A test used during the development and characterization of the product and not falling under the definitions above.

counting positive cells. Bulk (Naderi et al., 2023; Neumayer et al., 2024; Surendran et al., 2021; Wu et al., 2020; Yu et al., 2023) and single cell (Bershteyn et al., 2023; Jo et al., 2022; Miyagawa et al., 2024; Neumayer et al., 2024; Wang et al., 2024; Wu et al., 2020, 2024) RNA sequencing was mostly used for characterization; however, one report has proposed to use single cell RNA sequencing for QC assessment (Jo et al., 2022).

The next CQA that was most often taken into consideration was cell or aggregate morphology. The most common method was microscopic evaluation without staining to observe the morphology and size of individual cells or cell clusters. Hematoxylin and eosin (H&E) and immunohistochemistry (IHC) staining were used to assess tissue-engineered constructs for presence of cell multilayers, subcellular structures, or cell polarization. Transmission electron microscopy was used to observe platelet ultrastructures (Sugimoto et al., 2022a) and subcellular structures and cell-cell connections in cardiomyocyte patches (Miyagawa et al., 2024), while a particle size analyzer was employed to evaluate platelet maturity (Sugimoto et al., 2022a).

Metabolic parameters have been proposed as a CQA in pancreatic islet cells, where the consumption of nutrients and production of certain metabolites were suggested to indicate the required switch toward the intended cell type (Iworima et al., 2024). Specifically, decreasing glucose consumption and lactate accumulation rates indicated the transition from glycolysis to oxidative phosphorylation during the differentiation of ESCs into pancreatic islets, while decreased C-peptide accumulation in spent medium at end differentiation stage could indicate the shift from primarily constitutive to more regulated hormone secretion, representing beta cell maturation (Iworima et al., 2024).

Several tests that were used to verify the cellular identity as a CQA were related to their physiology and therefore included a variety of methods to assess functional characteristics. For example, MSC identity was confirmed by trilineage (osteogenic, chondrogenic, and adipogenic) differentiation potential verified by cell morphology, IF imaging, and/or qPCR (Huang et al., 2023; Rong et al., 2024; Shin et al., 2022; Wu et al., 2020). This ability was evaluated to confirm the physiological identity of the product, rather than potency, as the intended therapeutic mechanism was not directly related to the differentiation capacity, but was based on the immunomodulatory properties of MSCs for the treatment of joint, lung, bladder, and retinal diseases (Huang et al., 2023; Rong et al., 2024; Shin et al., 2022; Wu et al., 2020).

Finally, donor identity confirmation is required to match the differentiated product to the correct source. For this CQA, the cell product's genotype was compared with the Master Cell Bank and/or the patient/donor cells for allogeneic (Kirkeby et al., 2023; Wu et al., 2020) and autologous (Wang et al., 2024) products. This assessment was applied as a QC test by performing a short tandem repeat (STR) analysis using PCR technique in three reports (Kirkeby et al., 2023; Wang et al., 2024; Wu et al., 2020).

To sum up, target cell identity was confirmed in a variety of ways by assessing protein and RNA expression, cell and aggregate morphology, metabolism, and physiological characteristics, as well as a match with the donor material. While some techniques were product-specific (e.g., antigen recognition assay for platelets), others were universal and used as QC tests for a variety of product types (e.g., flow cytometry, bright-field and fluorescence microscopy, and qPCR).

Impurities

The purity evaluation of PSC-derived products was based on identifying and quantifying cellular and acellular impurities, which can be divided into four subgroups: off-target

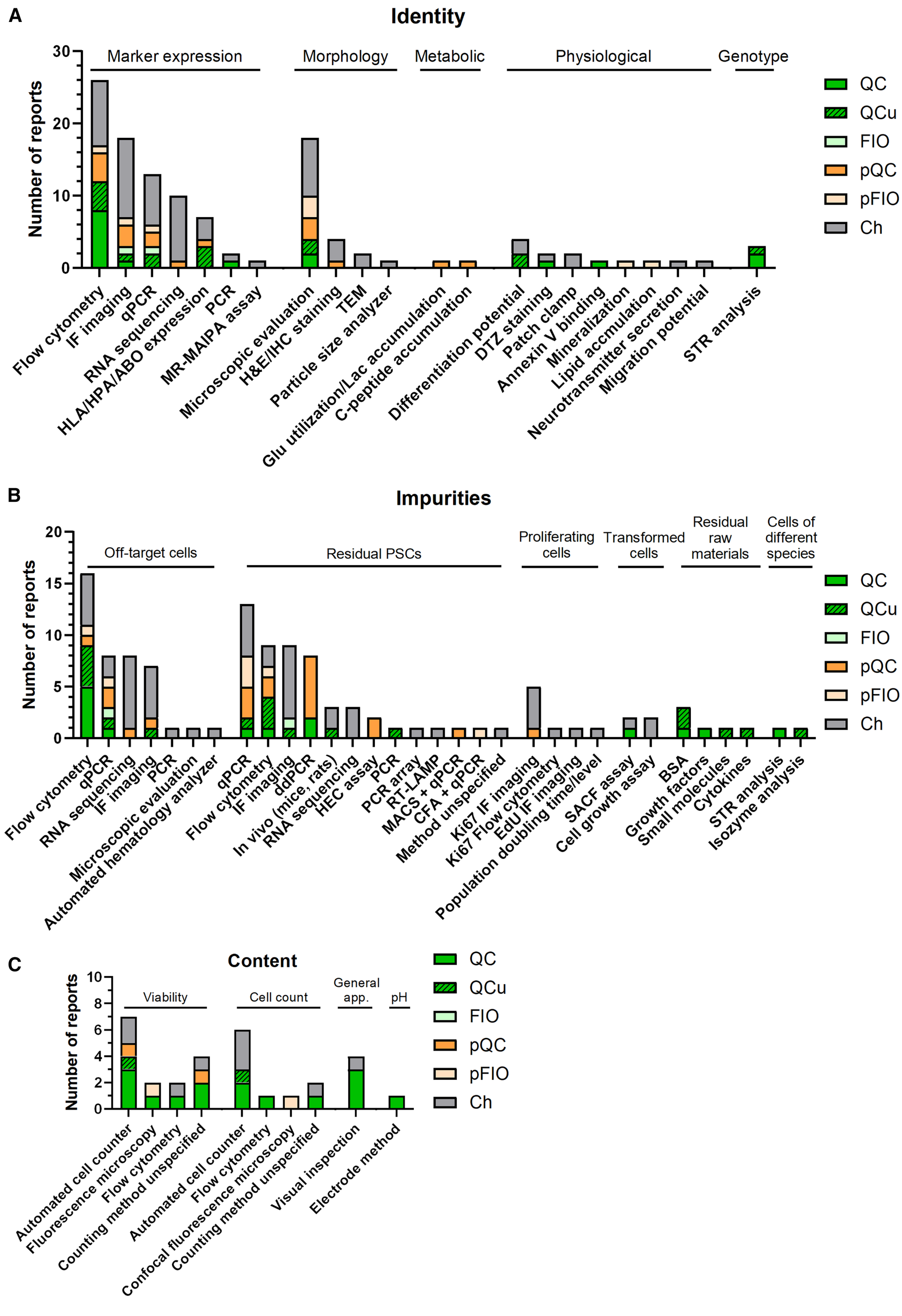

(legend on next page)

cells, tumorigenic cells, residual raw materials, and interspecies contamination (Figure 1B; Table S3).

The most frequently assessed impurity was off-target cells, often detected using the same techniques as described in the "Identity" section. Additionally, to detect off-target tissue in retinal organoids, the cells from other lineages arising during the differentiation process and residual progenitors were detected using single-cell PCR and bright-field microscopy (Watari et al., 2023). For a platelet product, an automated hematology analyzer was used to quantify the immature platelet fraction (Sugimoto et al., 2022b).

The absence of undifferentiated PSCs in any PSC-derived medicinal product is considered an important CQA, as these cells can have tumorigenic potential (Sato et al., 2019). Considerable attention was given to methods fit for the purpose of identifying residual undifferentiated PSCs, which could be present in low quantities among the product cells, requiring assays with sufficiently low detection thresholds. Although the most frequent techniques largely overlapped with the ones used for off-target cell identification, such as qPCR, flow cytometry, and IF imaging, we observed increasing use of novel techniques specifically for assessment of this CQA using droplet digital PCR (ddPCR) (Chung et al., 2022; Shi et al., 2022; Soma et al., 2024; Sun et al., 2024; Tsujimoto et al., 2022; Wang et al., 2024; Wu et al., 2023; Yasuda et al., 2024) and highly efficient culture (HEC) assay (Watanabe et al., 2023, 2024). Whilst the ddPCR is also based on marker detection, it is proposed to offer a lower detection limit (0.0001%–0.001%, as specified in the included reports) than the more widely used qPCR (0.001%–0.38%) and flow cytometry (0.002%–0.2%) (Figure S2; Table S4). The HEC assay, based on culturing a sample of the product in PSC maintenance medium to detect outgrowing PSC colonies, has also achieved a low limit of detection (0.0005-0.005%) (Watanabe et al., 2023, 2024). The detection threshold of an established qPCR technique could be improved through a combination of methods to enrich the residual PSC population by magnetic-activated cell sorting (MACS) (Tsujimoto et al., 2022) or pre-culturing cells in PSC maintenance medium (Sekine et al., 2023) before applying the detection method. *In vivo* mouse and rat studies have also been used to confirm the absence of tumorigenicity and to determine the percentage of undifferentiated PSCs by comparing the teratoma formation of PSC-derived end-products to spike-in samples with known PSC concentrations for up to 52 weeks (Park et al., 2024b; Wu et al., 2020; Yu et al., 2023) (Figure 1B). Interestingly, even though animal studies are rarely used as release tests due to long duration, high costs, and the need for repeated testing for every batch, a 6-week-long *in vivo* mouse study was listed together with other quality tests required by the Chinese regulatory authority for one of the products (Wu et al., 2020).

Potentially tumorigenic cells may also arise during the manufacturing process. Although some degree of expansion is expected in the end-products containing immature progenitor cells, which can either be the intended on-target cell population or off-target cells remaining due to incomplete differentiation, excessive proliferation represents a potential risk that could result in uncontrolled growth and tumor formation. Proliferative capacity was assessed by IF imaging or flow cytometry using the Ki67 marker (Bershteyn et al., 2023; Gantier et al., 2024; Izrael et al., 2018; Park et al., 2024b; Surendran et al., 2021; Yu et al., 2023) or EdU incorporation (Bershteyn et al., 2023) and by measuring population doubling time and expansion level (Park et al., 2024b). Dysregulated proliferative activity could also be an indication of malignant transformation as cells may accumulate mutations and become tumorigenic. The presence of such cells was assessed using a soft agar colony formation (SACF) assay, which is based on detecting the outgrowth of anchorage-independent colonies in agar over 2–3 weeks (Miyagawa et al., 2024; Wang et al., 2024). Similarly, proliferation rate was monitored in a cell

Figure 1. Identity, purity, and content evaluation of PSC-derived therapy products

(A) Target cell identity was confirmed by various techniques assessing marker expression, morphology, metabolic and physiological properties, and genotype. The assessment of the HLA, HPA, or ABO blood type antigens was performed by flow cytometry but was grouped as a separate method in identity testing due to the focus on immunophenotypic cell profile. See also Table S2.

(B) Techniques used to detect and/or quantify cellular and acellular impurities such as off-target cells; tumorigenic cells including residual PSCs, proliferating, and transformed cells; residual raw materials; and contaminating cells of different species. See also Figure S2; Tables S3 and S4.

(C) Content of the end formulation was evaluated based on cell viability and count, general appearance of cell suspension and sheets, and pH. The unspecified counting methods mostly relied on Trypan Blue exclusion and fluorescent staining. See also Table S5. BSA, bovine serum albumin; CFA, colony formation assay; ddPCR, droplet digital PCR; DTZ, dithizone; EdU, 5-ethynyl-2′-deoxyuridine; Glu, glucose; HEC: highly efficient culture; HLA, human leukocyte antigen; HPA, human platelet antigen; H&E, hematoxylin and eosin; IF, immunofluorescence; IHC, immunohistochemistry; Lac, lactate; MACS, magnetic-activated cell sorting; MR-MAIPA, modified rapid monoclonal antibody-specific immobilization of platelet antigens; RT-LAMP, reverse transcription loop-mediated isothermal amplification; SACF, soft agar colony formation; STR, short tandem repeat; TEM, transmission electron microscopy.

growth assay by culturing cells under regular passaging conditions in maturation medium for 5–6 passages up to 50 days (Miyagawa et al., 2024; Soma et al., 2024).

Acellular impurities originating from raw materials used during manufacturing and cryopreservation have been a part of the QC test panel in three reports (Sugimoto et al., 2022a; Wang et al., 2024; Wu et al., 2020). This included the detection of residual animal-derived components (bovine serum albumin), recombinant growth factors and cytokines, and small molecules (agonists, antagonists, inhibitors, antibiotics, and solvents) using methods such as ELISA, liquid chromatography-tandem mass spectrometry (LC-MSMS), and gas chromatography-mass spectrometry (GC-MS). Two reports tested for a possible contamination with cells of different species (Wang et al., 2024; Wu et al., 2020). The absence of interspecies contaminants was confirmed by STR analysis, simultaneously testing for the donor material identity, and by isozyme analysis relying on the electrophoretic mobility of enzyme isoforms, with human cells showing a distinct banding pattern (Chang et al., 2001).

To conclude, for the assessment of product purity as a CQA, different impurities were tested at varying frequencies, with off-target cells and residual PSCs being the most prevalent cellular impurities of interest. While flow cytometry, qPCR, and IF imaging remain the most frequently used techniques for QC, new methods are being developed and qualified, offering potentially superior detection thresholds. Culture-based methods can be suitable to detect tumorigenic cells of different origins.

Content

Product content, reflecting composition and therapeutic dose in the final formulation, was evaluated as a separate quality category. Viability and total cell count were the most frequently assessed CQAs, measured on single-cell samples using automated cell counters or flow cytometry (Figures 1C; Table S5). Alternatively, (confocal) fluorescence microscopy of cell viability and membrane integrity assays has been proposed and applied for whole aggregates (Kriedemann et al., 2024; Wang et al., 2024), with one of the reports describing a procedure to validate the imaging assay (Kriedemann et al., 2024). General appearance by visual inspection has also been set as a QC criterium for product release assessing the color and turbidity of the cell suspension ensuring absence of foreign particles (Kirkeby et al., 2023; Sugimoto et al., 2022a) and macroscopically observing the integrity of cell sheets after detaching from culture vessels (Miyagawa et al., 2022; Soma et al., 2024). In one platelet product, pH was included as a release parameter (Sugimoto et al., 2022a), as in platelet concentrates it can indicate metabolic activity and viability (FDA, 2008; Murphy and Gardner, 1975). Overall, total and viable cell numbers, supported by visual appearance and, in some products, pH, represent key content-related parameters for confirming the quality of PSC-derived cell therapies.

Safety

For the overview of quality testing related to safety, compendial sterility testing was excluded, and our analysis focused on the aspects specific to PSC-derived medicinal products such as genomic integrity, tumorigenicity, cytotoxicity, and adventitious virus presence.

Genomic integrity

Genomic integrity of the end products was assessed in 11 reports at different levels of detail (Bershteyn et al., 2023; Domingues et al., 2022; Kirkeby et al., 2023; Miyagawa et al., 2022, 2024; Neumayer et al., 2024; Park et al., 2024a; Shigyo et al., 2023; Shin et al., 2022; Soma et al., 2024; Wang et al., 2024). Chromosomal aberrations such as numerical variations or large structural abnormalities were evaluated by karyotyping 20-50 metaphases using Giemsa or multicolor fluorescence *in situ* hybridization (mFISH) staining (Domingues et al., 2022; Shigyo et al., 2023; Shin et al., 2022; Soma et al., 2024) (Figure 2A; Table S6). For the assessment of post-mitotic cells and a more detailed analysis of mutations in known high-risk genomic locations and structural variants associated with cancer or other diseases, several targeted approaches were employed. Copy number variations (CNVs) were identified by a comparative genomic hybridization (CGH) array (Miyagawa et al., 2022), while both CNVs and copy neutral loss of heterozygosity (CN-LOH) events could be detected by single nucleotide polymorphism (SNP) arrays (Bershteyn et al., 2023; Kirkeby et al., 2023; Miyagawa et al., 2024; Soma et al., 2024) (e.g., HumanOmniExpress-24 (Miyagawa et al., 2024; Soma et al., 2024) and HumanCytoSNP-12 (Kirkeby et al., 2023)), and a chromosomal microarray (CytoScanHD (Park et al., 2024a)).

High-resolution next-generation sequencing (NGS) methods, capable of detecting single-base changes, were the most frequently used techniques for PSC-derived end-products, aiming to confirm the absence of (malignant) *de novo* mutations acquired during cell culture. Targeted sequencing was utilized in four reports using different cancer gene panels, covering a single gene (*TP53*) or up to 344 cancer-related genes (Bershteyn et al., 2023; Kirkeby et al., 2023; Neumayer et al., 2024; Park et al., 2024a) (Table S7). Alternatively, untargeted whole-genome sequencing (WGS) or whole-exome sequencing (WES) was done to acquire comprehensive information on CNVs, single-nucleotide variants (SNVs), insertions and deletions (INDELs). The sequencing depth reached approximately 40–83× for WGS (Miyagawa et al., 2024; Neumayer et al., 2024; Soma et al., 2024; Wang et al.,

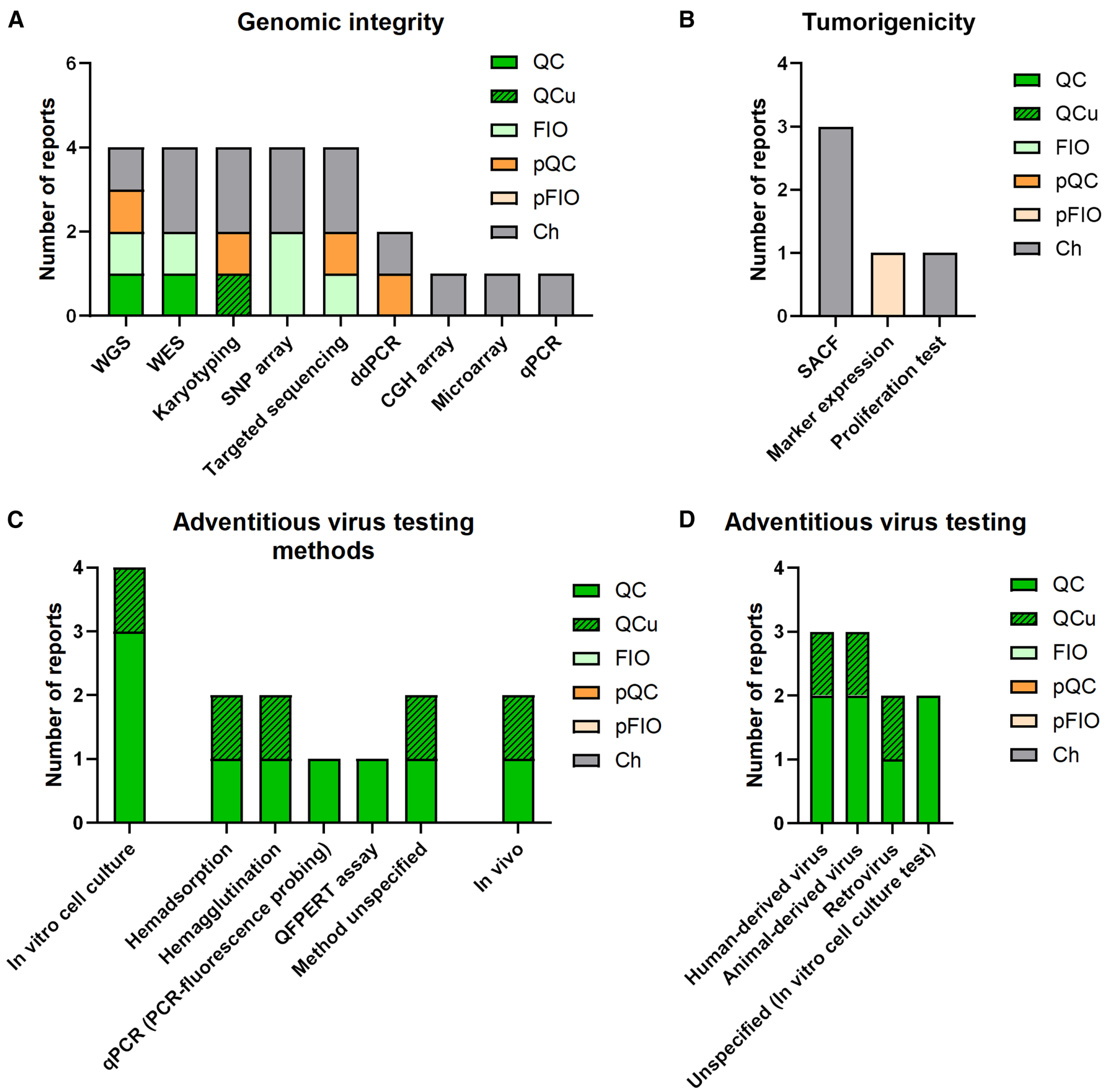

Figure 2. Safety testing of PSC-derived therapy products
(A) Methods used to assess genomic integrity in the end-products. See also Table S7.
(B) *In vitro* tests to determine the tumorigenic potential of product cells.
(C) Methods for detecting adventitious viruses based on the use of detector cell lines, cell-free *in vitro* techniques, and *in vivo* studies. See also Figure S3A.
(D) Virus types and species targeted for detection in adventitious virus testing. See also Figure S3B. For the whole figure, see also Table S6. CGH, comparative genomic hybridization; ddPCR, droplet digital PCR; QFPERT, quantitative fluorescent product-enhanced reverse transcriptase; SACF, soft agar colony formation; SNP, single nucleotide polymorphism; WES, whole-exome sequencing; WGS, whole-genome sequencing.

2024), 83–300× for WES (Miyagawa et al., 2024; Park et al., 2024a; Soma et al., 2024; Wang et al., 2024), and 100–300× for targeted sequencing (Bershteyn et al., 2023; Park et al., 2024a) (Table S7).

Results acquired by the different NGS and array methods were often analyzed by collating the identified variants to publicly available databases reporting on genetic variations related to tumorigenicity and other pathological conditions (Miyagawa et al., 2024; Park et al., 2024a; Soma et al., 2024; Wang et al., 2024). In addition, databases containing unannotated and benign genomic variants were cross-referenced to exclude constitutive variants unlikely to be related to disease (Kirkeby et al., 2023; Miyagawa et al., 2024; Neumayer et al., 2024). Moreover, various *in silico* tools were used to predict the deleterious nature of the captured variants (Park et al., 2024a) by evaluating the

impact on the change in protein structure (Montenegro et al., 2021). Follow-up analysis using targeted ddPCR has been proposed as a QC test (Park et al., 2024a) and was used to detect mutated variants present at low frequencies previously identified through NGS (Neumayer et al., 2024; Park et al., 2024a), while qPCR was shown to be more prone to false-positive results (Park et al., 2024a) (Figure 2A).

Based on the used targeted panels and databases, the primary goal of assessing genomic integrity in the final PSC-derived medicinal products was to detect cancer-related variants and evaluate potential tumorigenicity of transformed cells. While karyotyping was frequently used where possible, high-resolution NGS and array-based techniques suitable for assessing genomic aberrations in post-mitotic PSC-derived cells are gaining popularity as QC and FIO tests.

In vitro *tumorigenicity testing*

Some methods previously described in the "Impurities" section were applied with the primary goal to evaluate the potential to form tumors rather than estimating levels of specific cellular impurities, and were therefore assigned to the "Safety" category (Gantier et al., 2024; Miyagawa et al., 2024; Sugimoto et al., 2022a, 2022b; Wu et al., 2020). Quantification of proliferating cell colonies was done in agarose (SACF assay) (Gantier et al., 2024; Sugimoto et al., 2022a; Wu et al., 2020), suspension culture (Sugimoto et al., 2022b), and adherent culture assays (Sugimoto et al., 2022b) for up to 21 days (Wu et al., 2020), 22 days (Sugimoto et al., 2022b), and 12 days (Sugimoto et al., 2022b), respectively (Figures 2B; Table S6). Master Cell Bank PSCs (Sugimoto et al., 2022a; Wu et al., 2020) and intermediate product cells (Sugimoto et al., 2022a, 2022b; Wu et al., 2020) served as comparators, with cancer cell lines (Sugimoto et al., 2022a, 2022b) or cancer cell spike-ins (Sugimoto et al., 2022b) as positive controls. One report proposed quantifying LIN28A marker expression levels as an alternative to *in vivo* tumorigenicity testing, as this marker had been found to correlate with tumor formation frequency in mice (Miyagawa et al., 2024).

In vitro *toxicity testing*

Two reports described the use of *in vitro* cell viability models to investigate potential cytotoxic effects of the product on surrounding healthy cells (Cichocki et al., 2022; Domingues et al., 2022). For genetically engineered NK cells, absence of off-target cytotoxicity was assessed by co-culturing the product with pre-labeled hematopoietic progenitor cells followed by viability assessment using flow cytometry (Cichocki et al., 2022). For engineered skin tissue, the toxicity of reconstituted composite keratinocyte-fibroblast skin grafts was assessed by applying the product's conditioned medium to intact primary keratinocyte monolayers and recording apoptosis via live cell imaging (Domingues et al., 2022).

Adventitious virus testing

Adventitious viral agent testing, performed to detect viral contamination potentially present in the starting material or introduced through raw materials during manufacturing, was described in only five products, but applied as a QC release test in all these cases (Kirkeby et al., 2023; Park et al., 2024b; Wang et al., 2024; Wu et al., 2020, 2024) (Figures 2C and 2D; Table S6). Most reports described a combination of methods used to assess viral safety in the PSC-derived end products (Figure S3). *In vitro* cell culture assays were most frequently described, using co-cultures of cell product (or spent culture media) with indicator cell lines of human, monkey, bovine, and porcine origin (Kirkeby et al., 2023; Park et al., 2024b; Wang et al., 2024; Wu et al., 2020) to observe cytopathic effect (Kirkeby et al., 2023; Wang et al., 2024). Hemadsorption and hemagglutination tests were used independently or as follow-up methods for the *in vitro* cell culture tests (Wang et al., 2024; Wu et al., 2020). For the detection of known viral contaminants and retroviruses, qPCR (Wang et al., 2024) and quantitative fluorescent product-enhanced reverse transcriptase (QFPERT) assay (Kirkeby et al., 2023) were used, respectively. *In vivo* testing was performed in two reports, using various animal models (Wang et al., 2024; Wu et al., 2020).

All in all, the safety of the PSC-derived medicinal products was evaluated with regards to genomic integrity, tumorigenic outgrowth, cytotoxicity, and infectious viral agents. A number of *in vitro* approaches were used to determine the product safety profile and possible pathogenic, toxic, and infectious risks.

Potency

Due to the variety of medicinal products that can be obtained from PSCs, approaches to examine their potency can be tailored to different characteristics and differ per product. Some of the potency tests aim to directly measure CQAs linked to the mechanism of action, while others rely on surrogate markers. However, distinguishing between direct or surrogate potency assays proved challenging due to the highly product- and indication-specific context defining the therapeutic mechanism of a PSC-derived medicinal product. Therefore, it was not possible to consistently and reliably classify this based on the limited information provided about potency development in the included reports. For these reasons, potency tests designed to evaluate the biological activity of a product were grouped based on the assessed characteristics rather than by method used and represent a combination of both direct and surrogate assays (Figure 3; Table S8).

The most frequent approach to evaluate product potency was measuring production or secretion of cell-specific hormones and cytokines (Cichocki et al., 2022; Gantier et al.,

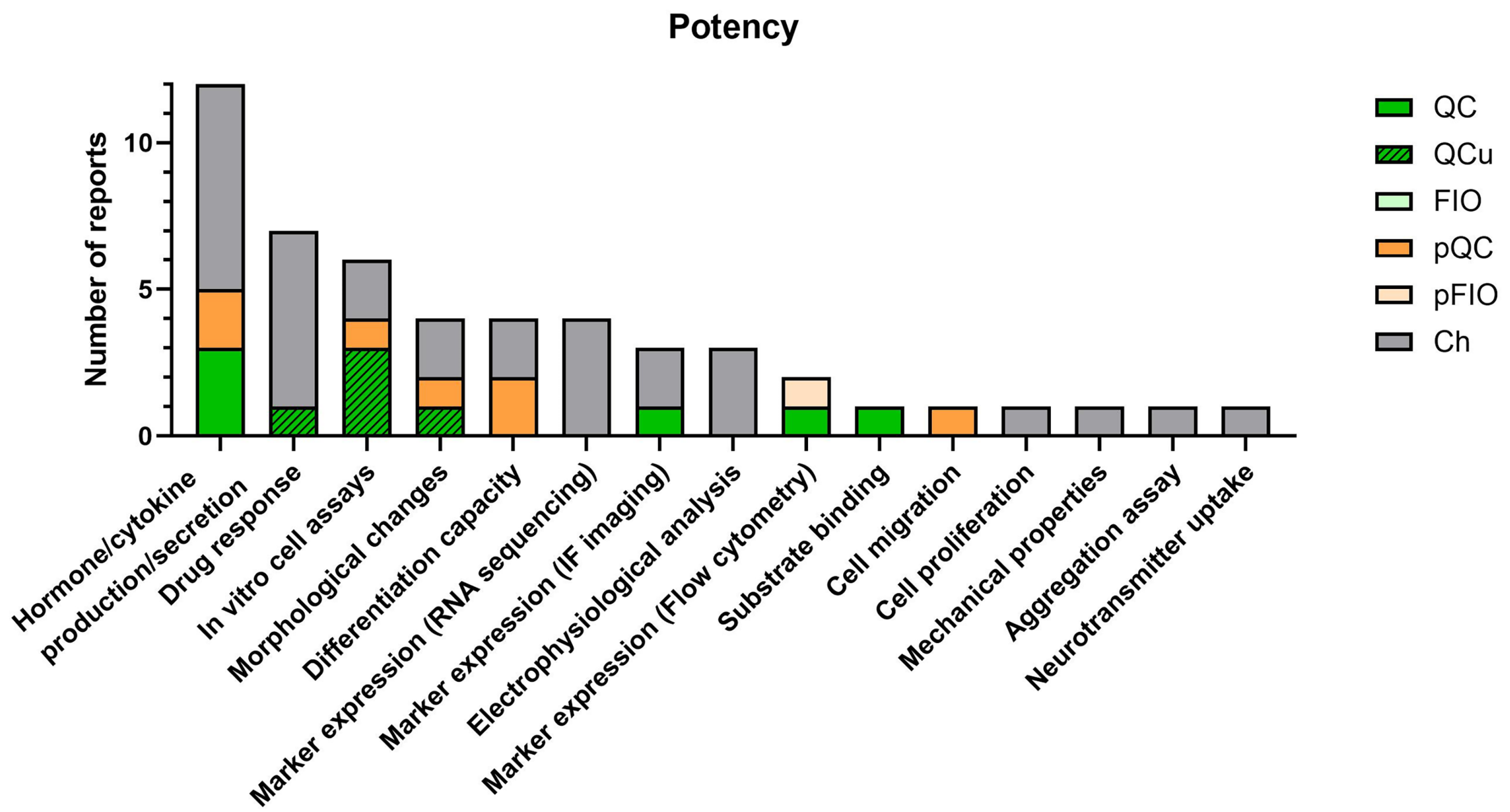

Figure 3. Potency tests of PSC-derived therapy products
Functionality of the cells and tissue constructs was evaluated using a range of direct and surrogate assays based on various physiological properties, *in vitro* cell assays, and marker expression. See also Table S8. IF, immunofluorescence.

2024; Gotkine et al., 2023; Huang et al., 2023; Iworima et al., 2024; Miyagawa et al., 2024; Naderi et al., 2023; Park et al., 2024b; Surendran et al., 2022; Wang et al., 2024; Wu et al., 2020, 2024). Signature molecules, such as cell-specific growth factors (Gantier et al., 2024; Gotkine et al., 2023; Miyagawa et al., 2024; Surendran et al., 2022), glucagon and insulin or its by-product C-peptide (Iworima et al., 2024; Wang et al., 2024; Wu et al., 2024), neurotransmitters (Naderi et al., 2023; Park et al., 2024b), and immunomodulatory cytokines (Cichocki et al., 2022; Huang et al., 2023; Wu et al., 2020), were detected using ELISA (Gantier et al., 2024; Gotkine et al., 2023; Huang et al., 2023; Iworima et al., 2024; Naderi et al., 2023; Park et al., 2024b; Surendran et al., 2022; Wang et al., 2024; Wu et al., 2020, 2024), radioimmunoassay (Iworima et al., 2024), mass spectrometry (Gantier et al., 2024), electrochemiluminescence (Cichocki et al., 2022), or bead-coupled multiplex immunoassays (Miyagawa et al., 2024). The release of the molecules was often assessed in response to a biologically relevant inducing factor, such as membrane depolarization by ion (Naderi et al., 2023; Park et al., 2024b) or glucose stimulation (Iworima et al., 2024; Wang et al., 2024; Wu et al., 2024), treatment with pro-inflammatory cytokines (Wu et al., 2020) or synovial fluid from meniscus injury patients (Huang et al., 2023), hypoxia induction (Miyagawa et al., 2024), or co-culture with a tumor cell line (Cichocki et al., 2022). In addition, hormone release was assessed to evaluate cell polarity by measuring the differential growth factor secretion in supernatant on the apical and basal side of the retinal pigment epithelium cell layer (Surendran et al., 2022). In rare cases, the secreted proteins were also assessed for their activity and correct isoform (Wu et al., 2020).

In vitro cell assays utilized model cells co-cultured with the medicinal product or its conditioned medium to assess the activity of the product. Stimulation of tissue regeneration was evaluated through chondrocyte migration (Huang et al., 2023) and keratinocyte scratch assay (Domingues et al., 2022). Immunomodulatory effects were observed by inhibited lymphocyte proliferation (Huang et al., 2023; Wu et al., 2020) and anti-fibrotic effects by reduced myofibroblast *trans*-differentiation (Wu et al., 2020). CAR-NK cell potency was measured by antigen-specific cytotoxicity against engineered cancer cells and persistence via serial restimulation (Cichocki et al., 2022). Astrocyte functionality was demonstrated by promoting axonal outgrowth and protecting neurons from oxidative stress (Izrael et al., 2018). Angiogenic interactions between MSCs and endothelial cells were assessed using a dye transfer assay (Shin et al., 2022).

Other CQAs related to potency that were interrogated included morphological changes and structural organization

of cells (Domingues et al., 2022; Huang et al., 2023; Shin et al., 2022; Wu et al., 2020), differentiation capacity (Domingues et al., 2022; Naderi et al., 2023; Shigyo et al., 2023; Zhao and Liu, 2022), electrophysiological analyses (Miyagawa et al., 2024; Naderi et al., 2023; Yu et al., 2023), PAC-1 binding and aggregation for platelets (Sugimoto et al., 2022a), MSC migration toward tumor cells (Zhao and Liu, 2022), corneal epithelial cell proliferation (Soma et al., 2024), mechanical strength of cardiomyocyte patches (Miyagawa et al., 2024), and neurotransmitter uptake by astrocytes (Izrael et al., 2018). Drug response was often integrated in other assays like hormone secretion or electrophysiological analysis to assess cell activation or pathway specificity by applying stimulating compounds and challenging the cells with inhibitors (Iworima et al., 2024; Izrael et al., 2018; Kobayashi et al., 2024; Miyagawa et al., 2024; Naderi et al., 2023; Shin et al., 2022; Yu et al., 2023).

Potency evaluation by marker expression was assessed through bulk and single-cell RNA sequencing (Huang et al., 2023; Neumayer et al., 2024; Wu et al., 2020; Yu et al., 2023), IF imaging (Neumayer et al., 2024; Soma et al., 2024; Surendran et al., 2022), and flow cytometry (Neumayer et al., 2024; Sugimoto et al., 2022a). These methods were rarely used as stand-alone tests and required additional steps to validate their suitability by demonstrating a correlation with true biological activity (Neumayer et al., 2024). In addition, PSC-derived cell potency was often compared to primary cells or cell lines of the same lineage to demonstrate functional equivalence.

In conclusion, the potency of PSC-derived products can be evaluated in a multitude of ways using product-specific parameters, with hormone and cytokine production being the most applied approach for measuring biological activity. Suitable assays need to be determined by the product developers in consultation with the regulatory authorities. Surrogate methods correlating with biological activity can be validated when justified—usually when measuring direct potency as a QC release test is not feasible. Careful case-by-case consideration is required for designing QC strategies and evaluation of the desired quality product profile, which is highly related to the clinical indication for which the product is developed.

Stability

Cell product attributes related to identity, impurities, content, genomic integrity, and potency have been tested in stability studies to evaluate the persistence of these CQAs. This included the impact of prolonged culture; in-use stability during room temperature preservation, storage in the formulation solution, and transportation to the clinical site; long-term cryopreservation; and delivery through medical devices, where applicable (Figure S4; Table S9).

The dominant CQA assessed across all categories of the stability studies was cell viability (Gantier et al., 2024; Kirkeby et al., 2023; Kobayashi et al., 2024; Naderi et al., 2023; Watari et al., 2023; Wu et al., 2020; Yu et al., 2023), succeeded by marker expression evaluating identity and purity of the product using flow cytometry, qPCR, and IF imaging of on- and off-target cells and absence of pluripotency markers (Gantier et al., 2024; Kirkeby et al., 2023; Kobayashi et al., 2024; Naderi et al., 2023; Sugimoto et al., 2022b; Watari et al., 2023; Yu et al., 2023). Cell morphology was the third most common attribute often assessed by bright-field (Gantier et al., 2024) or IF imaging (Watari et al., 2023; Yu et al., 2023) of replated cells or multilayered structures (Sugimoto et al., 2022a). Specifications for cell count, general appearance of the suspension, and pH were defined in a shipping test critical for the release of a platelet product after transportation to the clinical care facility (Sugimoto et al., 2022a). Simulation of storing the cell preparation in a formulation buffer at 4°C or on ice prior to the transplantation or during transportation was assessed by inspecting cell viability for up to 4-72 h (Kobayashi et al., 2024; Naderi et al., 2023; Wu et al., 2020; Yu et al., 2023). Genomic stability was assessed by karyotyping (Naderi et al., 2023) or by an SNP array (Kirkeby et al., 2023), which was classified as an FIO test for clinical release (Figure 2A) and compared CNV and CN-LOH events between directly thawed cells and after 14 days of additional culture. Prolonged *in vitro* culture of 4–12 weeks was also applied for cardiac spheroids to monitor their viability, nourishment of the core structure, and cellular purity over extended periods (Kobayashi et al., 2024) (Figure S4). Furthermore, in-use and cryopreservation stability was evaluated by assessing potency through marker expression (Sugimoto et al., 2022a), cytokine secretion (Surendran et al., 2021), and replating ability to demonstrate functional adhesive properties (Gantier et al., 2024). The longest time-points for stability testing after cryopreservation were reported at 6 months (Naderi et al., 2023; Yu et al., 2023) and at 36 months (Gantier et al., 2024; Kirkeby et al., 2023) with a planned extension to a total of 60 months (Kirkeby et al., 2023). The impact of a delivery device was monitored immediately after extruding the cells (Kirkeby et al., 2023; Naderi et al., 2023; Yu et al., 2023) and up to 11 months post-transplantation in a porcine *in vivo* study with a single animal to observe the survival and maturation of the grafted cells (Kirkeby et al., 2023).

In conclusion, stability studies mostly focused on detecting changes in cell viability and product composition, evaluated by marker expression. The handling conditions reflected the period after manufacturing and prior to patient transplantation, which included storage, transportation, final formulation, and administration through a

delivery device. These items represent critical steps in product handling that could affect the product quality.

Non-clinical *in vivo* studies

Non-clinical animal studies remain an essential part of safety and efficacy evaluation during the development of medicinal products, including PSC-derived therapies. A number of parameters were extracted from the included reports regarding the design of the *in vivo* studies to identify the current practices in biodistribution, tumorigenicity, toxicity, and efficacy assessment (Table S10-S13).

Animal models

Key aspects of animal models, such as species, immunosuppression and disease state, number of animals used, sex, and follow-up duration, were extracted from the included reports containing *in vivo* testing (Bershteyn et al., 2023; Cichocki et al., 2022; Corsini and Mitola, 2023; Gantier et al., 2024; Huang et al., 2023; Izrael et al., 2018; Kirkeby et al., 2023; Kobayashi et al., 2024; Luciani et al., 2022; Manley et al., 2017; Miyagawa et al., 2022, 2024; Naderi et al., 2023; Neumayer et al., 2024; Oh et al., 2022; Park et al., 2024b; Rong et al., 2024; Shigyo et al., 2023; Shin et al., 2022; Soma et al., 2024; Sugimoto et al., 2022a, 2022b; Surendran et al., 2021; Wang et al., 2024; Watari et al., 2023; Wu et al., 2020, 2024; Yu et al., 2023) (Figures 4, S5, and S6).

Mice were the predominant species used for all studies, closely followed by rats (Figure 4A). For most safety and efficacy studies in large animal models, non-human primates and rabbits were the model of choice, while pigs were the preferred model to test the efficacy of specific products like cardiomyocyte patch (Miyagawa et al., 2024). To assess teratoma formation caused by ESCs, a chick embryo model was proposed, offering a growth-stimulating highly-vascularized environment and allowing to monitor the teratoma formation through a transparent window (Corsini and Mitola, 2023).

Most of the safety studies were conducted in immunocompromised murine strains, while large animal models were genetically unaltered (Figure 4A). In the majority of the reviewed reports, drug-based immunosuppression was not used or remained unreported (Figure S5A), which could be attributed to the preferred use of the genetically induced immunodeficient mice and rats. However, when immunosuppressant use was reported, it was administered to both immunocompetent and immunodeficient animals. Immunosuppressive drugs in animal models can not only be administered to prevent rejection of a xenograft but also to mimic clinical conditions where immunosuppressive treatment is applied as an adjuvant. Meanwhile, efficacy was primarily evaluated in disease models induced genetically, mechanically, or via drug or toxin treatment (Figure 4A). In some cases, studies were performed in healthy animals in addition to diseased mice (Bershteyn et al., 2023; Wang et al., 2024) and non-human primates (Kobayashi et al., 2024).

The number of animals varied per study, not only between but also within the groups of the same species (Figures S6A and S6B). Based on the available reported data, the total number of animals, including all dose groups, controls, and spares (Figure S6A), as well as the lowest animal number used to test a single dose of a product (Figure S6B) generally ranged from a few up to a cohort of hundreds for mice and rats. In studies with other species, considerably lower animal numbers were reported, reaching up to a few dozen for a minimum cohort used to test a single dose of a product (Figure S6B) and for complete studies (Figure S6A).

Where sex was specified, a study set-up using a single sex or a mixed population was reported at a similar rate for biodistribution, tumorigenicity, and efficacy assessment (Figure S5B). For toxicity studies, a mixed population was more often chosen. One report mentioned a limited possibility to include both male and female pigs (Miyagawa et al., 2024), while another report justified their choice for female rats in tumorigenicity evaluation of retinal grafts as necessary to limit aggressive behavior among co-housed animals (Watari et al., 2023).

The longest follow-up duration was generally observed for mice and rats, varying from 7 days studying biodistribution of platelets (Sugimoto et al., 2022a) and efficacy of MSCs (Shin et al., 2022) to 1.5 years monitoring efficacy of interneurons (Bershteyn et al., 2023) and tumorigenicity of retinal sheets (Watari et al., 2023) (Figure S6C). Non-human primates were followed for a maximum of 4 weeks in an efficacy study of islets (Wu et al., 2024) to 36 weeks in a long-term toxicity study of repeatedly dosed immunity-and-matrix-regulatory cells (IMRCs) (Huang et al., 2023). Rabbits were used to follow the biodistribution of platelets for up to 24 h (Sugimoto et al., 2022a) and toxicity and efficacy of IMRCs in meniscus regeneration for 8 weeks (Huang et al., 2023), while cardiac regeneration in pigs was observed for 12 weeks (Miyagawa et al., 2024). Teratoma detection in the proposed chick embryo model could be achieved in a rapid turnaround time of 10 days (Corsini and Mitola, 2023).

Dosing studies, controls, and site of administration

An essential part of a testing strategy for a new medicinal product is deciding on the dosing. Most of the *in vivo* studies assessed the effect of a single dose of cells (Figure 4B). The selected dose was often related to the estimated cell number required for efficacy or a maximal feasible dose limited by the volume constraints to increase the possibility of detecting toxic effects, tumorigenesis, or ectopic distribution. Dose-escalation of 2–5 different cell amounts was primarily utilized in finding a therapeutically effective or non-toxic dose. Furthermore, repeated dosing

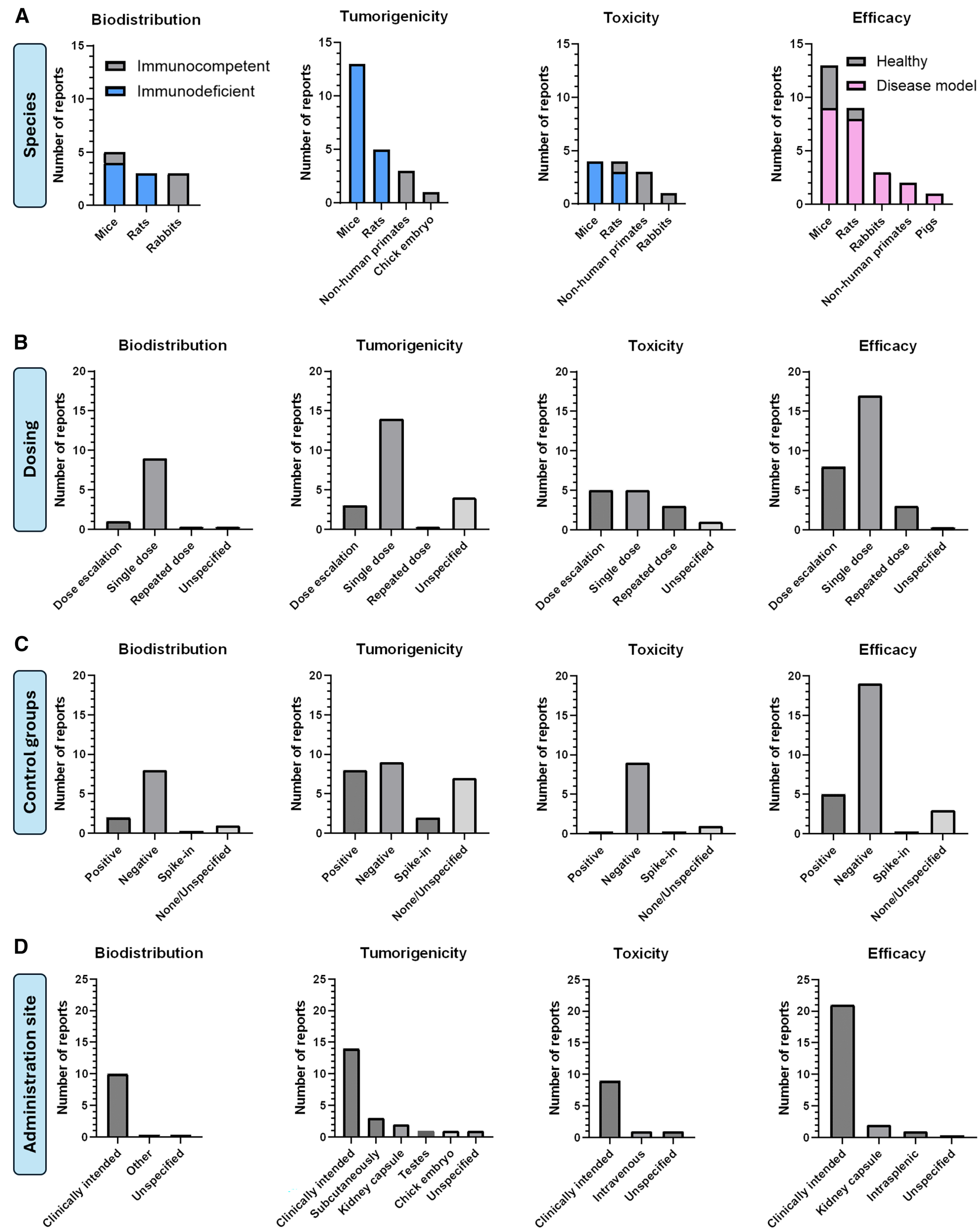

Figure 4. Animal species and product dosing in non-clinical studies of PSC-derived therapy products
(A) Animal species and employment of immunosuppression or disease models in non-clinical safety and efficacy assessments described in the included reports.
(B) Number of different product doses tested for PSC-derived products in animal studies.
(C) Inclusion of control groups in the non-clinical studies.
(D) Sites of administration through clinically intended or other routes utilized in the analyzed *in vivo* studies. See also Figures S5 and S6; Tables S10–S13.

was used to assess toxicity and efficacy, with the most extensive example of IMRCs administered intravenously up to 22 times within 24 weeks in non-human primates (Huang et al., 2023; Wu et al., 2020).

In addition to the animal groups dosed with the cell product, negative control groups were often used for comparison (Figure 4C). Negative controls either remained untreated (Cichocki et al., 2022; Gantier et al., 2024; Naderi et al., 2023; Rong et al., 2024; Shin et al., 2022; Wang et al., 2024; Watari et al., 2023), were sham-operated (Miyagawa et al., 2024; Shin et al., 2022; Watari et al., 2023; Wu et al., 2024), or were dosed with the vehicle (saline, medium, or formulation buffer) (Bershteyn et al., 2023; Huang et al., 2023; Izrael et al., 2018; Kirkeby et al., 2023; Kobayashi et al., 2024; Manley et al., 2017; Naderi et al., 2023; Park et al., 2024b; Rong et al., 2024; Sugimoto et al., 2022a, 2022b; Surendran et al., 2021; Wu et al., 2020; Yu et al., 2023); Matrigel for tumorigenicity studies (Soma et al., 2024; Wang et al., 2024); carrier cells to maintain cell density (Kirkeby et al., 2023); dispersed cells compared to spheroids (Kobayashi et al., 2024); gene-engineered cells containing the backbone construct (Cichocki et al., 2022); or intermediate cell product (Sugimoto et al., 2022a; Wu et al., 2024). Positive controls were mainly used in tumorigenicity studies by administering cancer cell lines (Oh et al., 2022; Park et al., 2024b; Sugimoto et al., 2022a), PSCs (Kirkeby et al., 2023; Neumayer et al., 2024; Soma et al., 2024; Wang et al., 2024; Wu et al., 2024; Yu et al., 2023), or unpurified product (Miyagawa et al., 2024). Spike-in controls with PSCs (Park et al., 2024b) or patient-derived induced carcinoma cells (Neumayer et al., 2024) mixed into the product or differentiated cells enabled titrating the number of cells able to initiate tumor growth. Controls consisting of intermediate cell product (Sugimoto et al., 2022a) or PSCs (Neumayer et al., 2024) in biodistribution studies were used to characterize the general cell dissemination or possible cancer metastases. Some efficacy studies compared the cell therapy product to approved chemical drugs (Cichocki et al., 2022; Shin et al., 2022; Wu et al., 2020), primary cells (Cichocki et al., 2022; Gantier et al., 2024; Neumayer et al., 2024; Wu et al., 2020), or equivalent PSC-derived research-grade cells (Shin et al., 2022).

Site of administration may be a key determinant for cell efficacy, tumor-stimulating environment, and routes of dissemination. Subcutaneous injection in a murine model has been described as a gold standard for studying teratoma formation (Corsini and Mitola, 2023) and was utilized for detecting possible tumor growth by transplanting corneal endothelial and epithelial cells (Soma et al., 2024; Yu et al., 2023) (Figure 4D). However, across all *in vivo* studies, cell products were predominantly administered to the clinically intended site, including the brain, spinal cord, heart, vein, eye, skin, joint, muscle sheath, and bladder. Sometimes, other sites (testes (Wu et al., 2020)) and model systems (chick embryos; Corsini and Mitola, 2023) were preferred, possibly for their hypoimmunogenic environment. Islet cells were transplanted under the kidney capsule in mice to evaluate their potential tumorigenicity and functionality (Wang et al., 2024; Wu et al., 2024). Toxicity of IMRCs intended for meniscus injury was tested by intravenous infusion (Huang et al., 2023), while intrasplenic injection of hepatic progenitor cells facilitated the delivery of cells to the liver due to the limited access to the portal vein in mice (Gantier et al., 2024).

GLP conditions

Good Laboratory Practice (GLP) is an established quality system for conducting non-clinical safety studies, ensuring appropriate planning, performance, monitoring, recording, archiving, and reporting (FDA, 2025; The European Parliament and the Council of the European Union, 2004). GLP conditions are usually applied for pivotal studies designed to obtain confirmatory data on product safety for submission to the regulatory authorities. Among the reported animal studies, the majority did not mention the application of GLP conditions (Figure S5C). Biodistribution and toxicity studies were most frequently performed under GLP conditions (4/10) (Izrael et al., 2018; Kirkeby et al., 2023; Manley et al., 2017; Park et al., 2024b), followed by tumorigenicity studies (6/20) (Izrael et al., 2018; Kirkeby et al., 2023; Manley et al., 2017; Oh et al., 2022; Park et al., 2024b; Shin et al., 2022). GLP conditions were rarely applied for efficacy studies (1/23). Most studies following GLP regulations were outsourced to third parties (Izrael et al., 2018; Kirkeby et al., 2023; Manley et al., 2017; Park et al., 2024b; Yu et al., 2023), while some were performed within the manufacturer's own facilities (Oh et al., 2022; Shin et al., 2022) (Tables S10–S13). Conversely, some non-GLP studies were transferred to external facilities (Wu et al., 2020, 2024).

Use of in vivo *studies for QC testing*

Interestingly, while most non-clinical *in vivo* studies were performed during the development phase of the product, some *in vivo* tumorigenicity assays were included as QC release tests (Miyagawa et al., 2022; Wang et al., 2024). Allogeneic cryopreserved iPSC-derived cardiomyocytes intended for use in a clinical trial were first transplanted as a patch onto the hearts of 10 immunodeficient mice, and the product was released after no tumor formation was observed at the 16-week endpoint (Miyagawa et al., 2022). Another report specified a similar 4-month study with 50 immunodeficient mice as a QC release test of an autologous cryopreserved islet product transplanted under the kidney capsule, using Matrigel with ascitic fluid and the iPSC Master Cell Bank starting material as negative and positive controls, respectively (Wang et al., 2024). For

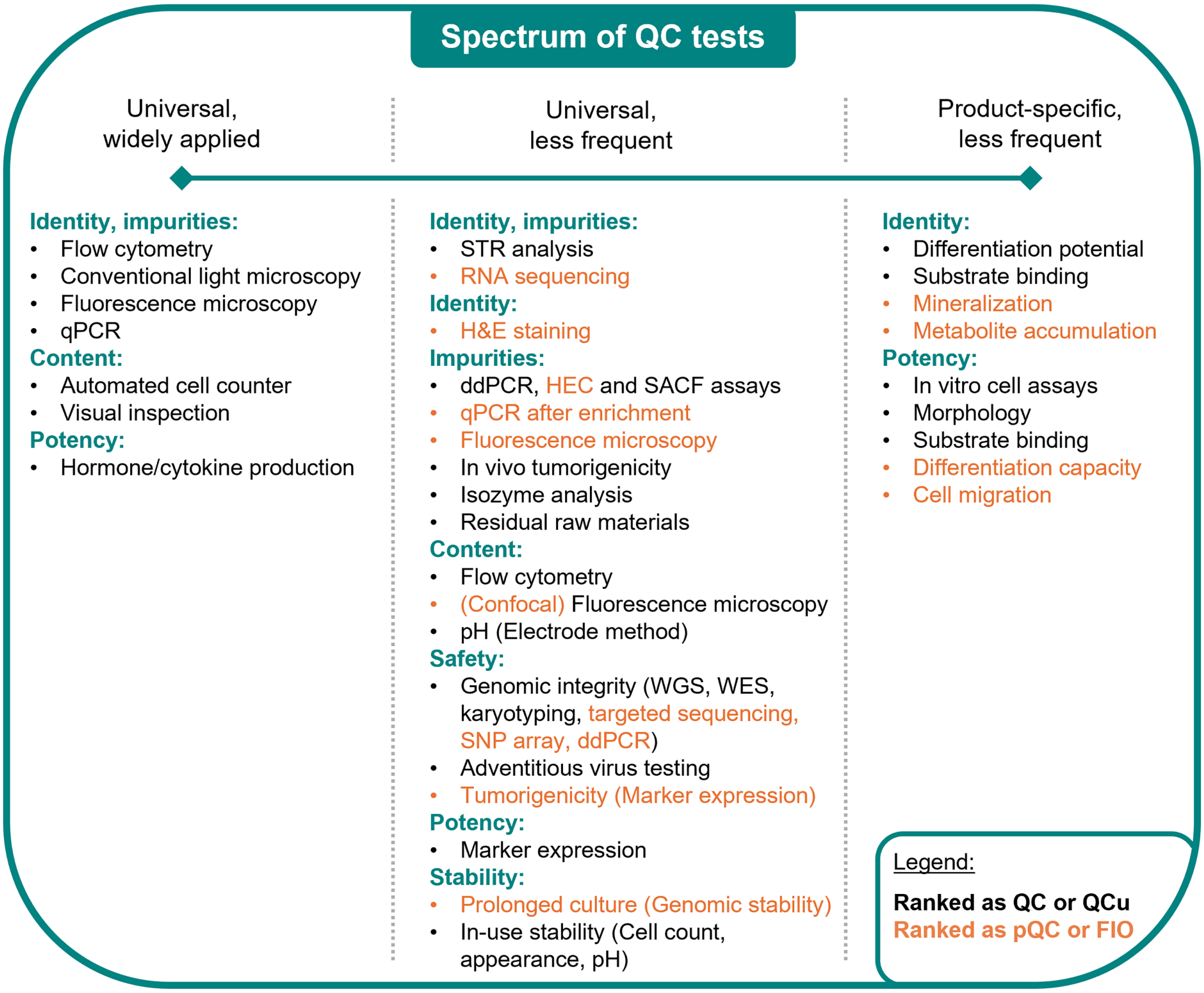

Figure 5. Spectrum of QC tests on the scale of universality and frequency of application
Methods on the left side can be utilized universally for multiple PSC-derived cell lineages and were frequently reported for QC release testing. Methods on the right side focus on product-specific properties mostly related to cell functionality and were used less frequently compared with other techniques within the same groups of tests. Methods in the middle of the spectrum represent potentially widely applicable methodologies which were less frequently reported. Methods in black were ranked as QC or QCu, while methods in orange obtained pQC or FIO as the highest applied ranks.

cryopreserved allogeneic (Miyagawa et al., 2022; Wu et al., 2020) and autologous (Wang et al., 2024) products released as one batch, the possibility of long-term preservation contributed to the feasible implementation of a release test with a substantial lead time.

Typical profile of non-clinical studies and quality testing

From the available information, it is evident that the outcomes on efficacy and tumorigenicity of PSC-derived cell therapy products were reported more frequently than biodistribution and toxicity (Figure 4A). The typical profile of non-clinical safety and efficacy studies delineated a cohort nearing a hundred immunosuppressed rodents with a balanced male/female ratio, comparing the effect of a single dose of the cell product to a negative control, administered via a clinically relevant route and followed up for a median of 5–10 months.

The detailed systematic data extraction of the quality testing strategies enabled us to compile a list of CQAs and applied methods for QC release testing of PSC-derived medicinal products for clinical application (Table S14). These QC tests ranged over a spectrum of universality and application frequency (Figure 5). Some techniques can be universally applied for multiple PSC-derived cell lineages and were widely implemented. The other end of the spectrum hosts tests that were relatively less frequently observed within the corresponding testing groups and analyzed product-specific properties, mostly dependent on cell function and mechanism of action. Meanwhile, methodologies clustering in the middle of the spectrum can be universal across PSC-derived medicinal products, however, they

were reported at a lower frequency. These methods included both approved QC tests as well as proposed, emerging, and potentially informative methods ranked as pQC and FIO. In addition, we provide a product-specific overview of the performed QC release tests per product type and the available specifications or reported results, which could benefit therapy developers interested in comparing the methods and acceptance criteria applied to similar products (Table S15). This resulted in a comprehensive resource providing information on testing strategies that have been used to qualify cell and tissue-engineered products for clinical application, fit-for-purpose methods to evaluate relevant CQAs, and reported specifications allowing the comparison of acceptance criteria.

DISCUSSION

In this systematic review, we provided a comprehensive overview of the quality and non-clinical testing strategies reported for various iPSC- and ESC-derived medicinal products in recent years. An inclusive literature search yielded reports on diverse PSC-derived products from different geographical regions, many of which have passed a regulatory checkpoint before starting clinical trials. We grouped a wide range of analyzed CQAs with regards to PSC-derived cell product identity, purity, content, safety, potency, and stability, constructing a traceable dataset for delving into product-specific cases of interest. Using a custom ranking system for quality tests, we ranked the methods revealing the trends in the most frequently assessed CQAs and preferred techniques for general characterization as well as for release testing. A summary of all techniques ranked as QC and QCu is intended to serve as a “QC menu card” listing recognized methods employed for assessing CQAs of different PSC-derived medicinal products during product release. Meanwhile, the product-specific overview with release test specifications is meant to provide a starting point for developers designing a QC testing panel using earlier experience as a blueprint.

This work was focused on terminally differentiated products derived from iPSCs and ESCs, which are the two main types of PSCs used to generate medicinal products (Kirkeby et al., 2025). In our analysis, we encountered a lower-than-expected testing frequency for some CQAs, such as viral safety and genomic integrity assessment. A possible explanation for missing seemingly critical analyses could be the use of interchangeable approaches, or manufacturing in-process controls and upstream QC tests performed on starting materials and intermediate products, such as donor screening and Master Cell Bank validation, falling beyond the scope of this review. It is also possible that the under-representation of certain testing modalities relates to the fact that some QC and non-clinical tests performed on the final formulation of the products may be undisclosed, reflecting publication bias, or may have been published as part of the pre-clinical development prior to the established cut-off for record retrieval. The need to include additional cross-referenced reports manually (Izrael et al., 2018; Manley et al., 2017; Surendran et al., 2021; Wu et al., 2020) demonstrated the fragmented reporting of test panels. Therefore, overall conclusions about the completeness of the testing strategies cannot be drawn from this dataset. Standardized and complete reporting of testing panels, applied methods, and acceptance criteria could improve the comparability of the studies and adaptation for future products, expediting product development, regulatory review process, and patient access to novel therapies worldwide.

The “QC menu” of recognized release tests can furthermore be extended with the techniques ranked as pQC tests, comprising state-of-the-art methods undergoing validation for this purpose or indicated as provisional QC release tests for developed products which were not yet applied in clinical trials at the time of data analysis. The field of PSC-derived therapies remains a dynamic domain in which novel assay developments and improvements are encouraged to accommodate the diversity of the final products and establish more rapid, sensitive, and cheaper procedures (O’Shea et al., 2020). The laborious *in vivo* and *in vitro* assays for adventitious virus detection, which can be limited by low detection sensitivity, variable cell line susceptibility, and long testing time, have the prospect to be replaced by the NGS (ICH, 2023). Possibly due to the relatively recent regulatory acceptance of the revised ICH Q5A(R2) guideline in 2023 (ICH, 2023), NGS application for adventitious virus testing of the final products was not observed in the included reports. Standardization of testing panels and defining minimum technical requirements such as the sequencing depth appropriate for adventitious virus or genomic integrity evaluation could contribute to a wider implementation of NGS in QC testing. During our systematic search, several novel methods for QC testing of PSC-derived cells have been retrieved, which were excluded from the main analysis due to early development stages and limited fit-for-purpose evidence. A few promising methods which addressed a clear CQA-specific challenge and were proposed for QC testing are Raman imaging of metabolites for assessing cellular purity (Li et al., 2023); multifunctional microelectrode array detecting exocytosis for cell functionality (He et al., 2022); atomic force microscopy evaluating nanomechanical properties for functional evaluation (Herardot et al., 2024); and artificial intelligence (AI) algorithms of cell morphology supporting identity assessment (Lien et al.,

2023; Marzec-Schmidt et al., 2023). Other up-and-coming techniques currently transforming the field are multi- and spatial omics coupled with powerful AI-assisted analysis algorithms (Gong et al., 2025; Yang et al., 2025), which could be especially valuable for assessing engineered tissue constructs at a cellular level while preserving the architecture which defines product's quality and functionality. Moreover, all these established and novel techniques can comprise not only the final product testing strategies, but could also support in-process control, comparability studies, and long-term evaluation throughout the product life cycle. Robust comparability assessments may be particularly important for PSC-derived medicinal products, as manufacturing processes often evolve due to process modifications, production scaling, or manufacturing site transfer, requiring demonstration that CQAs remain consistent throughout development, upscaling, and commercialization.

In conclusion, this review creates a consolidated resource of recent QC and non-clinical testing practices for PSC-derived products. Insights into state-of-the-art and established fit-for-purpose testing methods, revealed by the custom ranking system, can benefit product developers and regulatory authorities by providing a toolkit for devising future QC strategies. Taken together, this review highlights both universal methods and product-specific solutions, identifies gaps, and points to the need for harmonization, transparent reporting, and continued innovation to ensure safe and effective clinical translation of PSC-derived therapies.

ACKNOWLEDGMENTS

This work was supported by the Novo Nordisk Foundation Center for Stem Cell Medicine reNEW (NNF21CC0073729).

AUTHOR CONTRIBUTIONS

A.G., conceptualization, methodology, investigation, validation, formal analysis, data curation, visualization, writing – original draft, and writing – review and editing; M.T., conceptualization, methodology, investigation, validation, formal analysis, supervision, and writing – review and editing; I.v.A.-W., investigation, validation, and writing – review and editing; F.L., investigation and validation; P.G., methodology; B.R., supervision and writing – review and editing; E.d.K., supervision, funding acquisition, and writing – review and editing; F.C., supervision, funding acquisition, and writing – review and editing; P.M., conceptualization, supervision, project administration, funding acquisition, and writing – review and editing.

DECLARATION OF INTERESTS

The authors declare no competing interests.

SUPPLEMENTAL INFORMATION

Supplemental information can be found online at https://doi.org/10.1016/j.stemcr.2026.103084.

REFERENCES

Barry, J., Abranches, E., Baptista, R.P., Barry, C., Brandsten, C., Bruce, K., Carpenter, M.K., Cavagnaro, J., Collins, L., Csaszar, E., et al. (2026). Charting the translational pathway: ISSCR best practices for the development of PSC-derived therapies. Stem Cell Rep. *21*, 102784. https://doi.org/10.1016/j.stemcr.2025.102784.

Bershteyn, M., Bröer, S., Parekh, M., Maury, Y., Havlicek, S., Kriks, S., Fuentealba, L., Lee, S., Zhou, R., Subramanyam, G., et al. (2023). Human pallial MGE-type GABAergic interneuron cell therapy for chronic focal epilepsy. Cell Stem Cell *30*, 1331–1350.e11. https://doi.org/10.1016/j.stem.2023.08.013.

Chang, J.T., Chen, K.T., Liu, C.L., and Wang, S.R. (2001). Application of the Isoenzyme Profile in the Development of Cell-Based Devices. Acta Biotechnol. *21*, 243–253. https://doi.org/10.1002/1521-3846(200108)21:3<243::aid-abio243>3.0.co;2-o.

Chung, L., Cogburn, L.A., Sui, L., and Dashnau, J.L. (2022). Development of an induced pluripotent stem cell-specific microRNA assay for detection of residual undifferentiated cells in natural killer cell therapy products. Cytotherapy *24*, 733–741. https://doi.org/10.1016/j.jcyt.2022.02.005.

Cichocki, F., Bjordahl, R., Goodridge, J.P., Mahmood, S., Gaidarova, S., Abujarour, R., Davis, Z.B., Merino, A., Tuininga, K., Wang, H., et al. (2022). Quadruple gene-engineered natural killer cells enable multi-antigen targeting for durable antitumor activity against multiple myeloma. Nat. Commun. *13*, 7341. https://doi.org/10.1038/s41467-022-35127-2.

Corsini, M., and Mitola, S. (2023). Alternative In Vivo Models to Study Teratoma. Methods Mol. Biol. *2572*, 181–189. https://doi.org/10.1007/978-1-0716-2703-7_14.

Diaz-Hernandez, M.E., Khan, N.M., and Drissi, H. (2023). Efficient Differentiation of Human Induced Pluripotent Stem Cell (hiPSC)-Derived Mesenchymal Progenitors Into Adipocytes and Osteoblasts. Bio. Protoc. *13*, e4885. https://doi.org/10.21769/BioProtoc.4885.

Domingues, S., Darle, A., Masson, Y., Saidani, M., Lagoutte, E., Bejanariu, A., Coutier, J., Ayata, R.E., Bouschbacher, M., Peschanski, M., et al. (2022). Clinical Grade Human Pluripotent Stem Cell-Derived Engineered Skin Substitutes Promote Keratinocytes Wound Closure In Vitro. Cells *11*, 1151. https://doi.org/10.3390/cells11071151.

EDQM (European Directorate for the Quality of Medicines & HealthCare) (2025a). 2.6.1 Sterility, 11.8 (Council of Europe).

EDQM (European Directorate for the Quality of Medicines & HealthCare) (2025b). 2.6.7. Mycoplasmas, 11.8 (Council of Europe).

EDQM (European Directorate for the Quality of Medicines & HealthCare) (2025c). 2.6.14. Bacterial Endotoxins, 11.8 (Council of Europe).

EDQM (European Directorate for the Quality of Medicines & HealthCare) (2025d). 2.6.27 Microbiological Examination of Cell-Based Preparations, 11.8 (Council of Europe).

EDQM (European Directorate for the Quality of Medicines & HealthCare) (2025e). 5.1.6. Alternative Methods for Control of Microbiological Quality, 11.8 (Council of Europe).

EMA (2008). Guideline on the risk-based approach according to annex I, part IV of Directive 2001/83/EC applied to Advanced therapy medicinal products. https://www.ema.europa.eu/en/documents/scientific-guideline/guideline-risk-based-approach-according-annex-i-part-iv-directive-200183ec-applied-advanced-therapy-medicinal-products_en.pdf.

EMA (2011). Reflection paper on stem cell-based medicinal products. https://www.ema.europa.eu/en/documents/scientific-guideline/draft-reflection-paper-stem-cell-based-medicinal-products_en.pdf.

FDA (2008). 21 CFR 640.25. https://www.ecfr.gov/current/title-21/part-640/section-640.25.

FDA (2025). Good Laboratory Practice for Nonclinical Laboratory Studies. https://www.ecfr.gov/current/title-21/chapter-I/subchapter-A/part-58.

Fessler, R.G., Ehsanian, R., Liu, C.Y., Steinberg, G.K., Jones, L., Lebkowski, J.S., Wirth, E.D., and McKenna, S.L. (2022). A phase 1/2a dose-escalation study of oligodendrocyte progenitor cells in individuals with subacute cervical spinal cord injury. J. Neurosurg. Spine *37*, 812–820. https://doi.org/10.3171/2022.5.SPINE22167.

Gantier, M., Rispal, R., Fourrier, A., Ménoret, S., Delbos, F., Anegon, I., and Nguyen, T.H. (2024). Cryopreserved cGMP-compliant human pluripotent stem cell-derived hepatic progenitors rescue mice from acute liver failure through rapid paracrine effects on liver cells. Stem Cell Res. Ther. *15*, 71. https://doi.org/10.1186/s13287-024-03673-9.

Gong, Y., Fei, P., Zhang, Y., Xu, Y., and Wei, J. (2025). From Multi-Omics to Visualization and Beyond: Bridging Micro and Macro Insights in CAR-T Cell Therapy. Adv. Sci. *12*, e2501095. https://doi.org/10.1002/advs.202501095.

Gotkine, M., Caraco, Y., Lerner, Y., Blotnick, S., Wanounou, M., Slutsky, S.G., Chebath, J., Kuperstein, G., Estrin, E., Ben-Hur, T., et al. (2023). Safety and efficacy of first-in-man intrathecal injection of human astrocytes (AstroRx) in ALS patients: phase I/IIa clinical trial results. J. Transl. Med. *21*, 122. https://doi.org/10.1186/s12967-023-03903-3.

Hayakawa, T., Aoi, T., Umezawa, A., Ozawa, K., Sato, Y., Sawa, Y., Matsuyama, A., Yamanaka, S., and Yamato, M. (2015a). A study on ensuring the quality and safety of pharmaceuticals and medical devices derived from processing of allogeneic human induced pluripotent stem(-Like) cells. Regen. Ther. *2*, 95–108. https://doi.org/10.1016/j.reth.2015.06.004.

Hayakawa, T., Aoi, T., Umezawa, A., Ozawa, K., Sato, Y., Sawa, Y., Matsuyama, A., Yamanaka, S., and Yamato, M. (2015b). A study on ensuring the quality and safety of pharmaceuticals and medical devices derived from processing of autologous human induced pluripotent stem(-like) cells. Regen. Ther. *2*, 81–94. https://doi.org/10.1016/j.reth.2015.06.002.

Hayakawa, T., Aoi, T., Umezawa, A., Ozawa, K., Sato, Y., Sawa, Y., Matsuyama, A., Yamanaka, S., and Yamato, M. (2015c). A study on ensuring the quality and safety of pharmaceuticals and medical devices derived from the processing of human embryonic stem cells. Regen. Ther. *2*, 109–122. https://doi.org/10.1016/j.reth.2015.06.001.

He, E., Zhou, Y., Luo, J., Xu, S., Zhang, K., Song, Y., Wang, M., Xu, S., Dai, Y., Yang, G., et al. (2022). Sensitive detection of electrophysiology and dopamine vesicular exocytosis of hESC-derived dopaminergic neurons using multifunctional microelectrode array. Biosens. Bioelectron. *209*, 114263. https://doi.org/10.1016/j.bios.2022.114263.

Herardot, E., Liboz, M., Lamour, G., Malo, M., Plancheron, A., Habeler, W., Geiger, C., Frank, E., Campillo, C., Monville, C., and Ben M'Barek, K. (2024). Biomechanical Characterization of Retinal Pigment Epitheliums Derived from hPSCs Using Atomic Force Microscopy. Stem Cell Rev. Rep. *20*, 1340–1352. https://doi.org/10.1007/s12015-024-10717-3.

Heslop, J.A., Hammond, T.G., Santeramo, I., Tort Piella, A., Hopp, I., Zhou, J., Baty, R., Graziano, E.I., Proto Marco, B., Caron, A., et al. (2015). Concise review: workshop review: understanding and assessing the risks of stem cell-based therapies. Stem Cells Transl. Med. *4*, 389–400. https://doi.org/10.5966/sctm.2014-0110.

Hirai, T., Yasuda, S., Umezawa, A., and Sato, Y. (2023). Country-specific regulation and international standardization of cell-based therapeutic products derived from pluripotent stem cells. Stem Cell Rep. *18*, 1573–1591. https://doi.org/10.1016/j.stemcr.2023.05.003.

Hirami, Y., Mandai, M., Sugita, S., Maeda, A., Maeda, T., Yamamoto, M., Uyama, H., Yokota, S., Fujihara, M., Igeta, M., et al. (2023). Safety and stable survival of stem-cell-derived retinal organoid for 2 years in patients with retinitis pigmentosa. Cell Stem Cell *30*, 1585–1596.e1586. https://doi.org/10.1016/j.stem.2023.11.004.

Huang, L., Zhang, S., Wu, J., Guo, B., Gao, T., Shah, S.Z.A., Huang, B., Li, Y., Zhu, B., Fan, J., et al. (2023). Immunity-and-matrix-regulatory cells enhance cartilage regeneration for meniscus injuries: a phase I dose-escalation trial. Signal Transduct. Targeted Ther. *8*, 417. https://doi.org/10.1038/s41392-023-01670-7.

ICH (1997). Derivation and Characterisation of Cell Substrates Used for Production of Biotechnological/Biological Products (Q5D) (ICH).

ICH (1999). Specifications: Test Procedures And Acceptance Criteria For New Drug Substances And New Drug Products: Chemical Substances (Q6A) (ICH).

ICH (2008). Pharmaceutical Quality System (Q10) (ICH).

ICH (2009). Pharmaceutical Development Q8(R2) (ICH).

ICH (2023). Viral Safety Evaluation of Biotechnology Products Derived from Cell Lines of Human or Animal Origin (Q5A(R2)) (ICH).

Iworima, D.G., Baker, R.K., Ellis, C., Sherwood, C., Zhan, L., Rezania, A., Piret, J.M., and Kieffer, T.J. (2024). Metabolic switching, growth kinetics and cell yields in the scalable manufacture of stem cell-derived insulin-producing cells. Stem Cell Res. Ther. *15*, 1. https://doi.org/10.1186/s13287-023-03574-3.

Izrael, M., Slutsky, S.G., Admoni, T., Cohen, L., Granit, A., Hasson, A., Itskovitz-Eldor, J., Krush Paker, L., Kuperstein, G., Lavon, N., et al. (2018). Safety and efficacy of human embryonic stem

cell-derived astrocytes following intrathecal transplantation in SOD1(G93A) and NSG animal models. Stem Cell Res. Ther. *9*, 152. https://doi.org/10.1186/s13287-018-0890-5.

Jo, H.Y., Seo, H.H., Gil, D., Park, Y., Han, H.J., Han, H.W., Thimmulappa, R.K., Kim, S.C., and Kim, J.H. (2021). Single-Cell RNA Sequencing of Human Pluripotent Stem Cell-Derived Macrophages for Quality Control of The Cell Therapy Product. Front. Genet. *12*, 658862. https://doi.org/10.3389/fgene.2021.658862.

Kirkeby, A., Nelander, J., Hoban, D.B., Rogelius, N., Bjartmarz, H., Novo Nordisk Cell Therapy R&D, Storm, P., Fiorenzano, A., Adler, A.F., and Vale, S. (2023). Preclinical quality, safety, and efficacy of a human embryonic stem cell-derived product for the treatment of Parkinson's disease, STEM-PD. Cell Stem Cell *30*, 1299–1314.e9. https://doi.org/10.1016/j.stem.2023.08.014.

Kirkeby, A., Main, H., and Carpenter, M. (2025). Pluripotent stem-cell-derived therapies in clinical trial: A 2025 update. Cell Stem Cell *32*, 10–37. https://doi.org/10.1016/j.stem.2024.12.005.

Kobayashi, H., Tohyama, S., Ichimura, H., Ohashi, N., Chino, S., Soma, Y., Tani, H., Tanaka, Y., Yang, X., Shiba, N., et al. (2024). Regeneration of Nonhuman Primate Hearts With Human Induced Pluripotent Stem Cell-Derived Cardiac Spheroids. Circulation *150*, 611–621. https://doi.org/10.1161/CIRCULATIONAHA.123.064876.

Kobold, S., Bultjer, N., Stacey, G., Mueller, S.C., Kurtz, A., and Mah, N. (2023). History and current status of clinical studies using human pluripotent stem cells. Stem Cell Rep. *18*, 1592–1598. https://doi.org/10.1016/j.stemcr.2023.03.005.

Kriedemann, N., Triebert, W., Teske, J., Mertens, M., Franke, A., Ullmann, K., Manstein, F., Drakhlis, L., Haase, A., Halloin, C., et al. (2024). Standardized production of hPSC-derived cardiomyocyte aggregates in stirred spinner flasks. Nat. Protoc. *19*, 1911–1939. https://doi.org/10.1038/s41596-024-00976-2.

Lemmens, M., Perner, J., Potgeter, L., Zogg, M., Thiruchelvam, S., Müller, M., Doll, T., Werner, A., Gilbart, Y., Couttet, P., et al. (2023). Identification of marker genes to monitor residual iPSCs in iPSC-derived products. Cytotherapy *25*, 59–67. https://doi.org/10.1016/j.jcyt.2022.09.010.

Li, M., Ueyama-Toba, Y., Lindley, M., Kongklad, G., Nawa, Y., Kumamoto, Y., Ishida, S., Kanda, Y., Fujita, S., Mizuguchi, H., and Fujita, K. (2023). Label-Free Evaluation of Maturation and Hepatotoxicity of Human iPSC-Derived Hepatocytes Using Hyperspectral Raman Imaging. Anal. Chem. *95*, 9252–9262. https://doi.org/10.1021/acs.analchem.3c00976.

Lien, C.Y., Chen, T.T., Tsai, E.T., Hsiao, Y.J., Lee, N., Gao, C.E., Yang, Y.P., Chen, S.J., Yarmishyn, A.A., Hwang, D.K., et al. (2023). Recognizing the Differentiation Degree of Human Induced Pluripotent Stem Cell-Derived Retinal Pigment Epithelium Cells Using Machine Learning and Deep Learning-Based Approaches. Cells *12*, 211. https://doi.org/10.3390/cells12020211.

Luciani, M., Garsia, C., Mangiameli, E., Meneghini, V., and Gritti, A. (2022). Intracerebroventricular transplantation of human iPSC-derived neural stem cells (hiPSC-NSCs) into neonatal mice. Methods Cell Biol. *171*, 127–147. https://doi.org/10.1016/bs.mcb.2022.04.007.

Madrid, M., Lakshmipathy, U., Zhang, X., Bharti, K., Wall, D.M., Sato, Y., Muschler, G., Ting, A., Smith, N., Deguchi, S., et al. (2024). Considerations for the development of iPSC-derived cell therapies: a review of key challenges by the JSRM-ISCT iPSC Committee. Cytotherapy *26*, 1382–1399. https://doi.org/10.1016/j.jcyt.2024.05.022.

Manley, N.C., Priest, C.A., Denham, J., Wirth, E.D., and Lebkowski, J.S. (2017). Human Embryonic Stem Cell-Derived Oligodendrocyte Progenitor Cells: Preclinical Efficacy and Safety in Cervical Spinal Cord Injury. Stem Cells Transl. Med. *6*, 1917–1929. https://doi.org/10.1002/sctm.17-0065.

Marzec-Schmidt, K., Ghosheh, N., Stahlschmidt, S.R., Küppers-Munther, B., Synnergren, J., and Ulfenborg, B. (2023). Artificial Intelligence Supports Automated Characterization of Differentiated Human Pluripotent Stem Cells. Stem Cell. *41*, 850–861. https://doi.org/10.1093/stmcls/sxad049.

Masumoto, K., Aihara, Y., Miyagawa Kuroishi, M., Maeda, N., Sakai, Y., Oka, Y., Takahashi, Y., Oda, K., and Yanagida, M. (2022). Highly sensitive and non-disruptive detection of residual undifferentiated cells by measuring miRNAs in culture supernatant. Sci. Rep. *12*, 10351. https://doi.org/10.1038/s41598-022-14273-z.

McKenna, S.L., Ehsanian, R., Liu, C.Y., Steinberg, G.K., Jones, L., Lebkowski, J.S., Wirth, E., and Fessler, R.G. (2022). Ten-year safety of pluripotent stem cell transplantation in acute thoracic spinal cord injury. J. Neurosurg. Spine *37*, 321–330. https://doi.org/10.3171/2021.12.SPINE21622.

Miyagawa, S., Kainuma, S., Kawamura, T., Suzuki, K., Ito, Y., Iseoka, H., Ito, E., Takeda, M., Sasai, M., Mochizuki-Oda, N., et al. (2022). Case report: Transplantation of human induced pluripotent stem cell-derived cardiomyocyte patches for ischemic cardiomyopathy. Front. Cardiovasc. Med. *9*, 950829. https://doi.org/10.3389/fcvm.2022.950829.

Miyagawa, S., Kawamura, T., Ito, E., Takeda, M., Iseoka, H., Yokoyama, J., Harada, A., Mochizuki-Oda, N., Imanishi-Ochi, Y., Li, J., et al. (2024). Pre-clinical evaluation of the efficacy and safety of human induced pluripotent stem cell-derived cardiomyocyte patch. Stem Cell Res. Ther. *15*, 73. https://doi.org/10.1186/s13287-024-03690-8.

Montenegro, L.R., Lerário, A.M., Nishi, M.Y., Jorge, A.A.L., and Mendonca, B.B. (2021). Performance of mutation pathogenicity prediction tools on missense variants associated with 46,XY differences of sex development. Clinics *76*, e2052. https://doi.org/10.6061/clinics/2021/e2052.

Murphy, S., and Gardner, F.H. (1975). Platelet storage at 22 degrees C: role of gas transport across plastic containers in maintenance of viability. Blood *46*, 209–218. https://doi.org/10.1182/blood.V46.2.209.209.

Naderi, S., Shiri, Z., Zarei-Kheirabadi, M., Mollamohammadi, S., Hosseini, P., Rahimi, G., Moradmand, A., Samadian, A., Shojaei, A., Yeganeh, M., et al. (2023). Cryopreserved clinical-grade human embryonic stem cell-derived dopaminergic progenitors function in Parkinson's disease models. Life Sci. *329*, 121990. https://doi.org/10.1016/j.lfs.2023.121990.

Neumayer, G., Torkelson, J.L., Li, S., McCarthy, K., Zhen, H.H., Vangipuram, M., Mader, M.M., Gebeyehu, G., Jaouni, T.M., Jaców-Malinowska, J., et al. (2024). A scalable and cGMP-compatible autologous organotypic cell therapy for Dystrophic

Epidermolysis Bullosa. Nat. Commun. *15*, 5834. https://doi.org/10.1038/s41467-024-49400-z.

Oh, S., Gu, E.Y., Han, J.S., Lee, B.S., Moon, K.S., Kim, Y.B., and Han, K.H. (2022). Tumorigenicity Assessment of Human Cancer Cell Lines Xenografted on Immunodeficient Mice as Positive Controls of Tumorigenicity Testing. Int. J. Toxicol. *41*, 476–487. https://doi.org/10.1177/10915818221124573.

O'Shea, O., Steeg, R., Chapman, C., Mackintosh, P., and Stacey, G.N. (2020). Development and implementation of large-scale quality control for the European bank for induced Pluripotent Stem Cells. Stem Cell Res. *45*, 101773. https://doi.org/10.1016/j.scr.2020.101773.

Park, J.W., Bae, S.J., Yun, J.H., Kim, S., and Park, M. (2024a). Assessment of Genetic Stability in Human Induced Pluripotent Stem Cell-Derived Cardiomyocytes by Using Droplet Digital PCR. Int. J. Mol. Sci. *25*, 1101. https://doi.org/10.3390/ijms25021101.

Park, S., Park, C.W., Eom, J.H., Jo, M.Y., Hur, H.J., Choi, S.K., Lee, J.S., Nam, S.T., Jo, K.S., Oh, Y.W., et al. (2024b). Preclinical and dose-ranging assessment of hESC-derived dopaminergic progenitors for a clinical trial on Parkinson's disease. Cell Stem Cell *31*, 25–38.e8. https://doi.org/10.1016/j.stem.2023.11.009.

Rong, L., Wei, W., Fang, Y., Liu, Y., Gao, T., Wang, L., Hao, J., Gu, X., Wu, J., and Wu, W. (2024). Clinical-grade human embryonic stem cell-derived mesenchymal stromal cells ameliorate diabetic retinopathy in db/db mice. Cytotherapy *26*, 606–615. https://doi.org/10.1016/j.jcyt.2024.02.020.

Sato, Y., Bando, H., Di Piazza, M., Gowing, G., Herberts, C., Jackman, S., Leoni, G., Libertini, S., MacLachlan, T., McBlane, J.W., et al. (2019). Tumorigenicity assessment of cell therapy products: The need for global consensus and points to consider. Cytotherapy *21*, 1095–1111. https://doi.org/10.1016/j.jcyt.2019.10.001.

Sekine, O., Kanaami, S., Masumoto, K., Aihara, Y., Morita-Umei, Y., Tani, H., Soma, Y., Umei, T.C., Haga, K., Moriwaki, T., et al. (2023). Seamless and non-destructive monitoring of extracellular microRNAs during cardiac differentiation from human pluripotent stem cells. Stem Cell Rep. *18*, 1925–1939. https://doi.org/10.1016/j.stemcr.2023.08.011.

Shi, H., Feng, T., Wang, R., Wu, L., and Gu, Y. (2022). Universal Markers for hiPSCs Residue Detection. Front. Biosci. *27*, 239. https://doi.org/10.31083/j.fbl2708239.

Shigyo, M., Kobayashi, Y., Platoshyn, O., Marsala, S., Kato, T., Jr., Takamura, N., Yoshida, K., Kishino, A., Bravo-Hernandez, M., Juhas, S., et al. (2023). Derivation of Sendai-Virus-Reprogrammed Human iPSCs-Neuronal Precursors: In Vitro and In Vivo Post-grafting Safety Characterization. Cell Transplant. *32*, 9636897231163232. https://doi.org/10.1177/09636897231163232.

Shin, J.H., Ryu, C.M., Yu, H.Y., Park, J., Kang, A.R., Shin, J.M., Hong, K.S., Kim, E.Y., Chung, H.M., Shin, D.M., and Choo, M.S. (2022). Safety of Human Embryonic Stem Cell-derived Mesenchymal Stem Cells for Treating Interstitial Cystitis: A Phase I Study. Stem Cells Transl. Med. *11*, 1010–1020. https://doi.org/10.1093/stcltm/szac065.

Soma, T., Oie, Y., Takayanagi, H., Matsubara, S., Yamada, T., Nomura, M., Yoshinaga, Y., Maruyama, K., Watanabe, A., Takashima, K., et al. (2024). Induced pluripotent stem-cell-derived corneal epithelium for transplant surgery: a single-arm, open-label, first-in-human interventional study in Japan. Lancet *404*, 1929–1939. https://doi.org/10.1016/s0140-6736(24)01764-1.

Sugimoto, N., Kanda, J., Nakamura, S., Kitano, T., Hishizawa, M., Kondo, T., Shimizu, S., Shigemasa, A., Hirai, H., Arai, Y., et al. (2022a). iPLAT1: the first-in-human clinical trial of iPSC-derived platelets as a phase 1 autologous transfusion study. Blood *140*, 2398–2402. https://doi.org/10.1182/blood.2022017296.

Sugimoto, N., Nakamura, S., Shimizu, S., Shigemasa, A., Kanda, J., Matsuyama, N., Tanaka, M., Hayashi, T., Fuchizaki, A., Nogawa, M., et al. (2022b). Production and nonclinical evaluation of an autologous iPSC-derived platelet product for the iPLAT1 clinical trial. Blood Adv. *6*, 6056–6069. https://doi.org/10.1182/bloodadvances.2022008512.

Sullivan, S., Stacey, G.N., Akazawa, C., Aoyama, N., Baptista, R., Bedford, P., Bennaceur Griscelli, A., Chandra, A., Elwood, N., Girard, M., et al. (2018). Quality control guidelines for clinical-grade human induced pluripotent stem cell lines. Regen. Med. *13*, 859–866. https://doi.org/10.2217/rme-2018-0095.

Sun, J., Yates, C., Dingwall, S., Ongtengco, C., Power, D., Gray, P., and Prowse, A. (2024). Analysis of three characterization assays reveals ddPCR of LIN28A as the most sensitive for the detection of residual pluripotent stem cells in cellular therapy products. Cytotherapy *26*, 1374–1381. https://doi.org/10.1016/j.jcyt.2024.05.019.

Surendran, H., Nandakumar, S., Reddy K, V.B., Stoddard, J., Mohan K, V., Upadhyay, P.K., McGill, T.J., and Pal, R. (2021). Transplantation of retinal pigment epithelium and photoreceptors generated concomitantly via small molecule-mediated differentiation rescues visual function in rodent models of retinal degeneration. Stem Cell Res. Ther. *12*, 70. https://doi.org/10.1186/s13287-021-02134-x.

Surendran, H., Soundararajan, L., Reddy K, V.B., Subramani, J., Stoddard, J., Reynaga, R., Tschetter, W., Ryals, R.C., and Pal, R. (2022). An improved protocol for generation and characterization of human-induced pluripotent stem cell-derived retinal pigment epithelium cells. STAR Protoc. *3*, 101803. https://doi.org/10.1016/j.xpro.2022.101803.

The European Parliament And The Council Of The European Union (2004). Directive on the harmonisation of laws, regulations and administrative provisions relating to the application of the principles of good laboratory practice and the verification of their applications for tests on chemical substances (codified version). https://eur-lex.europa.eu/LexUriServ/LexUriServ.do?uri=OJ:L:2004:050:0044:0059:en:PDF.

Tsujimoto, H., Katagiri, N., Ijiri, Y., Sasaki, B., Kobayashi, Y., Mima, A., Ryosaka, M., Furuyama, K., Kawaguchi, Y., and Osafune, K. (2022). In vitro methods to ensure absence of residual undifferentiated human induced pluripotent stem cells intermingled in induced nephron progenitor cells. PLoS One *17*, e0275600. https://doi.org/10.1371/journal.pone.0275600.

Wang, S., Du, Y., Zhang, B., Meng, G., Liu, Z., Liew, S.Y., Liang, R., Zhang, Z., Cai, X., Wu, S., et al. (2024). Transplantation of chemically induced pluripotent stem-cell-derived islets under abdominal anterior rectus sheath in a type 1 diabetes patient. Cell *187*, 6152–6164.e18. https://doi.org/10.1016/j.cell.2024.09.004.

Watanabe, T., Yasuda, S., Chen, C.L., Delsing, L., Fellows, M.D., Foldes, G., Kusakawa, S., Mouriès, L.P., and Sato, Y. (2023). International evaluation study of a highly efficient culture assay for detection of residual human pluripotent stem cells in cell therapies. Regen. Med. *18*, 219–227. https://doi.org/10.2217/rme-2022-0207.

Watanabe, T., Yasuda, S., Kusakawa, S., Kuroda, T., Furukawa, H., Futamura, M., Shimizu, S., Morishita, A., Hata, S., Koeda, A., et al. (2024). Multisite studies for optimization of a highly efficient culture assay used for in vitro detection of residual undifferentiated human pluripotent stem cells intermingled in cell therapy products. Regen. Ther. *26*, 315–323. https://doi.org/10.1016/j.reth.2024.06.007.

Watari, K., Yamasaki, S., Tu, H.Y., Shikamura, M., Kamei, T., Adachi, H., Tochitani, T., Kita, Y., Nakamura, A., Ueyama, K., et al. (2023). Self-organization, quality control, and preclinical studies of human iPSC-derived retinal sheets for tissue-transplantation therapy. Commun. Biol. *6*, 164. https://doi.org/10.1038/s42003-023-04543-5.

Wu, J., Song, D., Li, Z., Guo, B., Xiao, Y., Liu, W., Liang, L., Feng, C., Gao, T., Chen, Y., et al. (2020). Immunity-and-matrix-regulatory cells derived from human embryonic stem cells safely and effectively treat mouse lung injury and fibrosis. Cell Res. *30*, 794–809. https://doi.org/10.1038/s41422-020-0354-1.

Wu, Y., Zhang, Z., Wu, S., Chen, Z., and Pu, Y. (2023). Estimating residual undifferentiated cells in human chemically induced pluripotent stem cell derived islets using lncRNA as biomarkers. Sci. Rep. *13*, 16435. https://doi.org/10.1038/s41598-023-43798-0.

Wu, J., Li, T., Guo, M., Ji, J., Meng, X., Fu, T., Nie, T., Wei, T., Zhou, Y., Dong, W., et al. (2024). Treating a type 2 diabetic patient with impaired pancreatic islet function by personalized endoderm stem cell-derived islet tissue. Cell Discov. *10*, 45. https://doi.org/10.1038/s41421-024-00662-3.

Yang, Y., McCullough, C.G., Seninge, L., Guo, L., Kwon, W.J., Zhang, Y., Li, N.Y., Gaddam, S., Pan, C., Zhen, H., et al. (2025). A spatiotemporal and machine-learning platform facilitates the manufacturing of hPSC-derived esophageal mucosa. Dev. Cell *60*, 1359–1376.e10. https://doi.org/10.1016/j.devcel.2024.12.030.

Yasuda, S., Bando, K., Henry, M.P., Libertini, S., Watanabe, T., Bando, H., Chen, C., Fujimori, K., Harada, K., Kuroda, T., et al. (2024). Detection of residual pluripotent stem cells in cell therapy products utilizing droplet digital PCR: an international multisite evaluation study. Stem Cells Transl. Med. *13*, 1001–1014. https://doi.org/10.1093/stcltm/szae058.

Yu, J., Yu, N., Tian, Y., Fang, Y., An, B., Feng, G., Wu, J., Wang, L., Hao, J., Wang, L., et al. (2023). Safety and efficacy of human ESC-derived corneal endothelial cells for corneal endothelial dysfunction. Cell Biosci. *13*, 201. https://doi.org/10.1186/s13578-023-01145-w.

Zhao, Q., and Liu, F. (2022). Derivation and Characterization of Mesenchymal Stem Cells from iPS Cells. Methods Mol. Biol. *2454*, 443–454. https://doi.org/10.1007/7651_2020_327.

Stem Cell Reports, Volume *21*

Supplemental Information

Quality control and non-clinical testing of iPSC- and ESC-derived medicinal products: A systematic review

Akvilė Gasiūnaitė, Mara Tihaya, Inge van Amerongen-Westra, Fong Lin, Pema Gurung, Bahareh Rajaei, Eelco de Koning, Françoise Carlotti, and Pauline Meij

Supplemental figures

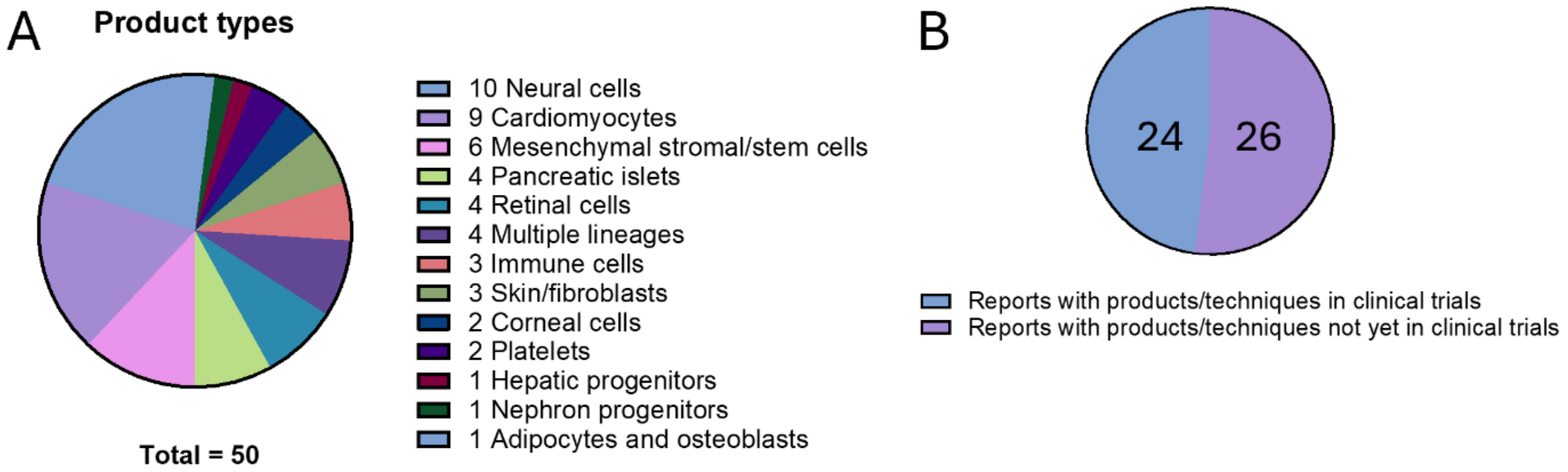

Figure S1. An overview of the included reports. A) Number of reports on PSC-derived products grouped by the end-differentiated cell types. See also Table S1. B) Clinical implementation of cell-based products and techniques in the included reports.

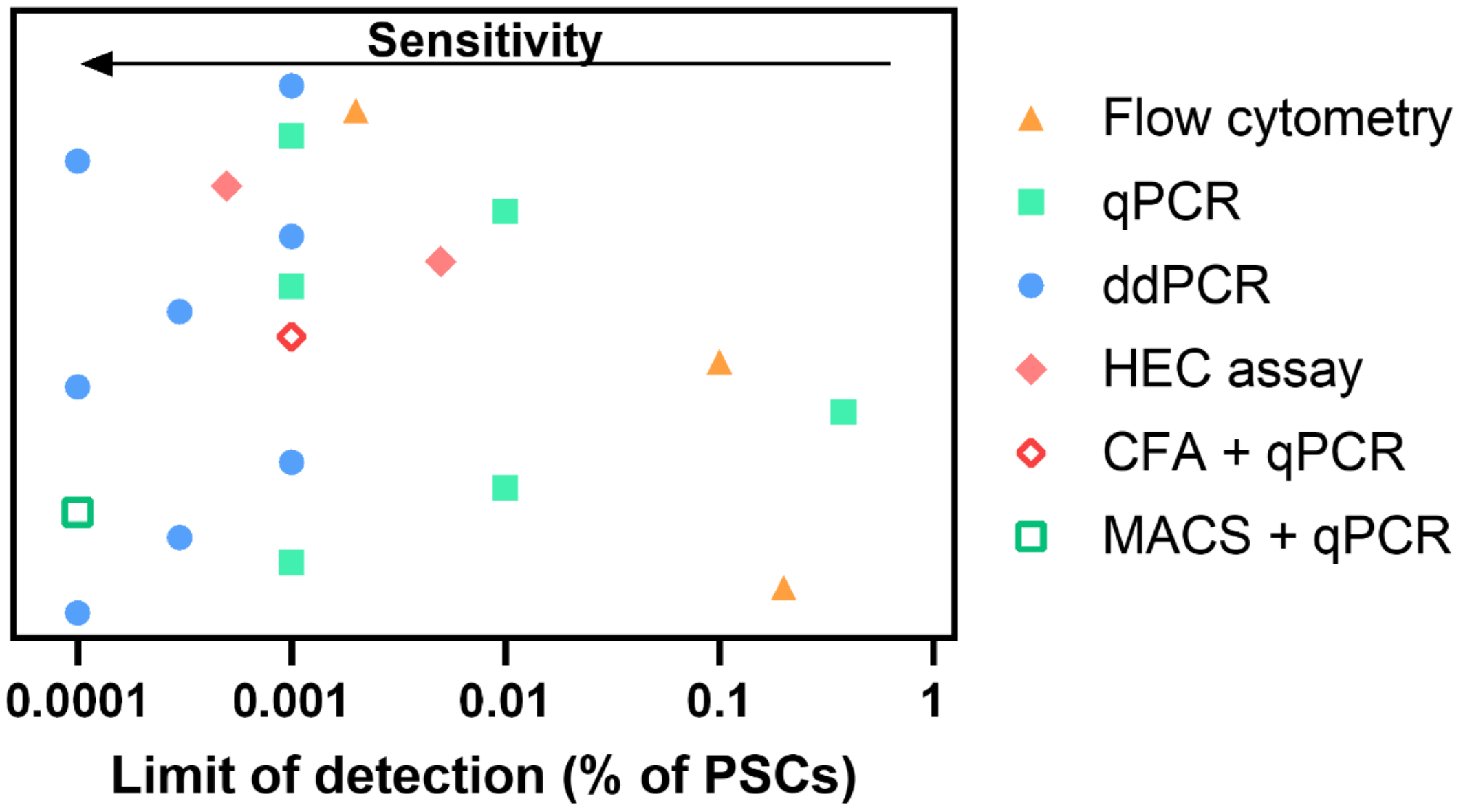

Figure S2. Limits of detection (LOD) reported for different methods employed in residual PSC detection, related to Figure 1B. The sensitivity to detect residual PSCs increases with decreasing LOD of the assay. Flow cytometry: N = 3 reports; qPCR: N = 6; ddPCR: N = 8; HEC assay: N = 2; CFA + qPCR: N = 1, MACS + qPCR: N = 1. CFA: colony formation assay, ddPCR: droplet digital PCR, HEC: highly efficient culture, MACS: magnetic-activated cell sorting, qPCR: quantitative PCR.

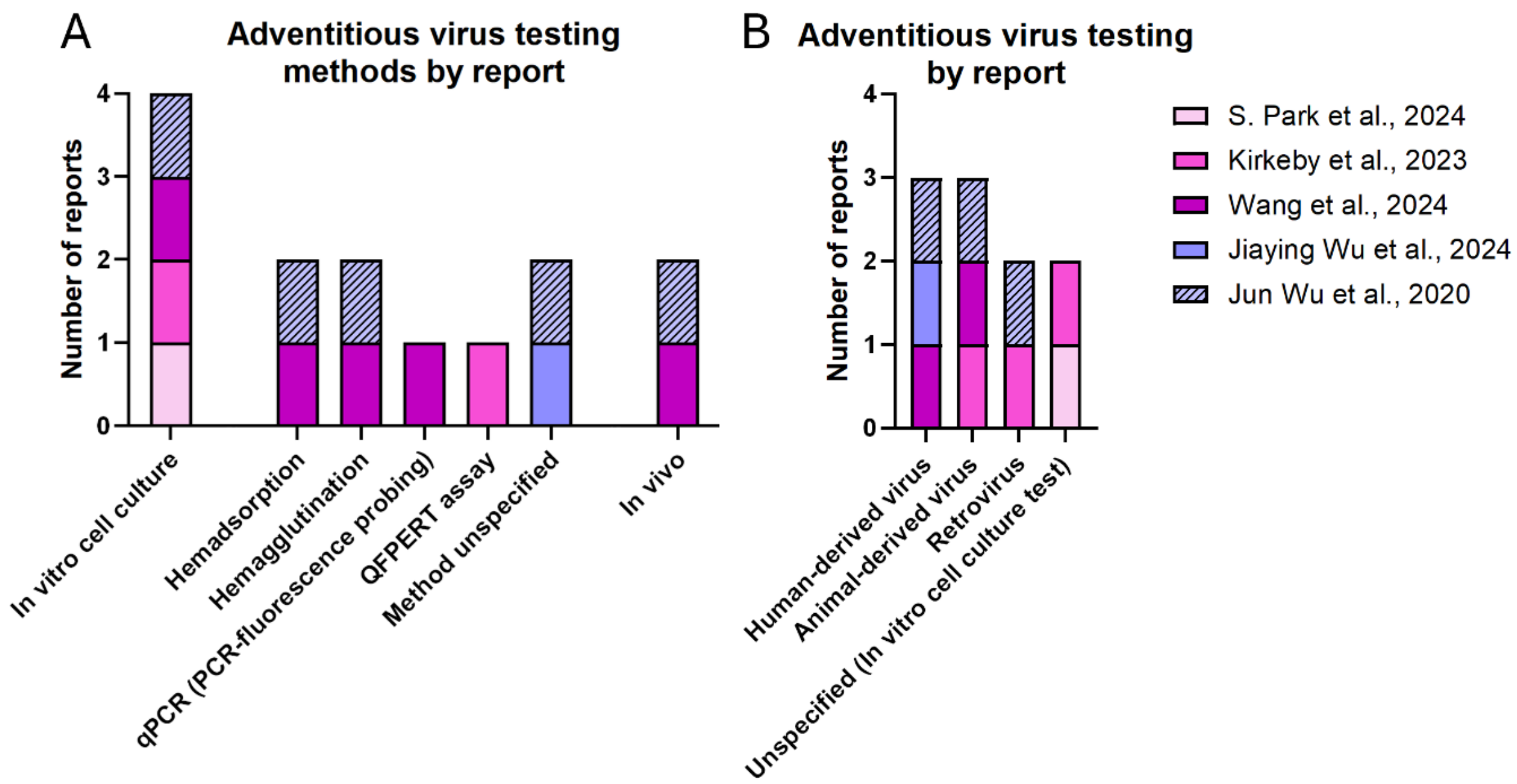

Figure S3. Reports that tested adventitious virus presence in the PSC-derived end-products, related to Figure 2. A) Graphical representation of different methods used for adventitious virus detection in each report. See also Figure 2C. B) Virus types and species targeted for detection. See also Figure 2D. qPCR: quantitative PCR, QFPERT: quantitative fluorescent product-enhanced reverse transcriptase.

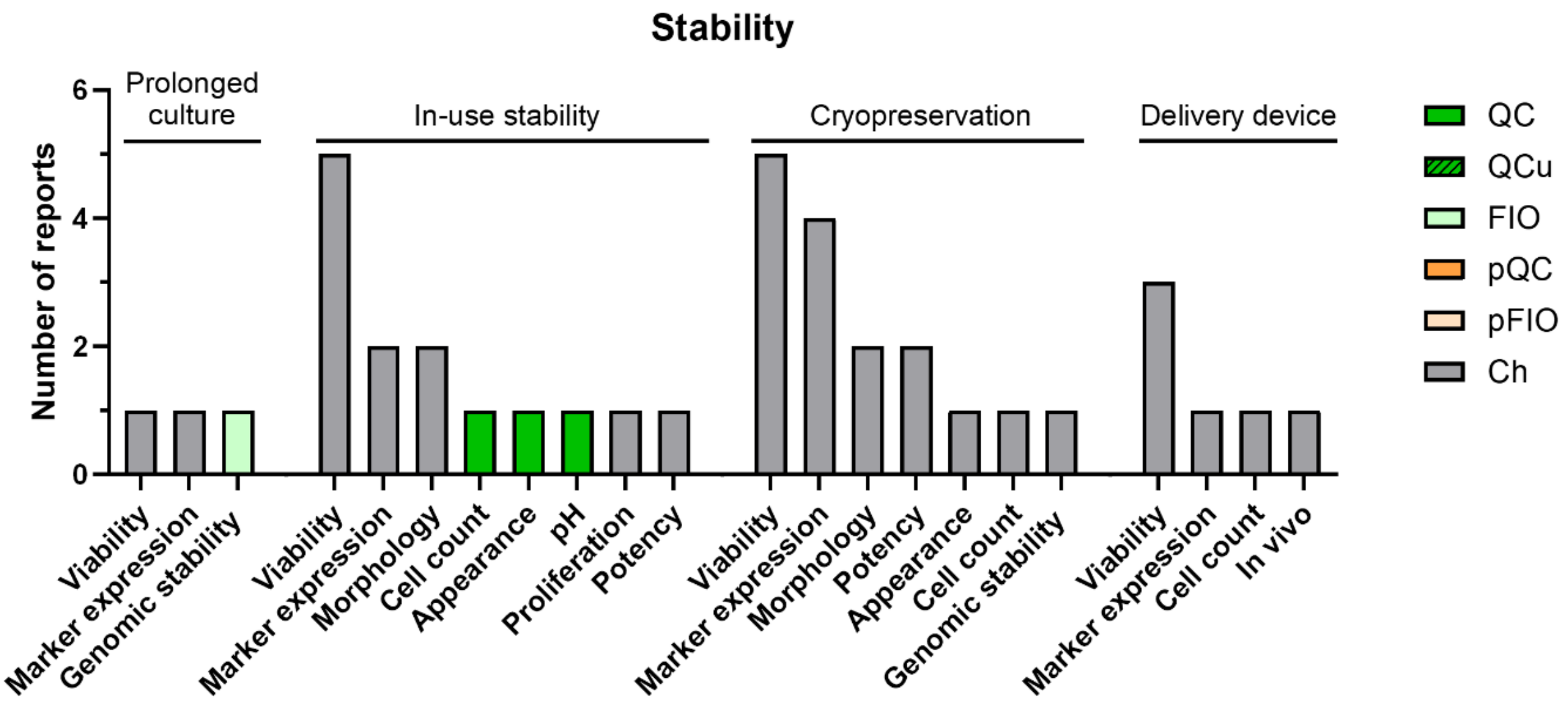

Figure S4. Stability testing of PSC-derived therapy products, related to Table S9. Different CQAs that were assessed to evaluate the effects of post-manufacturing product handling procedures, such as prolonged culture, in-use stability (during room temperature preservation, storage in the formulation solution, and transportation), cryopreservation, and delivery through an injection device.

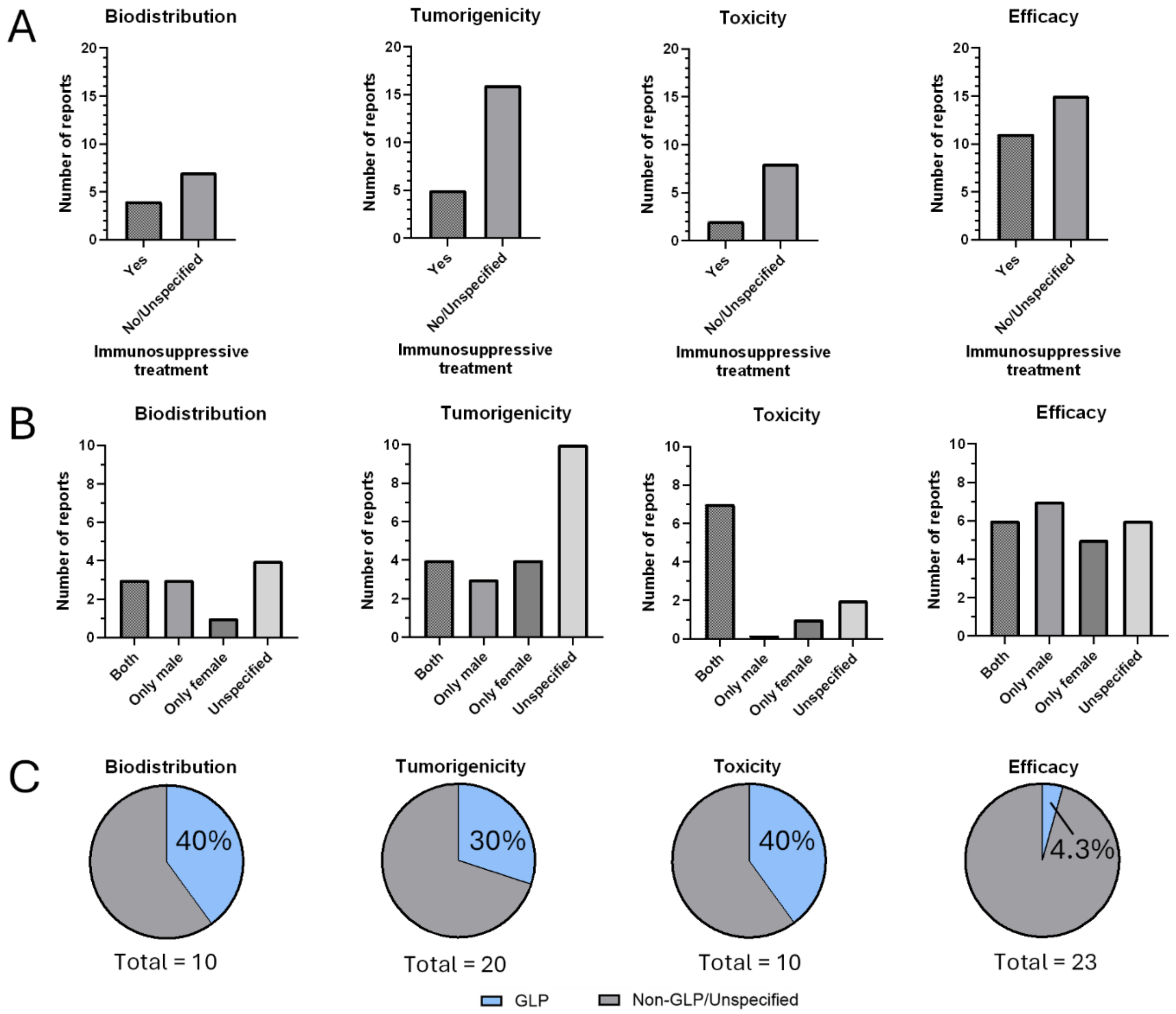

Figure S5. Animal model characteristics and study conditions in non-clinical assessment of PSC-derived therapy products, related to Figure 4 and Tables S10-S13. A) Use of the immunosuppressive drugs in animal studies. B) Use of male and female animals for PSC-derived product testing. C) Percentage of reports which applied GLP conditions in the non-clinical studies.

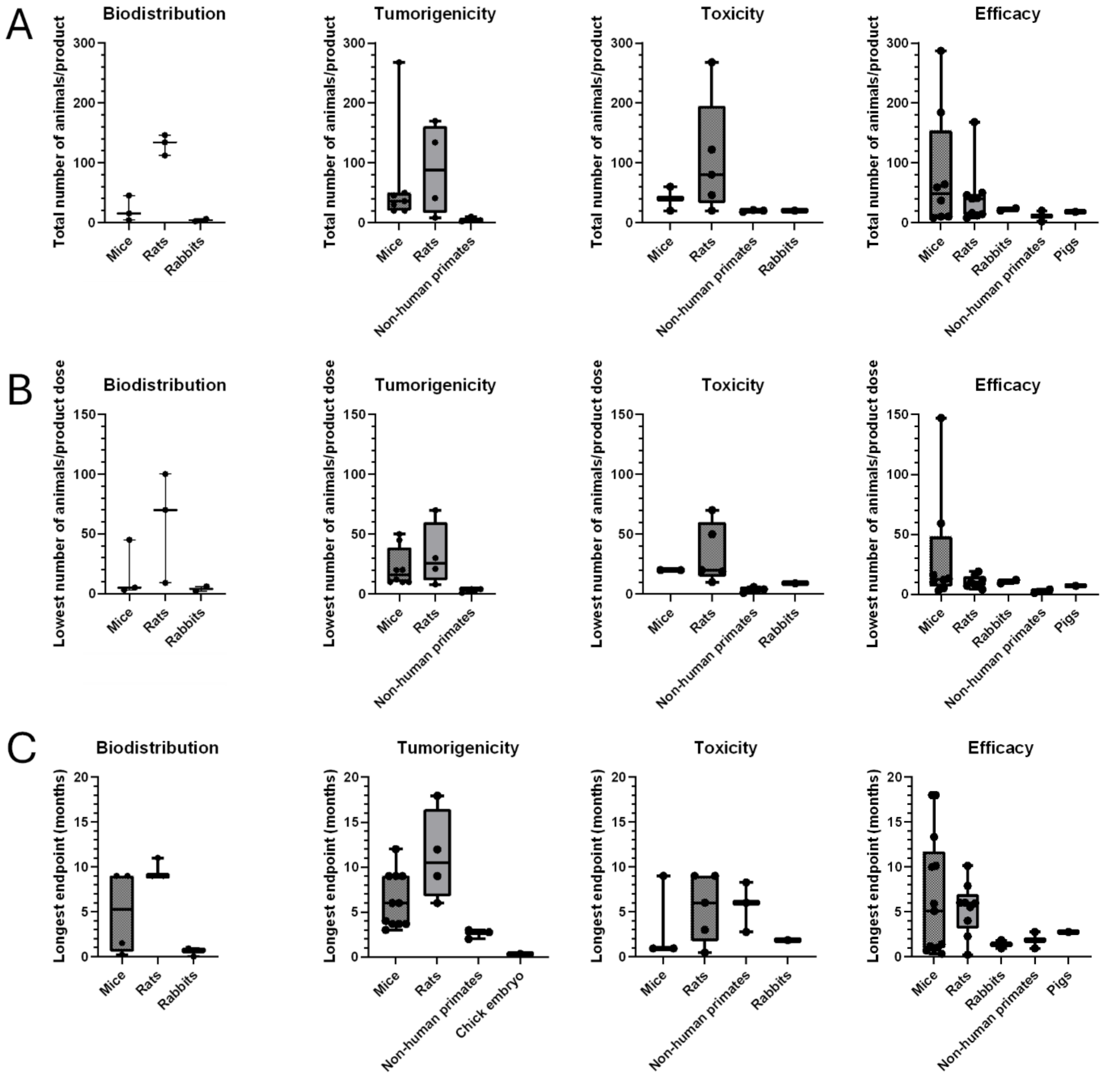

Figure S6. Animal numbers and follow-up duration in non-clinical assessment of PSC-derived therapy products, related to Figure 4 and Tables S10-S13. A) Total number of animals, including all dose groups, controls, and spares, used to test a single PSC-derived product described in the included reports. B) Lowest indicated number of animals used to test a single dose of the product. C) Longest endpoint of the follow-up duration in non-clinical studies per animal species. Data are represented as box plots with median line and whiskers showing minimum and maximum values. Individual dots represent the summed animal number per specific product.

Supplemental tables

Table S1. PSC-derived product types and their intended indications in the included reports, related to Figure S1.

Product type	Product	Disease	Reference numbers
Neural cells	Neural stem cells	Early-onset neurodegenerative or demyelinating CNS diseases	[1]
	Neural precursor cells	Spinal neurodegenerative disorders (spinal ischemic/traumatic injury, ALS, or multiple sclerosis)	[2]
	Dopaminergic progenitors	Parkinson’s disease	[3-5]
	Oligodendrocyte progenitor cells	Cervical spinal cord injury	[6, 7]
	Interneurons	Epilepsy	[8]
	Astrocytes	Amyotrophic lateral sclerosis	[9, 10]
Cardiomyocytes	Single cells	Heart failure	[11-15]
	Aggregates		[13, 16, 17]
	Patches		[18, 19]
MSCs	MSCs	Diabetic retinopathy, Interstitial cystitis	[20-23]
	IMRCs	Meniscus injury, Lung injury and fibrosis	[24, 25]
Retinal cells	Photoreceptors (retinal sheets)	Retinitis pigmentosa	[26, 27]
	RPE cells (suspension)	Age-related macular degeneration	[28, 29]
Immune cells	Macrophages	Inflammatory disorders and cancer	[30]
	NK cells	Multiple myeloma	[31, 32]

Skin grafts	Fibroblasts	-	[33]
	Keratinocytes and fibroblasts	Chronic wounds	[34]
	Keratinocytes, fibroblasts, and melanocytes	Dystrophic Epidermolysis Bullosa	[35]
Corneal cells	Corneal endothelial cells	Corneal endothelial dysfunction	[36]
	Corneal epithelial cell sheets	Limbal stem-cell deficiency	[37]
Multiple lineages	Including MSCs, RPEs, neural progenitor, hepatic, and endothelial cells	-	[38-41]
Other	Islets	Type 1 and 2 Diabetes	[42-45]
	Platelets	Aplastic anemia	[46, 47]
	Hepatic progenitors	Acute liver failure	[48]
	Nephron progenitor cells	Acute kidney injury	[49]
	Adipocytes and osteoblasts	-	[50]

ALS: amyotrophic lateral sclerosis, CNS: central nervous system, IMRCs: immunity-and-matrix-regulatory cells, MSCs: mesenchymal stromal/stem cells, NK cells: natural killer cells, RPE: retinal pigment epithelium.

Table S2. Identity tests of PSC-derived therapy products and their ranking, related to Figure 1A.

CQA	Method	Ranks and Reference numbers					
		QC	QCu	FIO	pQC	pFIO	Ch
Marker expression	Flow cytometry	[3, 5, 9, 18, 37, 44, 45, 47]	[6, 22, 24, 25]		[16, 21, 34, 36]	[28]	[2, 8, 17, 20, 30, 32, 35, 42, 48]
	IF imaging	[37]	[6]	[5]	[4, 34, 36]	[28]	[1-3, 8, 10, 17, 18, 26, 42, 44, 48]
	qPCR		[6, 27]	[5]	[4, 21]	[13]	[1, 3, 18, 28, 42, 45, 48]
	RNA-sequencing (single cell, bulk)				[30]		[4, 8, 18, 25, 29, 35, 36, 44, 45]
	HLA/HPA/ABO expression		[22, 24, 25]		[21]		[20, 46, 48]
	PCR	[3]					[26]
	MR-MAIPA assay						[46]
Morphology	Microscopic evaluation (bright-field/phase contrast)	[44, 45]	[22, 25]		[4, 34, 36]	[16, 21, 50]	[3, 17, 20, 26, 28, 30, 37, 42]
	H&E/IHC staining				[34]		[18, 26, 37]
	Transmission electron microscopy						[18, 47]
	Particle size/hematology analyzer						[47]
Metabolic	Glucose utilization and lactate accumulation				[42]		
	C-peptide accumulation in the medium				[42]		

Potency-related	Differentiation potential		[22, 24]				[20, 25]
	Dithizone (DTZ) staining	[44]					[42]
	Patch clamp						[16, 17]
	Annexin V binding	[47]					
	Mineralization					[50]	
	Lipid accumulation					[50]	
	Neurotransmitter secretion						[8]
	Migration potential						[8]
Genotype	STR analysis	[5, 44]	[25]				

HLA: human leukocyte antigen, HPA: human platelet antigen, H&E: hematoxylin and eosin, IF: immunofluorescence, IHC: immunohistochemistry, MR-MAIPA: modified rapid monoclonal antibody-specific immobilization of platelet antigens, STR: short tandem repeat.

Table S3. Impurity tests of PSC-derived therapy products and their ranking, related to Figure 1B.

CQA	Method	Ranks and Reference numbers					
		QC	QCu	FIO	pQC	pFIO	Ch
Off-target cells	Flow cytometry	[3, 5, 44, 45, 47]	[6, 22, 24, 25]		[21]	[16]	[8, 18, 20, 29, 32]
	qPCR	[26]	[6]	[5]	[4, 21]	[28]	[18, 42]
	RNA-sequencing (single cell, bulk)				[30]		[4, 8, 18, 25, 29, 35, 45]
	IF imaging		[6]		[4]		[3, 8, 17, 29, 42]
	PCR						[26]
	Morphology evaluation (bright-field microscopy)						[26]
	Morphology evaluation (automated hematology analyzer)						[46]
Residual PSCs	qPCR	[3]	[27]		[21, 36, 41]	[13, 14, 28]	[1, 18, 33, 35, 48]
	Flow cytometry	[5]	[9, 22, 25]		[8, 16]	[28]	[2, 48]
	IF imaging		[25]	[5]			[1, 3, 4, 18, 29, 36, 48]
	ddPCR	[37, 44]			[15, 31, 33, 39, 43, 49]		
	In vivo (mice, rats)		[25]				[3, 36]
	RNA-sequencing (single cell, bulk)						[8, 25, 29]
	Highly Efficient Culture (HEC) Assay				[12, 23]		
	PCR		[25]				
	PCR array						[18]
	RT-LAMP						[33]
	MACS + qRT-PCR				[49]		

	Colony formation assay (CFA) + qPCR (supernatant miRNA)					[13]	
	Method unspecified						[19]
Proliferating cells	Ki67 IF imaging				[36]		[3, 8, 10, 48]
	Ki67 Flow cytometry						[29]
	EdU IF imaging						[8]
	Population doubling time/level						[3]
Transformed cells	Soft agar colony formation (SACF) assay	[44]					[18]
	Cell growth assay/ Serial passage culture test						[18, 37]
Residual raw materials	Bovine serum albumin	[44]	[25, 47]				
	Growth factors	[44]					
	Small molecules		[47]				
	Cytokines		[47]				
Interspecies contamination	STR analysis	[44]					
	Isozyme analysis		[25]				

ddPCR: droplet digital PCR, EdU: 5-ethynyl-2´-deoxyuridine, IF: immunofluorescence, MACS: magnetic-activated cell sorting, RT-LAMP: reverse transcription loop-mediated isothermal amplification, STR: short tandem repeat.

Table S4. Methods for detecting residual PSCs in PSC-derived therapy products and their limit of detection, related to Figure 1B.

Method	Ref. no.	Rank	Material	Markers	Lowest reported LOD
Flow cytometry	[16]	pQC	Cells	OCT4	- (reported result 0.163%)
	[48]	Ch	Cells	OCT4	0.2%
	[2]	Ch	Cells	NANOG	-
	[5]	QC	Cells	OCT4/NANOG	≤0.1% (release specification)
	[9]	QCu	Cells	SSEA-4, EPCAM	-
	[28]	pFIO	Cells	OCT4	- (reported result 0.51%)
	[22]	QCu	Cells	OCT4, TRA-1-60, TRA-1-81	- (reported results 0.26%, 0.05%, 0.00%)
	[25]	QCu	Cells	TRA-1-60, SSEA-4, TRA-1-81	- (reported result <0.1%)
	[8]	pQC	Cells	TRA-1-60/OCT4	0.002%
qPCR	[3]	QC	Cells	*NANOG, TDGF1, REX1*	-
	[18]	Ch	Cells	*POU5F1, SOX2, NANOG, LIN28A*	-
	[48]	Ch	Cells	*OCT4, NANOG*	-
	[36]	pQC	Cells	*OCT4*	0.001%
	[13]	pFIO	Supernatant	miR-302b-3p	0.01%
	[35]	Ch	Cells	*LIN28A, NANOG, OCT4, SOX2*	0.38% (based on *LIN28A*)
	[14]	pFIO	Cells	*LIN28A, IDO1, CNMD*	0.001%
	[27]	QCu	Cells	*OCT4*	-
	[21]	pQC	Cells	*NANOG, OCT4*	-
	[28]	pFIO	Cells	*OCT4*	-
	[41]	pQC	Supernatant	miR-302b	0.001%
	[1]	Ch	Cells	*OCT4, NANOG*	-
	[33]	Ch	Cells	*LIN28A*	0.01%

ddPCR	[43]	pQC	Cells	*LNCPRESS2, LINC00678*	0.0001%
	[49]	pQC	Cells	*MIR302CHG*	0.001%
	[39]	pQC	Cells	*ESRG, ZSCAN10*	0.0001%
	[31]	pQC	Cells	miR-302a-5p, miR-302c-3p, miR-302d-5p	0.0003% (miR-302c-3p)
	[15]	pQC	Cells	*ESRG, LINC00678, LIN28A*	0.0003%
	[33]	pQC	Cells	*LIN28A*	0.001%
	[37]	QC	Cells	*LIN28A*	0.001%
	[44]	QC	Cells	*LNCPRESS2, LINC00678*	0.0001%
HEC assay	[12]	pQC	Cells	Alkaline phosphatase (ALP)	0.0005%
	[23]	pQC	Cells	Alkaline phosphatase (ALP)	0.005%
CFA + qPCR	[13]	pFIO	Supernatant	miR-302b-3p	0.001%
MACS + qPCR	[49]	pQC	Cells	*MIR302CHG*	0.0001%

CFA: colony formation assay, ddPCR: droplet digital PCR, HEC: highly efficient culture, LOD: limit of detection, MACS: magnetic-activated cell sorting.

Markers separated by a comma: evaluated individually; markers separated by a slash: double positive.

Table S5. Content tests of PSC-derived therapy products and their ranking, related to Figure 1C.

CQA	Method	Ranks and Reference numbers					
		QC	QCu	FIO	pQC	pFIO	Ch
Viability	Automated cell counter	[3, 5, 44]	[9]		[16]		[42, 48]
	Fluorescence microscopy	[44]				[16]	
	Flow cytometry	[45]					[8]
	Method unspecified	[18, 37]			[36]		[17]
Cell count	Automated cell counter	[3, 5]	[9]				[42, 44, 48]
	Flow cytometry	[47]					
	Confocal fluorescence microscopy					[16]	
	Method unspecified	[37]					[17]
General appearance	Visual inspection	[5, 37, 47]					[19]
pH	Electrode method	[47]					

Table S6. Safety tests of PSC-derived therapy products and their ranking, related to Figure 2.

CQA	Method	Ranks and Reference numbers					
		QC	QCu	FIO	pQC	pFIO	Ch
Genomic integrity	Whole genome sequencing	[44]		[37]	[35]		[18]
	Whole exome sequencing	[44]		[37]			[11, 18]
	Karyotyping		[22]		[34]		[2, 37]
	SNP array			[5, 37]			[8, 18]
	Targeted sequencing			[5]	[35]		[8, 11]
	ddPCR				[11]		[35]
	CGH array						[19]
	Microarray						[11]
	qPCR						[11]
Tumorigenicity	Soft agar colony formation (SACF) assay						[25, 47, 48]
	Marker expression					[18]	
	Proliferation test						[46]
Adventitious virus	In vitro cell culture	[3, 5, 44]	[25]				
	Hemadsorption	[44]	[25]				
	Hemagglutination	[44]	[25]				
	qPCR (PCR-fluorescence probing)	[44]					
	QFPERT assay	[5]					
	Method unspecified	[45]	[25]				
	In vivo	[44]	[25]				

CGH: comparative genomic hybridization, ddPCR: droplet digital PCR, QFPERT: quantitative fluorescent product-enhanced reverse transcriptase, SNP: single nucleotide polymorphism.

Table S7. Comparison of NGS methods used for genomic integrity testing, related to Figure 2A.

Method	Ref. no.	Rank	Depth coverage	Panel	Number of genes
WGS	[18]	Ch	56-83x	COSMIC, Cancer Gene Census, Shibata's list	
	[35]	pQC	~ 40x	-	
	[37]	FIO	71-76x	COSMIC Cancer Gene Census, Shibata's list	
	[44]	QC	~ 40x	COSMIC, Cancer Gene Census, CliniVar	
WES	[11]	Ch	~ 100x	COSMIC	
	[18]	Ch	84-114x	COSMIC, Cancer Gene Census, Shibata's list	
	[37]	FIO	83-148x	COSMIC Cancer Gene Census, Shibata's gene list	
	[44]	QC	~ 300x	COSMIC, Cancer Gene Census, CliniVar	
Targeted sequencing	[11]	Ch	~ 300x	ONCOAccuPanel	344 solid tumor-related genes
	[35]	pQC	-	STAMP oncopanel	130 genes, LOD allele frequency 5%
	[5]	FIO	-	Illumina NGS AmpliSeq Cancer HotSpot Panel	50 genes with 2800 COSMIC mutations; sensitivity of 5% mosaicism
	[8]	Ch	At least 100x	CleanPlex TP53 Panel (Paragon Genomics)	1 gene

COSMIC: Catalogue of Somatic Mutations in Cancer, STAMP: Stanford Actionable Mutation Panel for Solid Tumors, WES: whole exome sequencing, WGS: whole genome sequencing.

Table S8. Potency tests of PSC-derived therapy products and their ranking, related to Figure 3.

CQA	Ranks and Reference numbers					
	QC	QCu	FIO	pQC	pFIO	Ch
Hormone/cytokine production/secretion	[9, 44, 45]			[28, 42]		[3, 4, 18, 24, 25, 32, 48]
Drug response		[22]				[4, 10, 17, 18, 36, 42]
In vitro cell assays		[22, 24, 25]		[34]		[10, 32]
Morphological changes		[22]		[34]		[24, 25]
Differentiation capacity				[21, 34]		[2, 4]
Marker expression (RNA sequencing)						[24, 25, 35, 36]
Marker expression (IF imaging)	[37]					[28, 35]
Electrophysiological analysis						[4, 18, 36]
Marker expression (Flow cytometry)	[47]				[35]	
Substrate binding	[47]					
Cell migration				[21]		
Cell proliferation						[37]
Mechanical properties						[18]
Aggregation assay						[47]
Neurotransmitter uptake						[10]

IF: immunofluorescence.

Table S9. Stability tests of PSC-derived therapy products and their ranking, related to Figure S4.

**Stability aspect**	**Method**	**Ranks and Reference numbers**					
		**QC**	**QCu**	**FIO**	**pQC**	**pFIO**	**Ch**
Prolonged culture	Viability						[17]
	Marker expression						[17]
	Genomic stability			[5]			
In-use stability	Viability						[4, 17, 25, 26, 36]
	Marker expression						[26, 46]
	Morphology						[26, 47]
	Cell count	[47]					
	Appearance	[47]					
	pH	[47]					
	Proliferation						[26]
	Potency						[47]
Cryopreservation	Viability						[4, 5, 25, 36, 48]
	Marker expression						[4, 5, 36, 48]
	Morphology						[36, 48]
	Potency						[29, 48]
	Appearance						[5]
	Cell count						[5]
	Genomic stability						[4]
Delivery device	Viability						[4, 5, 36]
	Marker expression						[36]
	Cell count						[5]
	In vivo						[5]

Table S10. Non-clinical biodistribution studies of PSC-derived therapy products, related to Figure 4 and Figures S5-S6.

Study	Animal model		Reference numbers
Biodistribution	Species	Mice (immunodeficient)	[1, 10, 35, 47]
		Mice (WT)	[25]
		Rats (immunodeficient)	[3, 5, 7]
		Rabbits	[24, 36, 47]
	Male/Female ratio	Both	[3, 5, 24]
		Only male	[25, 36, 47]
		Only female	[7]
		Unspecified	[1, 10, 35, 47]
	Immunosuppressive treatment	Yes	[3, 7, 24, 47]
		No/Unspecified	[1, 5, 10, 25, 35, 36, 47]
	**Dosing**		**Reference numbers**
	Number of doses	Dose-escalation	[7]
		Single dose	[1, 3, 5, 10, 24, 25, 35, 36, 47]
		Repeated dose	-
		Unspecified	-
	Control groups	Positive	[35, 47]
		Negative	[3, 5, 7, 10, 24, 25, 36, 47]
		Spike-in	-
		None/Unspecified	[1]
	Transplantation site	Clinically intended	[1, 3, 5, 7, 10, 24, 25, 35, 36, 47]
		Other	-
	**Study conditions**		**Reference numbers**
	GLP study	Yes	[3, 5, 7, 10]
		No/Unspecified	[1, 24, 25, 35, 36, 47]
	Outsourced	Yes	[3, 5, 7, 10]
		No/Unspecified	[1, 24, 25, 35, 36, 47]

GLP: good laboratory practice, WT: wild-type.

Table S11. Non-clinical tumorigenicity studies of PSC-derived therapy products, related to Figure 4 and Figures S5-S6.

Study	Animal model		Reference numbers
Tumorigenicity	Species	Mice (immunodeficient)	[1, 10, 18, 19, 25, 35-38, 40, 44, 45, 48]
		Rats (immunodeficient)	[2, 3, 5, 7, 26]
		Non-human primates	[17, 44, 45]
		Chick embryo	[38]
	Male/Female ratio	Both	[3, 5, 17, 44]
		Only male	[25, 36, 45]
		Only female	[7, 18, 26, 38]
		Unspecified	[1, 2, 10, 19, 35, 37, 38, 40, 44, 48]
	Immunosuppressive treatment	Yes	[3, 7, 17, 44, 45]
		No/Unspecified	[1, 2, 5, 10, 18, 19, 25, 26, 35-38, 40, 44, 45, 48]
	**Dosing**		**Reference numbers**
	Number of doses	Dose-escalation	[7, 44, 45]
		Single dose	[1-3, 5, 10, 17-19, 26, 35-37, 44, 45]
		Repeated dose	-
		Unspecified	[25, 38, 40, 48]
	Control groups	Positive	[3, 5, 18, 36, 37, 40, 44, 45]
		Negative	[3, 5, 7, 10, 17, 26, 37, 44, 45]
		Spike-in	[3, 35]
		None/Unspecified	[1, 2, 19, 25, 38, 44, 48]
	Transplantation site	Clinically intended	[1-3, 5, 7, 10, 17-19, 26, 35, 40, 44, 45]
		Subcutaneous	[36-38]
		Kidney capsule	[44, 45]
		Testes	[25]
		Chick embryo chorioal lantoic membrane (CAM)	[38]
		Unspecified	[48]
	**Study conditions**		**Reference numbers**
	GLP study	Yes	[3, 5, 7, 10, 22, 40]
		No/Unspecified	[1, 2, 17-19, 25, 26, 35-38, 44, 45, 48]
	Outsourced	Yes	[3, 5, 7, 10]
		No/Unspecified	[1, 2, 17-19, 25, 26, 35-38, 40, 44, 45, 48]

GLP: good laboratory practice, WT: wild-type.

Table S12. Non-clinical toxicity studies of PSC-derived therapy products, related to Figure 4 and Figures S5-S6.

Study	Animal model		Reference numbers
Toxicity	Species	Mice (immunodeficient)	[10, 18, 22, 46]
		Rats (immunodeficient)	[3, 5, 7]
		Rats (WT)	[46]
		Non-human primates	[17, 24, 25]
		Rabbits	[24]
	Male/Female ratio	Both	[3, 5, 17, 18, 24, 25, 46]
		Only male	-
		Only female	[7]
		Unspecified	[10, 22]
	Immunosuppressive treatment	Yes	[7, 17]
		No/Unspecified	[3, 5, 10, 18, 22, 24, 25, 46]
	**Dosing**		**Reference numbers**
	Number of doses	Dose-escalation	[3, 7, 17, 24, 25]
		Single dose	[5, 10, 18, 24, 46]
		Repeated dose	[24, 25, 46]
		Unspecified	[22]
	Control groups	Positive	-
		Negative	[3, 5, 7, 10, 17, 18, 24, 25, 46]
		Spike-in	-
		None/Unspecified	[22]
	Transplantation site	Clinically intended	[3, 5, 7, 10, 17, 18, 24, 25, 46]
		Intravenous	[24]
		Unspecified	[22]
	**Study conditions**		**Reference numbers**
	GLP study	Yes	[3, 5, 7, 10]
		No/Unspecified	[17, 18, 22, 24, 25, 46]
	Outsourced	Yes	[3, 5, 7, 10, 25]
		No/Unspecified	[17, 18, 22, 24, 46]

GLP: good laboratory practice, WT: wild-type.

Table S13. Non-clinical efficacy studies of PSC-derived therapy products, related to Figure 4 and Figures S5-S6.

Study	Animal model		Reference numbers
Efficacy	Species	Mice (disease model)	[1, 8, 10, 20, 25, 32, 44, 45, 48]
		Mice (healthy)	[8, 17, 35, 44]
		Rats (disease model)	[3-5, 7, 10, 22, 26, 29]
		Rats (healthy)	[2]
		Rabbits	[24, 36, 46]
		Non-human primates	[17, 45]
		Pigs	[18]
	Male/Female ratio	Both	[4, 10, 17, 26, 29, 32]
		Only male	[8, 17, 20, 25, 36, 45, 48]
		Only female	[3, 5, 7, 18, 22]
		Unspecified	[1, 2, 24, 35, 44, 46]
	Immunosuppressive treatment	Yes	[3, 4, 7, 10, 17, 18, 24, 29, 36, 45, 46]
		No/Unspecified	[1, 2, 5, 8, 10, 17, 20, 22, 25, 26, 32, 35, 44, 45, 48]
	**Dosing**		**Reference numbers**
	Number of doses	Dose-escalation	[3, 5, 8, 17, 25, 29, 44, 45]
		Single dose	[1, 2, 4, 7, 10, 17, 18, 20, 22, 24, 26, 32, 35, 36, 45, 46, 48]
		Repeated dose	[10, 25, 32]
		Unspecified	-
	Control groups	Positive	[22, 24, 25, 32, 35, 48]
		Negative	[3-5, 7, 8, 10, 17, 18, 20, 22, 25, 26, 29, 32, 36, 44, 45, 48]
		Spike-in	-
		None/Unspecified	[1, 2, 46]
	Transplantation site	Clinically intended	[1-5, 7, 8, 10, 17, 18, 20, 22, 24-26, 29, 32, 35, 36, 45, 46]
		Kidney capsule	[44, 45]
		Intrasplenic	[48]
	**Study conditions**		**Reference numbers**
	GLP study	Yes	[36]
		No/Unspecified	[1-5, 7, 8, 10, 17, 18, 20, 22, 24-26, 29, 32, 35, 44-46, 48]
	Outsourced	Yes	[36, 45]
		No/Unspecified	[1-5, 7, 8, 10, 17, 18, 20, 22, 24-26, 29, 32, 35, 44, 46, 48]

GLP: good laboratory practice, WT: wild-type.

Table S14. CQAs tested and methods applied for clinical release testing of PSC-derived medicinal products, based on methods scored as QC and QCu.

QC	CQA	Method	Ref. no.
Identity	Marker expression	Flow cytometry	[3, 5, 6, 9, 18, 22, 24, 25, 37, 44, 45, 47]
		HLA expression (Flow cytometry)	[22, 24, 25]
		IF imaging	[6, 37]
		qPCR	[6, 27]
		PCR	[3]
	Morphology	Microscopic evaluation	[22, 25, 44, 45]
	Physiological	Differentiation potential	[22, 24]
		Dithizone (DTZ) staining	[44]
		Annexin V binding (Flow cytometry)	[47]
	Genotype	STR analysis	[5, 25, 44]
Impurities	Off-target cells	Flow cytometry	[3, 5, 6, 22, 24, 25, 44, 45, 47]
		qPCR	[6, 26]
		IF imaging	[6]
	Residual PSCs	Flow cytometry	[5, 9, 22, 25]
		qPCR	[3, 27]
		ddPCR	[37, 44]
		IF imaging	[25]
		PCR	[25]
		In vivo (mice)	[25]
	Transformed cells	SACF assay	[44]
	Residual raw materials	BSA	[25, 44, 47]
		Growth factors	[44]
		Small molecules	[47]
		Cytokines	[47]
	Interspecies contamination	STR analysis	[44]
		Isozyme analysis	[25]

Content	Viability	Automated cell counter	[3, 5, 9, 44]
		Fluorescence microscopy	[44]
		Flow cytometry	[45]
		Counting method unspecified	[18, 37]
	Cell count	Automated cell counter	[3, 5, 9]
		Flow cytometry	[47]
		Counting method unspecified	[37]
	General appearance	Visual inspection	[5, 37, 47]
	pH	Electrode method	[47]
Safety	Genomic integrity	Karyotyping	[22]
		WGS	[44]
		WES	[44]
	Adventitious virus	In vitro cell culture	[3, 5, 25, 44]
		Hemadsorption	[25, 44]
		Hemagglutination	[25, 44]
		qPCR (PCR-fluorescence probing)	[44]
		Ultracentrifugation and QFPERT assay	[5]
		In vivo	[25, 44]
		Method unspecified	[25, 45]
Potency	Hormone secretion	ELISA	[9, 44, 45]
	In vitro cell assays	Inhibition of PBMC proliferation	[24, 25]
		Dye transfer assay	[22]
	Marker expression	Flow cytometry	[47]
		IF imaging	[37]
	Morphological changes	In vitro tube formation assay	[22]
	Drug response	In vitro cell assay	[22]
	Substrate binding	Flow cytometry	[47]
Stability	In-use stability	Cell count	[47]
		General appearance	[47]
		pH	[47]
In vivo	Tumorigenicity (mice)	Gross organ observation	[19, 44]
		H&E staining	[19, 44]

HLA: human leukocyte antigen, H&E: hematoxylin and eosin, IF: immunofluorescence, PBMC: peripheral blood mononuclear cell, QFPERT: quantitative fluorescent product-enhanced reverse transcriptase, SACF: soft agar colony formation, STR: short tandem repeat.

Table S15. Tests applied in QC testing of PSC-derived cell therapy products grouped by cell type and specifications provided in the included reports. See Excel file.

Supplemental methods

A systematic search was conducted using pre-defined keywords to retrieve records on PSC-derived products and their QC, safety, and efficacy testing, manufacturing for clinical application, and regulatory guidelines (see section 'Systematic search strategy' below). Three databases were searched spanning the period from 1 January 2022 until 14 November 2024 (Figure S7). Titles and abstracts were screened on EndNote 21 by at least two reviewers independently: one primary reviewer screened the full set of retrieved records, followed by a parallel independent screening by one of the three secondary reviewers. Pre-defined criteria were used to include records on PSC-derived medicinal products developed for clinical application (for example, working with a clinical-grade cell line, implementing a GMP-compliant protocol, or mentioning the conduct of a clinical trial) and to exclude papers with a primary focus on adult stem cell products, exosomes, disease modelling, drug screening, developmental studies, or novel/pioneering techniques under development (see section 'Pre-defined inclusion/exclusion criteria' below). For the full text screening, reviews were removed first, and the remaining original publications were screened by the same primary and secondary reviewers. Four reports could not be retrieved because the trial registry records did not contain details about the quality testing. After exclusion due to the specified reasons, 41 reports remained.

To ensure the completeness of the retrieved reference list, the review from Kirkeby et al., 2025 [51] was used for cross-reference. Out of 61 listed clinical and non-clinical references from 2022-2024, 4 reports were included in the analysis after full-text screening [6, 8, 17, 32] (Figure S7). In addition, one relevant report was found independently (did not contain an abstract) [45] and 4 reports were added manually as specifically cross-referenced by the included articles to contain relevant additional information on the products [7, 10, 25, 29]. Finally, a total of 50 reports were included in the analysis.

The data extraction from the included articles was performed by the primary reviewer in close collaboration with the secondary reviewers. The data of interest were: (1) general information about the product and initiation of a clinical trial; (2) quality tests for product's identity, purity, content, safety, potency, and stability; and (3) *in vivo* studies for biodistribution, tumorigenicity, toxicity, and efficacy. Only the tests performed on end-differentiated cell products were extracted for this review. For the *in vitro* testing, the information of interest comprised of the utilized techniques and sensitivity, biomarkers, and source material of selected methods. For the *in vivo* studies, data were collected on the animal model (species, animal number, follow-up duration, sex, use of immunosuppression), dosing regimen

(number of doses, control groups, administration site), and study conditions (GLP, outsourcing). A ranking system was created to define the application of product quality tests across different stages of product development and clinical release (Table 1). The ranking was assigned based on the implementation of a testing method as described in a report. Similarly, the assignment of the test to a certain category were determined based on the primary goal of the testing method as defined in a report, for example, the test for detecting potentially tumorigenic impurities was categorized as impurity testing, while tests evaluating tumor formation as a safety risk fell under safety.

The relevant available information was extracted into an Excel sheet, summarized in table format, and subsequently transformed into graphical representations using GraphPad Prism 10.2.3. In cases where the *in vitro* or *in vivo* testing of the same medicinal product intended for the same indication was described in multiple reports, the test or study was counted once at the highest applied rank. There were six medicinal products covered by double reports among the included articles (Table S16), which usually was a result of separate publications on pre-clinical and clinical study reports. Durations of longest follow-up time points of *in vivo* studies specified in hours, days, weeks, or years were converted to months using an online tool Omni Calculator [52]. The relevant information was extracted where possible, and any missing or incomplete information (e.g. animal number or duration of *in vivo* studies) was treated as not given. Any differences in opinion during the screening and data extraction were resolved by consensus or referral to a third reviewer.

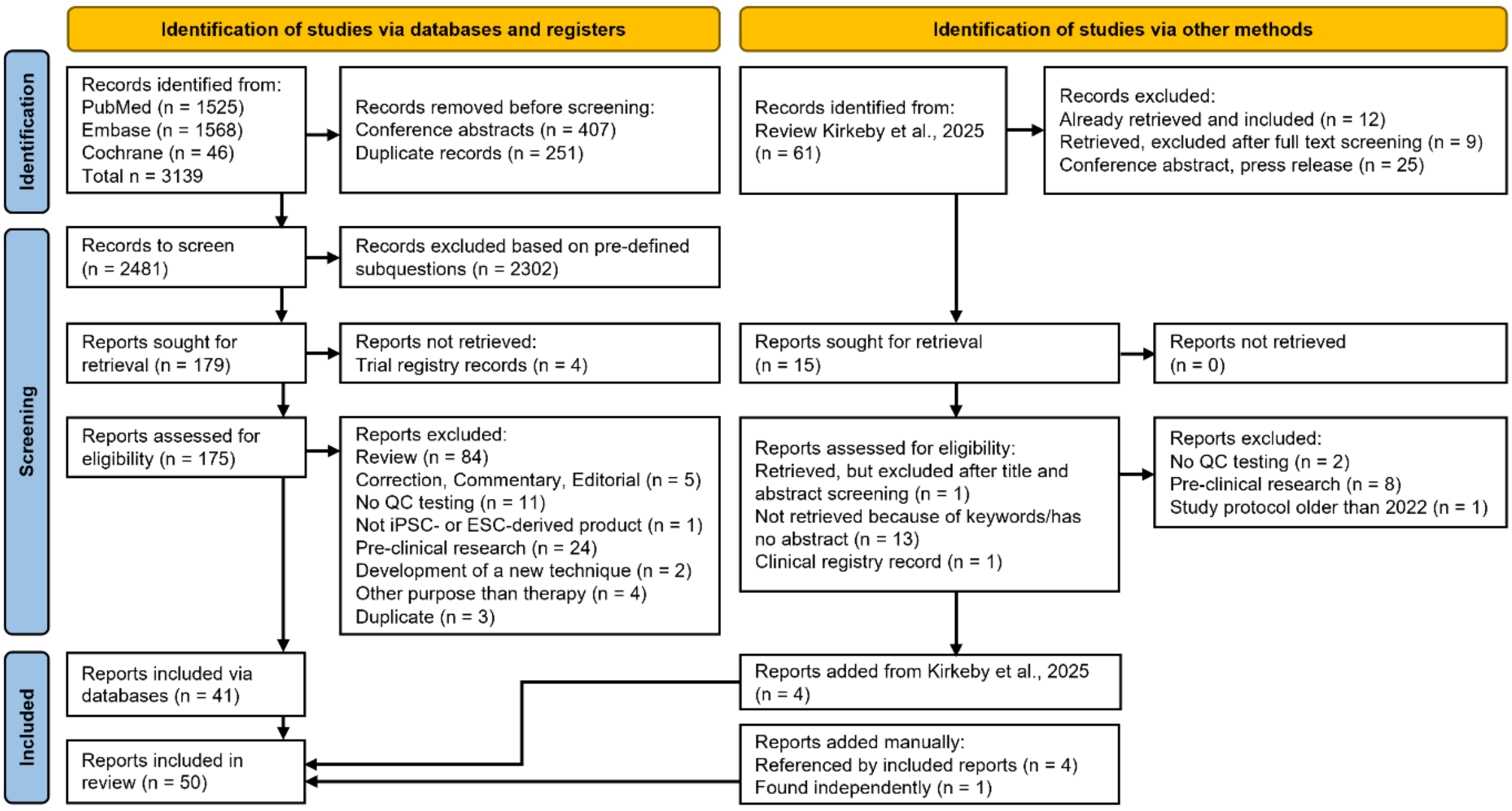

Figure S7. PRISMA 2020 flow diagram. The reports were collected via a systematic search and screening of databases, cross-referenced review paper, and manual addition. See also Supplemental methods.

Table S16. Medicinal products intended for the same indication covered by double reports. In the cases when a test or study was described in both reports, it was counted once at the highest applied rank (e.g. a test performed both as a characterization test during pre-clinical development, and in a later stage as a QC test with defined release specifications, this test was ranked as a QC test).

**Medicinal products covered by double reports**	**Reference numbers**
Cardiomyocytes (patches)	[18, 19]
Photoreceptors (retinal sheets)	[26, 27]
Astrocytes (AstroRx)	[9, 10]
RPE cells (suspension)	[28, 29]
Platelets (iPLAT1 clinical trial)	[46, 47]
Oligodendrocyte progenitor cells	[6, 7]

Systematic search strategy

<u>PubMed:</u>

("Stem cell-derived"[ti] OR "PSC-derived"[ti] OR "hPSC-derived"[ti] OR "iPSC-derived"[ti] OR "hiPSC-derived"[ti] OR "ESC-derived"[ti] OR "hESC-derived"[ti] OR "from pluripotent stem cell*"[tiab] OR "from human pluripotent stem cell*"[tiab] OR "from induced pluripotent stem cell*"[tiab] OR "from human induced pluripotent stem cell*"[tiab] OR "from embryonic stem cell*"[tiab] OR "from human embryonic stem cell*"[tiab] OR "from PSCs"[tiab] OR "from hPSCs"[tiab] OR "from human PSCs"[tiab] OR "from iPSCs"[tiab] OR "from hiPSCs"[tiab] OR "from human iPSCs"[tiab] OR "from ESCs"[tiab] OR "from hESCs"[tiab] OR "from human ESCs"[tiab] OR "residual stem cell*"[tiab] OR "residual PSC*"[tiab] OR "residual hPSC*"[tiab] OR "residual human PSC*"[tiab] OR "residual iPSC*"[tiab] OR "residual hiPSC*"[tiab] OR "residual human iPSC*"[tiab] OR "residual ESC*"[tiab] OR "residual hESC*"[tiab] OR "residual human ESC*"[tiab] OR "residual undifferentiated"[tiab])

AND

("Quality Control"[MeSH] OR "Total Quality Management"[MeSH] OR "Quality"[tiab] OR "QC test*"[tiab] OR "Chemistry, manufacturing and control"[tiab] OR "In-process control*"[tiab] OR "Good Manufacturing Practice"[tiab] OR "Clinical-grade"[tiab] OR "GMP-grade"[tiab] OR "GMP-compliant"[tiab] OR "Manufacturing"[tiab] OR "Product releas*"[tiab] OR "Process development"[tiab] OR ("Clinical"[tiab] AND "Trial*"[tiab]) OR ("Clinical"[tiab] AND "Stud*"[tiab]) OR "Consumer Product Safety"[MeSH] OR "Safety"[MeSH] OR "Safety"[tiab] OR "Carcinogenicity Tests"[MeSH] OR "Carcinogen*"[tiab] OR "Tumorigen*"[tiab] OR "Terato*"[tiab] OR "Genomic integrity"[tiab] OR "Genomic stability"[tiab] OR "Genomic instability"[tiab] OR "Efficac*"[tiab] OR "Potenc*"[tiab] OR "Characteri*"[tiab] OR "Legislation, Drug"[Mesh] OR "Policy"[Mesh] OR "United States Food and Drug Administration"[Mesh] OR "Legislat*"[tiab] OR "Policy"[tiab] OR "Policies"[tiab] OR "Regulation*"[tiab] OR "Guideline*"[tiab] OR "Guidance*"[tiab] OR "Regulatory standard*"[tiab] OR "Regulatory requirement*"[tiab])

Embase OVID:

(*induced pluripotent stem cell/ or *embryonic stem cell/ or ("Stem cell-derived" or "PSC-derived" or "hPSC-derived" or "iPSC-derived" or "hiPSC-derived" or "ESC-derived" or "hESC-derived").ti. or ("from pluripotent stem cell*" or "from human pluripotent stem cell*" or "from induced pluripotent stem cell*" or "from human induced pluripotent stem cell*" or "from embryonic stem cell*" or "from human embryonic stem cell*" or "from PSCs" or "from hPSCs" or "from human PSCs" or "from iPSCs" or "from hiPSCs" or "from human iPSCs" or "from ESCs" or "from hESCs" or "from human ESCs" or "residual stem cell*" or "residual PSC*" or "residual hPSC*" or "residual human PSC*" or "residual iPSC*" or "residual hiPSC*" or "residual human iPSC*" or "residual ESC*" or "residual hESC*" or "residual human ESC*" or "residual undifferentiated").ti,ab.) and (*quality control/ or *total quality management/ or *product safety/ or *patient safety/ or *drug safety/ or *good manufacturing practice/ or exp clinical trial/ or *drug efficacy/ or *drug potency/ or *carcinogen testing/ or *carcinogenesis/ or *carcinogenicity/ or *process development/ or *policy/ or *"food and drug administration"/ or *European Medicines Agency/ or *drug legislation/ or *drug approval/ or *genomic instability/ or ("Quality" or "QC test*" or "Chemistry, manufacturing and control" or "In-process control*" or "Good Manufacturing Practice" or ("Clinical" adj1 "Trial*") or ("Clinical" adj1 "Stud*") or "Safety" or "Efficac*" or "Potenc*" or "Characteri*" or "Carcinogen*" or "Tumorigen*" or "Terato*" or "Clinical-grade" or "GMP-grade" or "GMP-compliant" or "Manufacturing" or "Product releas*" or "Process development" or "Legislat*" or "Regulation*" or "Guideline*" or "Guidance*" or "Policy" or "Policies" or "Regulatory standard*" or "Regulatory requirement*" or "Genomic integrity" or "Genomic stability" or "Genomic instability").ti.)

Cochrane library:

ID Search

#1 ((pluripotent NEXT stem cell*) OR (induced NEXT pluripotent stem cell*) OR (embryonic NEXT stem cell*) OR (residual NEXT stem cell*) OR (residual NEXT PSC*) OR (residual NEXT hPSC*) OR (residual NEXT human PSC*) OR (residual NEXT human iPSC*) OR (residual NEXT iPSC*) OR (residual NEXT hiPSC*) OR (residual NEXT hESC*) OR (residual NEXT ESC*) OR (residual NEXT human ESC*) OR ("residual undifferentiated")):ti,ab,kw

#2 ("Stem cell-derived" or "PSC-derived" or "hPSC-derived" or "iPSC-derived" or "hiPSC-derived" or "ESC-derived" or "hESC-derived"):ti,ab,kw

#3 MeSH descriptor: [Induced Pluripotent Stem Cells] explode all trees

#4 MeSH descriptor: [Embryonic Stem Cells] explode all trees

#5 MeSH descriptor: [Pluripotent Stem Cells] explode all trees

#6 {OR #1-#5}

#7 MeSH descriptor: [Quality Control] explode all trees

#8 MeSH descriptor: [Total Quality Management] explode all trees

#9 MeSH descriptor: [Consumer Product Safety] explode all trees

#10 MeSH descriptor: [Carcinogenicity Tests] explode all trees

#11 MeSH descriptor: [Carcinogenesis] explode all trees

#12 MeSH descriptor: [Safety] explode all trees

#13 MeSH descriptor: [Legislation, Drug] explode all trees

#14 MeSH descriptor: [Drug Evaluation] explode all trees

#15 MeSH descriptor: [Drug Evaluation, Preclinical] explode all trees

#16 MeSH descriptor: [Policy] explode all trees

#17 MeSH descriptor: [United States Food and Drug Administration] explode all trees

#18 MeSH descriptor: [Clinical Trial] explode all trees

#19 MeSH descriptor: [Genomic Instability] explode all trees

#20 (("Quality" or (QC NEXT test*) or "Chemistry manufacturing and control" or (In-process NEXT control*) or (Good Manufacturing NEXT Practice*) or (Clinical NEXT Trial*) or (Clinical NEXT Stud*) or "Safety" or Efficac* or Potenc* or Characteri* or Carcinogen* or Tumorigen* or "Clinical-grade" or "GMP-grade" or "GMP-compliant" or "Manufacturing" or (Product NEXT releas*) or "Process development" or Legislat* or Regulation* or Guideline* or Guidance* or "Policy" or "Policies" or (Regulatory NEXT standard*) or (Regulatory NEXT requirement*) or "Genomic integrity" or "Genomic stability" OR Terato*)):ti,ab,kw

#21 {OR #7-#20}

#22 #6 AND #21

Pre-defined inclusion/exclusion criteria

Include, if the answer is “Yes”:

1. Does the paper discuss **stem cell-derived** therapy to be used in the clinic (e.g. mentions "clinical-grade", "GMP-compliant", "clinical trial")?
    a. Does the paper discuss **safety** of stem cell-based therapy?
    b. Does the paper discuss the measures to prevent **tumorigenicity** of stem cell-based therapy?
    c. Does the paper mention **biodistribution** studies?
    d. Does the paper mention **cell identity/composition** testing of a cell therapy product?
    e. Does the paper mention **purity** testing of a cell therapy product?
    f. Does the paper mention **potency** testing of a cell therapy product?
    g. Does the paper mention detection/removal of **residual stem cells**?

Exclude, if the answer is “Yes”:

1. Is the main focus of the paper a **stem cell therapy**?
2. Is the main focus of the paper a cell therapy derived from the stem cells other than iPSCs or ESCs (e.g. **mesenchymal stem cell-derived)**?
3. Is the main focus of the paper stem cell-derived **exosomes**?
4. Is the main focus of the paper **disease modelling** (using organoids, organs-on-chips)?
5. Is the main focus of the paper **drug screening** using stem cells or their products?
6. Is the main focus of the paper **developmental studies** (organism/organ development)?
7. Is the main focus of the paper **a novel strategy under development** for the detection/removal of **residual stem cells** that is **pre-clinical**?
8. Is the main focus of the paper **a novel test under development** for a stem cell-derived product that is **pre-clinical**?

Supplemental references

[1] M. Luciani, C. Garsia, E. Mangiameli, V. Meneghini, and A. Gritti, "Intracerebroventricular transplantation of human iPSC-derived neural stem cells (hiPSC-NSCs) into neonatal mice," (in eng), *Methods Cell Biol,* vol. 171, pp. 127-147, 2022, doi: 10.1016/bs.mcb.2022.04.007.

[2] M. Shigyo *et al.*, "Derivation of Sendai-Virus-Reprogrammed Human iPSCs-Neuronal Precursors: In Vitro and In Vivo Post-grafting Safety Characterization," (in English), *Cell Transplantation,* vol. 32, no. no pagination, 2023, doi: https://dx.doi.org/10.1177/09636897231163232.

[3] S. Park *et al.*, "Preclinical and dose-ranging assessment of hESC-derived dopaminergic progenitors for a clinical trial on Parkinson's disease," (in English), *Cell Stem Cell,* vol. 31(1), pp. 25-38.e8, 04 Jan 2024, doi: https://dx.doi.org/10.1016/j.stem.2023.11.009.

[4] S. Naderi *et al.*, "Cryopreserved clinical-grade human embryonic stem cell-derived dopaminergic progenitors function in Parkinson's disease models," (in English), *Life Sciences,* vol. 329(no pagination), 15 Sep 2023, Art no. 121990, doi: https://dx.doi.org/10.1016/j.lfs.2023.121990.

[5] A. Kirkeby *et al.*, "Preclinical quality, safety, and efficacy of a human embryonic stem cell-derived product for the treatment of Parkinson's disease, STEM-PD," (in English), *Cell Stem Cell,* vol. 30(10), pp. 1299-1314.e9, 05 Oct 2023, doi: https://dx.doi.org/10.1016/j.stem.2023.08.014.

[6] R. G. Fessler *et al.*, "A phase 1/2a dose-escalation study of oligodendrocyte progenitor cells in individuals with subacute cervical spinal cord injury," *J Neurosurg Spine,* vol. 37, no. 6, pp. 812-820, Dec 1 2022, doi: 10.3171/2022.5.SPINE22167.

[7] N. C. Manley, C. A. Priest, J. Denham, E. D. Wirth, 3rd, and J. S. Lebkowski, "Human Embryonic Stem Cell-Derived Oligodendrocyte Progenitor Cells: Preclinical Efficacy and Safety in Cervical Spinal Cord Injury," *Stem Cells Transl Med,* vol. 6, no. 10, pp. 1917-1929, Oct 2017, doi: 10.1002/sctm.17-0065.

[8] M. Bershteyn *et al.*, "Human pallial MGE-type GABAergic interneuron cell therapy for chronic focal epilepsy," *Cell Stem Cell,* vol. 30, no. 10, pp. 1331-1350 e11, Oct 5 2023, doi: 10.1016/j.stem.2023.08.013.

[9] M. Gotkine *et al.*, "Safety and efficacy of first-in-man intrathecal injection of human astrocytes (AstroRx) in ALS patients: phase I/IIa clinical trial results," (in English), *Journal of Translational Medicine,* vol. 21(1) (no pagination), December 2023, Art no. 122, doi: https://dx.doi.org/10.1186/s12967-023-03903-3.

[10] M. Izrael *et al.*, "Safety and efficacy of human embryonic stem cell-derived astrocytes following intrathecal transplantation in SOD1(G93A) and NSG animal models," *Stem Cell Res Ther,* vol. 9, no. 1, p. 152, Jun 6 2018, doi: 10.1186/s13287-018-0890-5.

[11] J. W. Park, S. J. Bae, J. H. Yun, S. Kim, and M. Park, "Assessment of Genetic Stability in Human Induced Pluripotent Stem Cell-Derived Cardiomyocytes by Using Droplet Digital PCR," *Int J Mol Sci,* vol. 25, no. 2, Jan 16 2024, doi: 10.3390/ijms25021101.

[12] T. Watanabe *et al.*, "International evaluation study of a highly efficient culture assay for detection of residual human pluripotent stem cells in cell therapies," (in English), *Regenerative Medicine,* vol. 18(3), pp. 219-227, 01 Mar 2023, doi: https://dx.doi.org/10.2217/rme-2022-0207.

[13] O. Sekine *et al.*, "Seamless and non-destructive monitoring of extracellular microRNAs during cardiac differentiation from human pluripotent stem cells," *Stem Cell Reports,* vol. 18, no. 10, pp. 1925-1939, Oct 10 2023, doi: 10.1016/j.stemcr.2023.08.011.

[14] M. Lemmens *et al.*, "Identification of marker genes to monitor residual iPSCs in iPSC-derived products," *Cytotherapy,* vol. 25, no. 1, pp. 59-67, Jan 2023, doi: 10.1016/j.jcyt.2022.09.010.

[15] S. Yasuda *et al.*, "Detection of residual pluripotent stem cells in cell therapy products utilizing droplet digital PCR: an international multisite evaluation study," *Stem Cells Transl Med,* vol. 13, no. 10, pp. 1001-1014, Oct 10 2024, doi: 10.1093/stcltm/szae058.

[16] N. Kriedemann *et al.*, "Standardized production of hPSC-derived cardiomyocyte aggregates in stirred spinner flasks," (in eng), *Nat Protoc,* Mar 28 2024, doi: 10.1038/s41596-024-00976-2.

[17] H. Kobayashi *et al.*, "Regeneration of Nonhuman Primate Hearts With Human Induced Pluripotent Stem Cell-Derived Cardiac Spheroids," *Circulation,* vol. 150, no. 8, pp. 611-621, Aug 20 2024, doi: 10.1161/CIRCULATIONAHA.123.064876.

[18] S. Miyagawa *et al.*, "Pre-clinical evaluation of the efficacy and safety of human induced pluripotent stem cell-derived cardiomyocyte patch," (in English), *Stem Cell Research and Therapy,* vol. 15(1) (no pagination), December 2024, Art no. 73, doi: https://dx.doi.org/10.1186/s13287-024-03690-8.

[19] S. Miyagawa *et al.*, "Case report: Transplantation of human induced pluripotent stem cell-derived cardiomyocyte patches for ischemic cardiomyopathy," *Front Cardiovasc Med,* vol. 9, p. 950829, 2022, doi: 10.3389/fcvm.2022.950829.

[20] L. Rong *et al.*, "Clinical-grade human embryonic stem cell-derived mesenchymal stromal cells ameliorate diabetic retinopathy in db/db mice," (in English), *Cytotherapy.,* 2024, doi: https://dx.doi.org/10.1016/j.jcyt.2024.02.020.

[21] Q. Zhao and F. Liu, "Derivation and Characterization of Mesenchymal Stem Cells from iPS Cells," (in English), *Methods in Molecular Biology,* Chapter vol. 2454, pp. 443-454, 2022, doi: https://dx.doi.org/10.1007/7651_2020_327.

[22] J. H. Shin *et al.*, "Safety of Human Embryonic Stem Cell-derived Mesenchymal Stem Cells for Treating Interstitial Cystitis: A Phase I Study," (in English), *Stem Cells Translational Medicine,* vol. 11(10), pp. 1010-1020, October 2022, doi: https://dx.doi.org/10.1093/stcltm/szac065.

[23] T. Watanabe *et al.*, "Multisite studies for optimization of a highly efficient culture assay used for in vitro detection of residual undifferentiated human pluripotent stem cells intermingled in cell therapy products," *Regen Ther,* vol. 26, pp. 315-323, Jun 2024, doi: 10.1016/j.reth.2024.06.007.

[24] L. Huang *et al.*, "Immunity-and-matrix-regulatory cells enhance cartilage regeneration for meniscus injuries: a phase I dose-escalation trial," (in English), *Signal Transduction and Targeted Therapy,* vol. 8(1) (no pagination), December 2023, Art no. 417, doi: https://dx.doi.org/10.1038/s41392-023-01670-7.

[25] J. Wu *et al.*, "Immunity-and-matrix-regulatory cells derived from human embryonic stem cells safely and effectively treat mouse lung injury and fibrosis," *Cell Res,* vol. 30, no. 9, pp. 794-809, Sep 2020, doi: 10.1038/s41422-020-0354-1.

[26] K. Watari *et al.*, "Self-organization, quality control, and preclinical studies of human iPSC-derived retinal sheets for tissue-transplantation therapy," (in English), *Communications biology,* vol. 6(1), p. 164, 10 Feb 2023, doi: https://dx.doi.org/10.1038/s42003-023-04543-5.

[27] Y. Hirami *et al.*, "Safety and stable survival of stem-cell-derived retinal organoid for 2 years in patients with retinitis pigmentosa," (in English), *Cell Stem Cell,* vol. 30(12), pp. 1585-1596.e6, 07 Dec 2023, doi: https://dx.doi.org/10.1016/j.stem.2023.11.004.

[28] H. Surendran *et al.*, "An improved protocol for generation and characterization of human-induced pluripotent stem cell-derived retinal pigment epithelium cells," (in English), *STAR protocols,* vol. 3(4), p. 101803, 16 Dec 2022, doi: https://dx.doi.org/10.1016/j.xpro.2022.101803.

[29] H. Surendran *et al.*, "Transplantation of retinal pigment epithelium and photoreceptors generated concomitantly via small molecule-mediated differentiation rescues visual function in rodent models of retinal degeneration," *Stem Cell Res Ther,* vol. 12, no. 1, p. 70, Jan 19 2021, doi: 10.1186/s13287-021-02134-x.
[30] H. Y. Jo *et al.*, "Single-Cell RNA Sequencing of Human Pluripotent Stem Cell-Derived Macrophages for Quality Control of The Cell Therapy Product," (in English), *Frontiers in Genetics,* vol. 12(no pagination), 31 Jan 2022, Art no. 658862, doi: https://dx.doi.org/10.3389/fgene.2021.658862.
[31] L. Chung, L. A. Cogburn, L. Sui, and J. L. Dashnau, "Development of an induced pluripotent stem cell-specific microRNA assay for detection of residual undifferentiated cells in natural killer cell therapy products," *Cytotherapy,* vol. 24, no. 7, pp. 733-741, Jul 2022, doi: 10.1016/j.jcyt.2022.02.005.
[32] F. Cichocki *et al.*, "Quadruple gene-engineered natural killer cells enable multi-antigen targeting for durable antitumor activity against multiple myeloma," *Nat Commun,* vol. 13, no. 1, p. 7341, Nov 29 2022, doi: 10.1038/s41467-022-35127-2.
[33] J. Sun *et al.*, "Analysis of three characterization assays reveals ddPCR of LIN28A as the most sensitive for the detection of residual pluripotent stem cells in cellular therapy products," *Cytotherapy,* vol. 26, no. 11, pp. 1374-1381, Nov 2024, doi: 10.1016/j.jcyt.2024.05.019.
[34] S. Domingues *et al.*, "Clinical Grade Human Pluripotent Stem Cell-Derived Engineered Skin Substitutes Promote Keratinocytes Wound Closure In Vitro," (in English), *Cells,* vol. 11(7) (no pagination), April-1 2022, Art no. 1151, doi: https://dx.doi.org/10.3390/cells11071151.
[35] G. Neumayer *et al.*, "A scalable and cGMP-compatible autologous organotypic cell therapy for Dystrophic Epidermolysis Bullosa," *Nat Commun,* vol. 15, no. 1, p. 5834, Jul 11 2024, doi: 10.1038/s41467-024-49400-z.
[36] J. Yu *et al.*, "Safety and efficacy of human ESC-derived corneal endothelial cells for corneal endothelial dysfunction," (in English), *Cell and Bioscience,* vol. 13(1) (no pagination), December 2023, Art no. 201, doi: https://dx.doi.org/10.1186/s13578-023-01145-w.
[37] T. Soma *et al.*, "Induced pluripotent stem-cell-derived corneal epithelium for transplant surgery: a single-arm, open-label, first-in-human interventional study in Japan," (in eng), *Lancet,* Nov 7 2024, doi: 10.1016/s0140-6736(24)01764-1.
[38] M. Corsini and S. Mitola, "Alternative In Vivo Models to Study Teratoma," (in English), *Methods in Molecular Biology,* Chapter vol. 2572, pp. 181-189, 2023, doi: https://dx.doi.org/10.1007/978-1-0716-2703-7_14.
[39] H. Shi, T. Feng, R. Wang, L. Wu, and Y. Gu, "Universal Markers for hiPSCs Residue Detection," *Front Biosci (Landmark Ed),* vol. 27, no. 8, p. 239, Aug 11 2022, doi: 10.31083/j.fbl2708239.
[40] S. Oh *et al.*, "Tumorigenicity Assessment of Human Cancer Cell Lines Xenografted on Immunodeficient Mice as Positive Controls of Tumorigenicity Testing," (in English), *International Journal of Toxicology,* vol. 41(6), pp. 476-487, December 2022, doi: https://dx.doi.org/10.1177/10915818221124573.
[41] K. Masumoto *et al.*, "Highly sensitive and non-disruptive detection of residual undifferentiated cells by measuring miRNAs in culture supernatant," (in eng), *Sci Rep,* vol. 12, no. 1, p. 10351, Jun 20 2022, doi: 10.1038/s41598-022-14273-z.
[42] D. G. Iworima *et al.*, "Metabolic switching, growth kinetics and cell yields in the scalable manufacture of stem cell-derived insulin-producing cells," (in eng), *Stem Cell Res Ther,* vol. 15, no. 1, p. 1, Jan 2 2024, doi: 10.1186/s13287-023-03574-3.
[43] Y. Wu, Z. Zhang, S. Wu, Z. Chen, and Y. Pu, "Estimating residual undifferentiated cells in human chemically induced pluripotent stem cell derived islets using lncRNA as

biomarkers," *Sci Rep,* vol. 13, no. 1, p. 16435, Sep 30 2023, doi: 10.1038/s41598-023-43798-0.

[44] S. Wang *et al.*, "Transplantation of chemically induced pluripotent stem-cell-derived islets under abdominal anterior rectus sheath in a type 1 diabetes patient," (in eng), *Cell,* vol. 187, no. 22, pp. 6152-6164.e18, Oct 31 2024, doi: 10.1016/j.cell.2024.09.004.

[45] J. Wu *et al.*, "Treating a type 2 diabetic patient with impaired pancreatic islet function by personalized endoderm stem cell-derived islet tissue," *Cell Discov,* vol. 10, no. 1, p. 45, Apr 30 2024, doi: 10.1038/s41421-024-00662-3.

[46] N. Sugimoto *et al.*, "Production and nonclinical evaluation of an autologous iPSC-derived platelet product for the iPLAT1 clinical trial," (in English), *Blood Advances,* vol. 6(23), pp. 6056-6069, 13 Dec 2022, doi: https://dx.doi.org/10.1182/bloodadvances.2022008512.

[47] N. Sugimoto *et al.*, "iPLAT1: the first-in-human clinical trial of iPSC-derived platelets as a phase 1 autologous transfusion study," (in English), *Blood,* vol. 140(22), pp. 2398-2402, 01 Dec 2022, doi: https://dx.doi.org/10.1182/blood.2022017296.

[48] M. Gantier *et al.*, "Cryopreserved cGMP-compliant human pluripotent stem cell-derived hepatic progenitors rescue mice from acute liver failure through rapid paracrine effects on liver cells," (in eng), *Stem Cell Res Ther,* vol. 15, no. 1, p. 71, Mar 12 2024, doi: 10.1186/s13287-024-03673-9.

[49] H. Tsujimoto *et al.*, "In vitro methods to ensure absence of residual undifferentiated human induced pluripotent stem cells intermingled in induced nephron progenitor cells," (in eng), *PLoS One,* vol. 17, no. 11, p. e0275600, 2022, doi: 10.1371/journal.pone.0275600.

[50] M. E. Diaz-Hernandez, N. M. Khan, and H. Drissi, "Efficient Differentiation of Human Induced Pluripotent Stem Cell (hiPSC)-Derived Mesenchymal Progenitors Into Adipocytes and Osteoblasts," (in eng), *Bio Protoc,* vol. 13, no. 22, p. e4885, Nov 20 2023, doi: 10.21769/BioProtoc.4885.

[51] A. Kirkeby, H. Main, and M. Carpenter, "Pluripotent stem-cell-derived therapies in clinical trial: A 2025 update," *Cell Stem Cell,* vol. 32, no. 1, pp. 10-37, Jan 2 2025, doi: 10.1016/j.stem.2024.12.005.

[52] "Omni Calculator." https://www.omnicalculator.com/ (accessed [Accessed August 7, 2025.
